# Targeting DNA Binding for NF-κB as an Anticancer Approach in Hepatocellular Carcinoma

**DOI:** 10.3390/cells7100177

**Published:** 2018-10-22

**Authors:** Po Yee Chung, Pik Ling Lam, Yuanyuan Zhou, Jessica Gasparello, Alessia Finotti, Adriana Chilin, Giovanni Marzaro, Roberto Gambari, Zhaoxiang Bian, Wai Ming Kwok, Wai Yeung Wong, Xi Wang, Alfred King-yin Lam, Albert Sun-chi Chan, Xingshu Li, Jessica Yuen Wuen Ma, Chung Hin Chui, Kim Hung Lam, Johnny Cheuk On Tang

**Affiliations:** 1 State Key Laboratory of Chemical Biology and Drug Discovery, Department of Applied Biology and Chemical Technology, The Hong Kong Polytechnic University, Hong Kong, China; chung.poyee@connect.polyu.hk (P.Y.C.); tcstacey@polyu.edu.hk (P.L.L.); yuanyuan.09.zhou@connect.polyu.hk (Y.Z.); bcwmkwok@polyu.edu.hk (W.M.K.); wyrwong@polyu.edu.hk (W.Y.W.); wangxi_1993@126.com (X.W.); chui.ch@gmail.com (C.H.C.); 2 Department of Life Sciences and Biotechnology, University of Ferrara, 44121 Ferrara, Italy; jessica.gasparello@unife.it (J.G.); alessia.finotti@unife.it (A.F.); 3 Department of Pharmaceutical and Pharmacological Sciences, University of Padova, 35122 Padova, Italy; adriana.chilin@unipd.it (A.C.); giovanni.marzaro@unipd.it (G.M.); 4 Clinical Division, School of Chinese Medicine, Hong Kong Baptist University, Hong Kong, China; bzxiang@hkbu.edu.hk; 5 Griffith Medical School, Griffith University, Gold Coast, QLD 4222, Australia; A.Lam@griffith.edu.au; 6 School of Pharmaceutical Sciences, Sun Yat-sen University, Guangzhou 510006, China; chenxz3@mail.sysu.edu.cn (A.S.-c.C.); lixsh@mail.sysu.edu.cn (X.L.); 7 School of Optometry, The Hong Kong Polytechnic University, Hong Kong, China; jessicayw.ma@polyu.edu.hk (J.Y.W.M.)

**Keywords:** NF-κB, anticancer, hepatocellular carcinoma, quinolines

## Abstract

Quinoline core has been shown to possess a promising role in the development of anticancer agents. However, the correlation between its broad spectrum of bioactivity and the underlying mechanism of actions is poorly understood. The present study, with the use of bioinformatics approaches, reported a series of designed molecules which integrated quinoline core and sulfonyl moiety, with the objective of evaluating the substituent and linker effects on anticancer activities and associated mechanistic targets. We identified potent compounds (**1h**, **2h**, **5** and **8**) exhibiting significant anticancer effects towards liver cancer cells (Hep3B) with the 3-(4,5-dimethylthiazol-2-yl)-5-(3-carboxymethoxyphenyl)-2-(4-sulfophenyl)-2*H*-tetrazolium (MTS) relative values of cytotoxicity below 0.40, a value in the range of doxorubicin positive control with the value of 0.12. Bulky substituents and the presence of bromine atom, as well as the presence of sulfonamide linkage, are likely the favorable structural components for molecules exerting a strong anticancer effect. To the best of our knowledge, our findings obtained from chemical synthesis, in vitro cytotoxicity, bioinformatics-based molecular docking analysis (similarity ensemble approach, SEA),and electrophoretic mobility shift assay provided the first evidence in correlation to the anticancer activities of the selected compound **5** with the modulation on the binding of transcription factor NF-κB to its target DNA. Accordingly, compound **5** represented a lead structure for the development of quinoline-based NF-κB inhibitors and this work added novel information on the understanding of the mechanism of action for bioactive sulfonyl-containing quinoline compounds against hepatocellular carcinoma.

## 1. Introduction

Cancer is one of the most life threatening diseases worldwide [1]. Thus, continuous development of effective anticancer drugs with the emergence of drug resistance to existing anticancer agents remains a big challenge [2]. Research work for isolating and modifying natural alkaloids from plants and microorganisms can help scientists probe for more biological active leads [3,4,5]. Natural alkaloids, such as derivatives of quinolines, are classes of nitrogen-containing heterocycles, are one of the widest classes of natural products and originate from either natural or artificially synthesized sources [6]. Some quinoline compounds and their derivatives are available as drugs nowadays [7]; examples include (but are not limited to) antimalarial (e.g., quinine, quinidine, chloroquine), antiviral (e.g., saquinavir), antibacterial (e.g., ciprofloxacin, sparfoxacin and gatifloxacin), antifungal and antiprotozoal (e.g., clioquinol), and anticancer (e.g., camptothecin, irinotecan and cabozantinib). Quinoline scaffold has been recognized as an important source of anticancer drugs as they showed different effects on cancer biological functions, such as growth inhibition associated with cell cycle arrest, apoptosis, inhibition of angiogenesis, arrest of cell migration, and modulation of nuclear receptor responsiveness [7]. More recently, Hu et al. [8] reported a tetraisohydroquinoline-based compound which could enhance the antitumor efficiency in human cancer cell lines. Therefore, there is a continuous research need to isolate and modify naturalalkaloids as biological active leads [7,9,10]. In particular, tropical plants, such as *Galipea officinalis* and *Galipea longiflora*, are naturally occurring sources of several important tetrahydroquinoline and quinoline alkaloids [11]. Extensive studies, including our reported work, demonstrated that quinoline derivatives have a wide range of biological activities [12] leading, for instance, to anticancer [13], antimicrobial [14], and anti-inflammatory effects [15].

Hepatocellular carcinoma (HCC) is one of the leading causes of cancer-related death worldwide; the survival rate remains low (about 20%), even after the introduction of physical treatments, such as liver transplant surgery and radiation and chemotherapy [16,17]. However, drugs with optimal efficacy for HCC are still limited, and it has been suggested that the targeted regulation involving the inhibition on cell migration and epithelia-mesenchymal transition (EMT) processes might be the new directions for cancer treatment [18]. Thus, development of novel small-molecule drugs with better potency and limited side effects could help improve survival rate of patients with HCC.

In the present study, we reported a series of designed molecules that integrated quinoline core and sulfonyl moiety, with the objective of evaluating the substituent and linker effects on anticancer activities in HCC cells and the associated putative mechanistic target, the transcription factor NF-κB, with bioinformatic, and molecular analysis. The overall findings provided the first evidence about the lead structure for the potential development of quinoline-based NF-κB inhibitor.

## 2. Results and Discussion

**Scheme 1:**
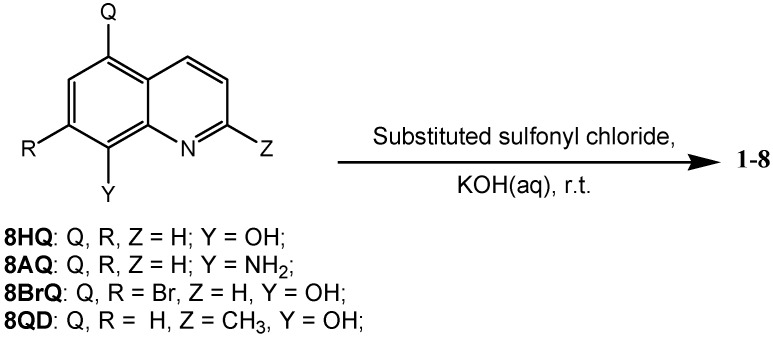
Synthesis of 8-substituted sulfonyl-containing quinolines.

We have previously reported a reaction for the preparation of 8-substituted quinoline ethers under basic condition in organic solvent with the help of phase transfer agent [14]. We now extended our reported protocol into a facile reaction in water for the substitution of 8-hydroxyquinoline (8HQ) and extended the protocol for various commercially available quinolines, such as 8-aminoquinoline (8AQ) and 2-methyl-8-hydroxyquinoline (8QD), to prepare the corresponding substituted quinolines for the study of substituent and linker (between quinoline core and sulfonyl group) effects on the biological activities towards cancer cells. Quinoline derivatives **1**–**8** were synthesized as simple and straightforward as shown in Scheme 1. In the presence of base and water as solvent, we diversified the quinoline core by substituting either the hydroxyl or amine group with a variety of substituted benzenesulfonyl chloride in the product yield of 23–96%. All the synthesized compounds were characterized by ^1^H-NMR and ^13^C-NMR, and the acquired spectra were shown in the supplementary information (Figures S1–S23). The molecular structure of our lead compound **5** was further confirmed by the X-ray crystallography; the obtained structure is shown in Figure S24 in the supplementary information.

Although the product yield of the substituted quinolines was not optimal, its potential for biomedical application was substantial, as the reactivity of compounds might be governed by both steric and electronic nature of the substituents on the aromatic ring of substrates. This method constituted a relatively “green” way to prepare substituted quinolines from commercially available 8-hydroxyquinoline in water. However, it deserved further optimization in the future to achieve higher yields.

**Figure 1:**
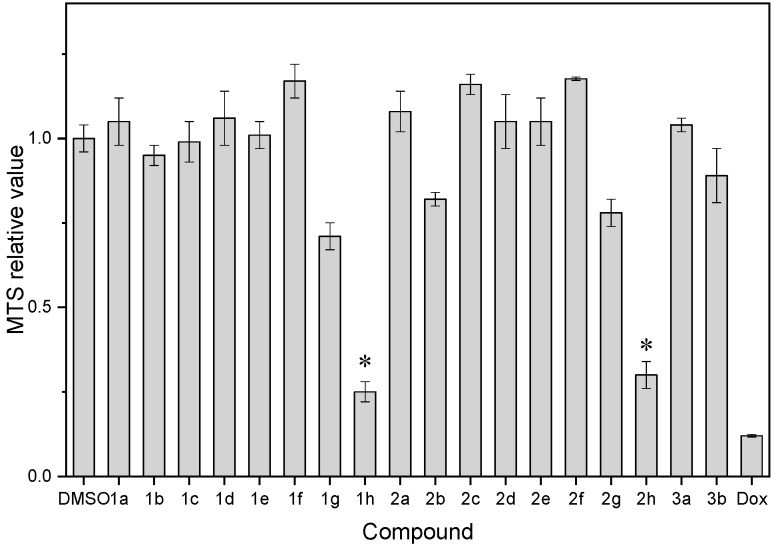
The MTS relative values of compounds **1** to **3** were compared with the vehicle control (DMSO) and the positive control (doxorubicin, Dox). *: compounds with MTS relative values <0.40.

Table 1 shows the biological activities of synthesized sulfonyl-containing quinolines (**1**–**3**) for the study of substituent effect on anticancer activity. These tested compounds were first evaluated using an in vitro MTS cytotoxicity assay [2] on hepatocellular carcinoma cells (Figure 1). First, molecules containing bromine atom showed stronger cytotoxicity on cancer cells than that containing trifluoromethyl group or unsubstituted. Among them, **1h** and **2h** exhibited the best anticancer activity against liver cancer cells (Hep3B), indicating the importance of the presence of bromine atom in the selected chromophore. Second, regarding the position of bromine atom on quinoline ring (**2b**–**2d**), the bromine atom at ortho-position of the aromatic ring was associated with better activity than those at *m*- or *p*-position. Third, the presence of an additional methyl group at 2 position (i.e., compound **2**) and changing the aromatic ring size (i.e., compound **1** vs. **3** and compound **2** vs. **3**) may not help to improve the anticancer activity on cancer cells for these types of compounds. Accordingly, the combined effect of the presence of bromine atom and bulky group at the 8-position attributed to the significant cell death observed in Hep3B cancer cells.

**Figure 2:**
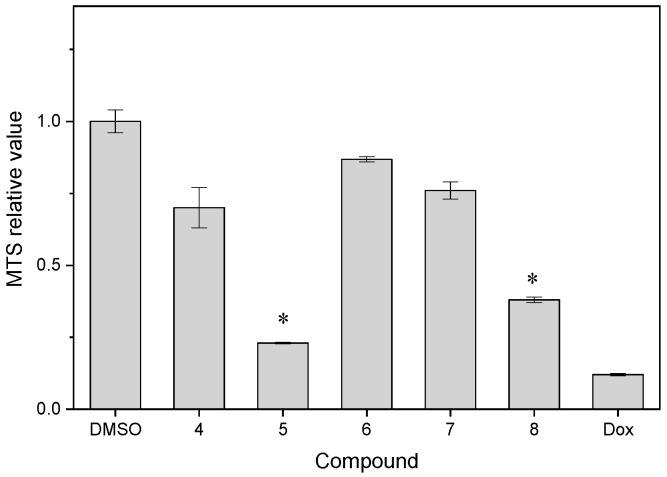
The MTS relative values of compounds **4** to **8** were compared with the vehicle control (DMSO) and the positive control (doxorubicin, Dox). * compounds with MTS relative values <0.40.

**Figure 3:**
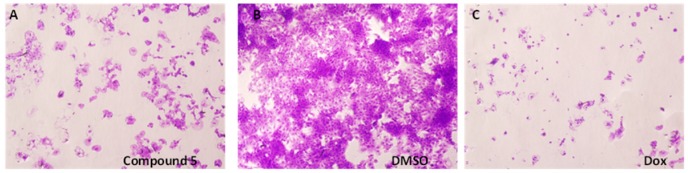
Morphological changes of (**A**) Hep3B cells after the 48 h treatment with compound **5** (at 1.8 μM), in comparison with (**B**) the vehicle control (DMSO) and (**C**) the positive control, doxorubicin (Dox, at 1.8 μM). Cancer cells showed shrinkage after the treatment of compound **5** and doxorubicin. The cells were stained by crystal violet. Each treatment was repeated in technical triplicates.

To further explore the structural-and-activity relationship of sulfonyl-containing quinolines, we synthesized some of the dimeric derivatives with different linker groups (**4**–**8**). Table 2 presents the bioactivity of the dimeric quinoline derivatives containing sulfonyl group, while the MTS relative values are shown in Figure 2. First, compounds containing two quinoline rings (**4**–**8**) generally exhibited a much stronger anticancer activity than that of one quinolinyl group **(1**–**3**). Second, the anticancer activity was likely linked to the effect of altering the substituents at 2-position, such as the methyl group and aldehyde group. Third, we further modified the quinoline ring with bromine atoms at the 5th and 7th positions with a pronounced anticancer activity result, indicating the presence of a bromine atom is of certain importance towards anticancer activity. Furthermore, regarding to the linker effect, the bioactivity of the compound with the oxygen being replaced to nitrogen at the linker part (i.e., compound **5**) was greatly enhanced. The relative MTS value was reduced to 0.230, which was comparable to that of the positive control (a standard chemotherapeutic drug, doxorubicin) with an MTS value of 0.120. Figure 3 displays the morphological changes of Hep3B cells under the exposure of the lead compound **5**. The shrinkage of cancer cells was present after the treatment of compound **5**. In addition, we examined the cell-type specificity of the anticancer activity of compound **5** by evaluating its anticancer activity against three human cancer cell lines, including human liver cancer (Hep3B), human esophageal cancer (SLMT-1), and human breast cancer (MCF-7) cells. The determined MTS_50_ values in these three cell lines ranged from 1.23 to 6.53 μM (Table 3), suggesting the anticancer effect of **5** was not limited to the types of cancer.

**Figure 4:**
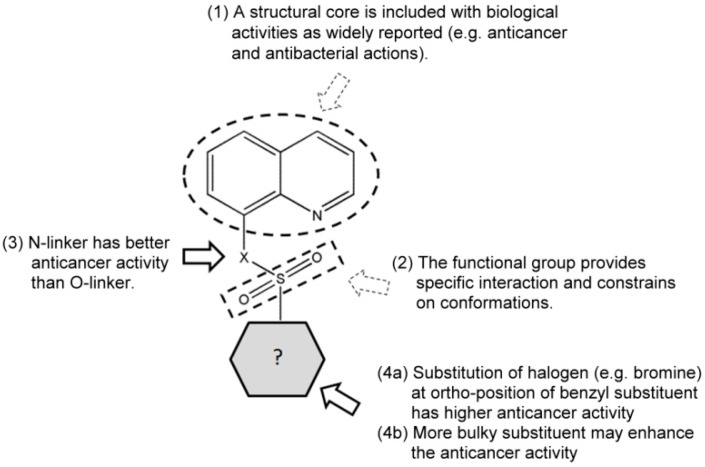
Structure-and-activity relationship of the designed and prepared sulfonyl-containing quinolines for studying both the linker and substituent effects on the anticancer activity.

The structure-and-anticancer activity relationship of our synthesized compounds was summarized in Figure 4. Taken together, our findings revealed some key structural requirements, including the presence of bulky groups (e.g., benzyl ring and quinolyl ring), sulfonamide group, and bromine atom, for the activity against Hep3B cancer cells.

After the lead compound **5** was identified, we further described the drug-like properties of this series of compounds with some known algorithms [19,21]. Log P estimates the lipophilicity of a chemical compound and drug score reflects the overall potential of a compound to be a drug. The calculated log P and drug scores are listed in Table 1 and Table 2. The data showed that compound **5** had the highest drug score among the test compounds, and had a log P value of 2.40 for drug permeation across a biological membrane. With the desired in vitro anticancer activity and drug-like properties, we employed the similarity ensemble approach [20] to identify the potential molecular targets of our lead compound **5** and other derivatives. The expectation value (*E*-value) determined from this approach indicates the chemical similarity between the biological targets and the ligands. According to the molecular docking results obtained from the online similarity ensemble approach search tool [20], the nuclear factor NF-κB p65 subunit was identified as the first molecular target in all the tested compounds, except for **1f,** which was at the second priority. The corresponding *E*-values are also listed in Table 1 and Table 2. These data reflected the class of tested sulfonyl-containing quinoline derivatives, all potentially participating in the NF-κB pathway. Interestingly, **5** has the highest priority towards the nuclear factor NF-κB p65 subunit with an *E*-value of 9.05× 10^−71^ and MaxTC of 0.75. Moreover, the derivatives containing two quinolinyl groups had smaller *E*-values than those derivatives containing one quinoline ring. This observation was in a good agreement with the trend obtained from our in vitro experimental results.

A study was also reported that *N*-(quinolin-8-yl)quinolin-8-sulfonamide (i.e.,compound**5** in this work), used as a ligand in a copper (II) complex, had a good ability in DNA binding and DNA cleavage [22]. In addition, Kim et al. reported that 2-amino-*N*-quinoline-8-yl benzenesulfonamide caused DNA fragmentation and led to subsequent apoptosis in Jurkat T cells [23]. With the support of these reported studies, we further investigated and revealed the potential binding between compound **5** and DNA and/or DNA binding factors which are crucial for anticancer activities.

**Figure 5:**
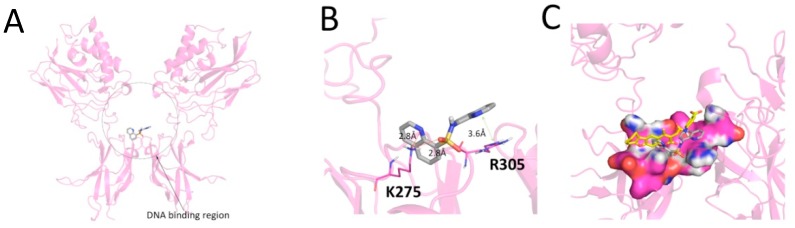
Results of docking simulation between compound **5** and NF-κB. Compound **5** was docked in NF-κB using the previously reported method. (**A**) Docking pose of inhibitor (gray carbon colored stick) in NF-κB (pink ribbon). (**B**) Details of the binding mode. Compound **5** established two H-bonds with lysine 275 and a face-to-face interaction with arginine 305. The distance and the interaction geometry between quinoline and arginine are consistent with literature data. (**C**) Comparison between the binding mode, previously reported, for psoralene compounds and the herein considered compound **5**.

**Figure 6:**
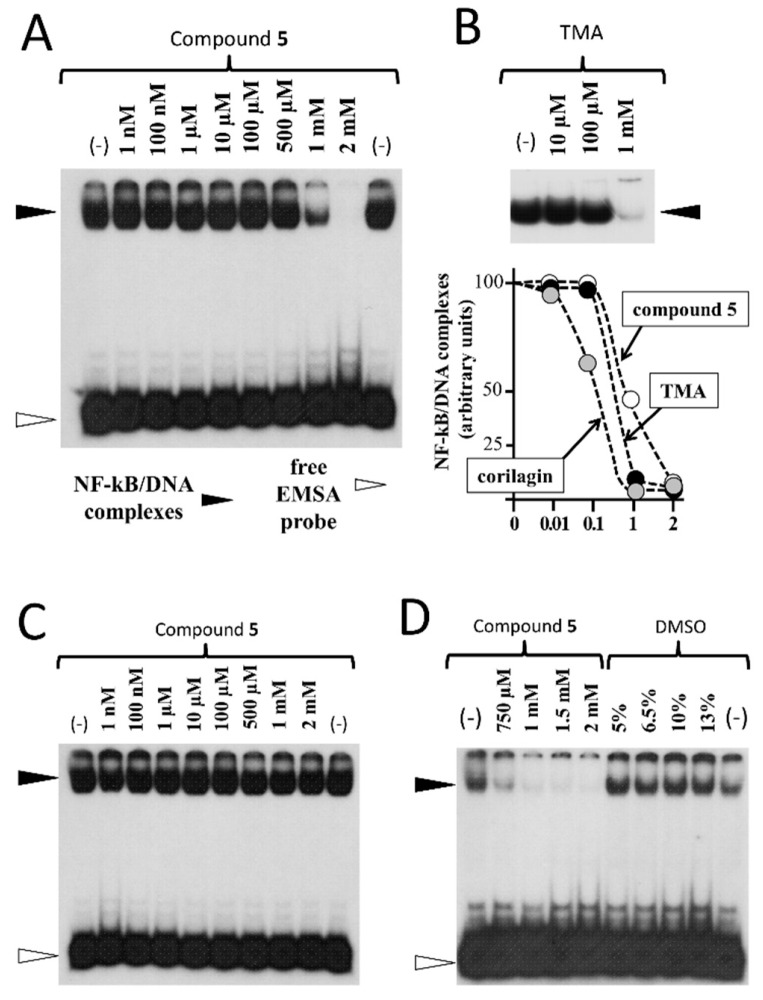
Effects of compound **5** on NF-κB/DNA interactions. (**A**) Compound **5** was pre-incubated for 20 min at the indicated concentrations with NF-κB p50 (Active Motif, cat.31101) and then a further 20 min incubation period was performed after adding the ^32^P-labelled NF-κB probe. Then the complexes were separated by polyacrylamide gel electrophoresis. (**B**) Representative data showing the effects of trimethylangelicin (TMA) on the generation of NF-κB/DNA complexes (upper part of the panel). In the lower part of the panel the comparison of the effects of compound **5**, TMA and corilagin is shown. (**C**) The same protocol described for panel A was followed, however, in this case, compound **5** was first incubated for 20 min with the ^32^P-labelled NF-κB probe, after which the NF-κB p50 protein was added. (**D**) The effects of compound **5** were not dependent from DMSO, added in the EMSA reactions at a concentration corresponding to those of compound **5** incubations.

Figure 5 shows the potential binding mode of compound **5** with the DNA binding region of NF-κB, as obtained through molecular docking simulation (AutoDock score = −5.4 Kcal/mol). The EMSA experiments [24,25] fully confirmed the data depicted in Figure 5. The data obtained (Figure 6) demonstrated that compound **5** inhibited the interactions between NF-κB and a ^32^P-labelled target oligonucleotide mimicking the NF-κB binding sites. The activity of compound **5** was similar to that of a known NF-κB inhibitor, trimethylangelicin (TMA); see the representative example shown in Figure 6B, upper part of the panel [25,26,27]. Moreover, when quantitative data obtained using compound **5**, TMA, and another validated NF-κB inhibitor (corilagin) [28] were compared, compound **5** was conclusively demonstrated to exert anti NF-κB activity leading to inhibition of the formation of NF-κB/DNA complexes with an efficiency similar to that of other NF-κB control inhibitors (Figure 6B, lower part of the panel). Finally, the inhibitory efficiency of compound **5** was in the range of the activity of other classes of NF-κB inhibitors studied by our group, including psoralene derivatives [25] and a set of low molecular weight compounds identified by virtual screening against p50 NF-κB (data not shown) [25,29]. In agreement with the docking results, the inhibition of NF-κB/DNA interactions was found only when compound **5** was pre-incubated with NF-kB, with ^32^P-labelled target NF-κB oligonucleotide added after (Figure 6A,D). On the contrary, no inhibition was obtained when the compound **5** was pre-incubated with the ^32^P-labelled EMSA probe and then NF-κB was added (Figure 6C), supporting the concept of a direct binding of compound **5** to NF-κB and not to DNA. This experiment, together with the docking data, suggested that the possible mechanism of action of compound **5** likely involved NF-κB binding and regulation.

Heterocycles, such as isoliensinine and coumarin analogs, showed inhibition of NF-κB activation. The coumarin analog reported by Neelgundmath et al. [30] possessed an ability to induce the apoptosis in HepG2 cells by suppressing the DNA binding ability of NF-κB, while a decrease in NF-κB activity and phosphorylation of NF-κB p65 subunit were observed in isoliensinine-treated HepG2 cells [31]. Additionally, a simple quinoline derivative, 8-hydroxyquinoline, was reported to inhibit the NF-κB activation in Raw 264.7 cells [32].

Quinoline is a remarkable class of compounds, found in both natural and synthetic sources, with a broad spectrum of biological activities [33]. We previously reported a series of 8-substituted quinoline ethers possessing anti-*Aspergillus niger* activity [14], and more recently, we discovered a hexahydrofuro[3,2-c]quinoline showed anti-breast cancer activity, as well as its enhanced effect on the fungistatic activity of micronazole [34]. Simeprevir, an orally-administered quinoline-based specific protease inhibitor, was approved by the Food and Drug Administration (FDA) for the clinical treatment of chronic hepatitis C virus infection [35,36,37]. This molecule contains aquinoline ring and a small sulfonamide group. Previous studies also reported 2-sulfolmethyl quinolines showing anti-hepatitis B virus activity [38] and antiproliferative activity [39] against HepG2 cancer cells in vitro.

Sulfonamides are well-known for their medicinal values and coordination properties that are able to bind to Zinc [40] or formed as metal complex to induce DNA damages by photoradiation [22]. However, studies on the anticancer effect of *O*-sulfonyl-containing quinolones are still sparse.

The functionality of sulfonyl moiety is presumably advantageous to a chemical structure of potential drug candidates, because of the availability of hydrogen bonding and the constraint on the side chains, which allow a specific conformation of molecules. These two important features could lead to stronger interaction at the active site of the biological targets. Herein, taking the promising anticancer effect of NF-κB inhibitors, we aimed to design and synthesize a series of sulfonyl-containing quinolines with rationale for the evaluation of substituent and linker effects on their in vitro anticancer activity. All synthesized compounds were screened for their in vitro anticancer activity in Hep3B hepatocellular carcinoma cells. Subsequently, the lead compound was also tested on esophageal carcinoma and breast cancer cells. Most importantly, the mechanism of anticancer effect was studied by the bioinformatics approach with a molecular docking analysis (similarity ensemble approach, SEA) followed by the associated molecular studies. The overall findings of the present work paved the new path for developing the sulfonyl-containing quinolines as the leads for future anticancer drug development which combines the chemical synthesis, genomic, and bioinformatics-based strategies.

The transcription factor NF-κB is a potential therapeutic target. Regulation of NF-κB may result in a targeted therapy and control of the chemoresistance in cancer cells [41]. This target is a transcription factor and plays a decisive role in several biological processes, involving cell cycle regulation [42], cell differentiation, and apoptosis [43,44]. More importantly, it was strongly suggested that targeting NF-κB could be an effective direction for anticancer treatment, as it suppresses cancer cell migration and epithelia-mesenchymal transition [45,46]. Previous studies showed that targeting the NF-κB had anticancer effects in breast cancer [47], colon cancer [48], and esophageal cancer cells [49]. Zuo et al. [50] also demonstrated the modulation of NF-κB in the regulation of human telomerase reverse transcriptase (hTERT) gene transcription, which was highly correlated to the pathogenesis of hepatocellular carcinoma.

The results presented support the concept that compound **5** deserves further studies to better characterize its biological effects on one side, and its mechanism of action on the other. These further efforts include (but are not limited to) the analysis of the activity on other transcription factors (in order to further verify the specificity of the treatment), the analysis of transcriptome (in order to verify the activity on NF-κB regulated genes, including those involved in cell migration and epithelia-mesenchymal transition process) and chromatin immunoprecipitation assays (in order to map the lack of binding of NF-κB to gene promoters containing NF-κB binding sites).

In conclusion, the results presented in this study identified a lead compound (compound **5**) among a series of 8-substituted sulfonyl-containing quinolines which potentially targets NF-κB signaling to induce cell death in hepatocellular cancer. It also provided the first evidence for the anticancer effect of quinoline-type compound **5** with the binding to NF-κB based on the bioinformatics and proven by molecular analysis. Further full-scale investigations will involve the anticancer studies on the suppression on cancer cell migration, epithelia-mesenchymal transition and the use of chromatin immunoprecipitation (ChIP) assay for probing protein-DNA interactions in HCC and other cancer types to fully elucidate the underlying anticancer actions for the cell death induced by our lead compound **5** as well as further development of effective anticancer strategies.

## 3. Materials and Methods

### 3.1. Chemistry

#### 3.1.1. Materials

All reagents and chemicals were purchased from commercial sources. The synthesized compounds were characterized by melting point, mass spectrometry, ^1^H-, and ^13^C-NMR. ^1^H- and ^13^C-NMR spectra were recorded on a Brucker DPX 400 spectrometer (Bruker, Switzerland) in CDCl_3_ unless otherwise specified.

#### 3.1.2. General Procedure for the Synthesis of **1**–**4**,**6**

To get a solution of 8-hydroxyquinoline or 8-hydroxyquinaldine (0.69 mmol, 1.1 equiv) in water (2 mL), substituted benzenesulfonyl chloride (0.62 mmol, 1.0 equiv) and potassium hydroxide (0.69 mmol, 1.1 equiv) were added and stirred at room temperature. The reaction progress was monitored by thin layer chromatography. After the reaction completed, the aqueous phase was extracted with dichloromethane and dried over anhydrous sodium sulfate. Solvent was removed by rotary evaporator (Heidolph, Schwabach, Germany) and the crude product was purified by silica gel column chromatography.

#### 3.1.3. General Procedure for the Synthesis of **5**

A solution of 8-aminoquinoline (0.62 mmol, 1.0 equiv), 8-quinolinesulfonyl chloride (0.62 mmol, 1.0 equiv), and triethylamine (0.62 mmol, 1.0 equiv) in acetonitrile was stirred at room temperature. The reaction progress was monitored by thin layer chromatography. When the reaction completed, solvent was removed under reduced pressure. The reaction mixture was extracted with dichloromethane and washed with water, dried over anhydrous sodium sulfate. Solvent was removed by rotary evaporator (Heidolph, Schwabach, Germany) and the crude product was purified by silica gel column chromatography.

#### 3.1.4. General Procedure for the Synthesis of **8**

Compound **8** was prepared from the reactant, 5,7-dibromo-8-hydroxyquinoline, using the method previously reported by our group [14]. The preparation followed the protocol stated in the study, unless otherwise specified.

#### 3.1.5. General Procedure for the Synthesis of **7**

Compound **7** was prepared by the oxidation of compound **6** using selenium dioxide under reflux in 1,4-dioxane, where the method was previously reported by our group [51].The preparation followed the protocol stated in the study, unless otherwise specified.

The characterization of the synthesized compounds is described in the supplementary information.

### 3.2. Biological Analysis

#### 3.2.1. Cell Lines and Cell Culture

Human hepatocellular carcinoma Hep3B, human esophageal squamous cell carcinoma SLMT-1 [52], and human breast carcinoma MCF-7 cell line were used for the preliminary evaluation of the possible anticancer activity of the synthesized compounds. Hep3B, and MCF-7 cell lines were cultured in 90% of DMEM (Dulbecco’s Modified Eagle Medium) culture medium supplemented with 10% fetal bovine serum and 1% penicillin antibiotics while SLMT-1 cell line was cultured in 90% of MEM (Minimum Essential Medium) Alpha culture medium supplemented with 10% fetal bovine serum and 1% penicillin antibiotics. The cells were incubated at 37°C, 5% CO_2_.

#### 3.2.2. In Vitro MTS Cytotoxicity Assay

The anticancer activity of synthesized compounds was evaluated against three cancer cell lines, namely Hep3B, SLMT-1, and MCF-7, by standard MTS assay as we reported before [53]. Cells were seeded in 96 well microtiter plates with a cell density of 5000 for 24 h for anticancer activity screening. Doxorubicin (Dox) and dimethylsulfoxide (DMSO, molecular biology grade) were used as positive control and a vehicle control, respectively. Synthesized compounds were added at the desired concentration and incubated for further 48 h. The relative cell viability in each well was determined by using MTS assay. Each treatment point was repeated in three technical and independent replicates.

#### 3.2.3. Molecular Docking Analyses

Computational studies were conducted on a 4 CPU (Intel Core2 Quad CPU Q9550, 2.83 GHz, Ubuntu, London, UK) ACPI x46 Linux workstation with Ubuntu 12.04 operating system (Ubuntu, London, UK). The NF-κB structure was derived from the Protein Data Bank (PDB code: 1NFK, p50) and handled with Chimera 5.3.1 software (RBVI, Resource for Biocomputing, Visualization, and Informatics, University of California, CA,USA, www.cgl.ucsf.edu/chimera/) [54]. The structure of compound **5** was prepared with MarvinSketch 5.5 software (Marvin, version 5.5.0.1, Program B; ChemAxon: Budapest, Hungary; www.chemaxon.com/products) and was minimized and protonated (pH = 7.4) with OpenBabel 2.2.3 software (Version 2.2.3, http://openbabel.org) [55], using the MMFF94s force field. Docking studies were performed with AutoDock 4.2 software (http://autodock.scripps.edu/) provided with AutoDock Tools 1.5.4 graphical interface [56]. According to the previous study [25], docking simulations were performed in a docking box characterized by 0.375 Å spacing and by 54 × 54 × 54 points dimensions. The box was centered at x = −11.11, y = 18.23, z = 15.33. Docking pose was obtained through Lamarckian genetic algorithm searching engine, carrying out 50 runs with a maximum of 2,500,000 energy evaluations. Cluster analysis was performed on obtained results, with a root-mean square tolerance of 2.0 Å, finally selecting the lowest energy pose.

#### 3.2.4. Electrophoretic Mobility Shift Assay (EMSA)

Electrophoretic mobility shift assays were performed by using as target DNA double-stranded ^32^P-labeled oligonucleotides mimicking an NF-κB binding site. Binding reactions were set up as described elsewhere in binding buffer (5% glycerol, 20 mM Tris-HCl pH 7.5, 1 mM DTT, 0.01% TRITON X-100, 50 mM KCl, 0.2 mM EDTA, 0.05 µg/µL dIdC and 1 mM MgCl_2_) and labeled oligonucleotides were incubated in the presence of compound **5** at different concentrations. The effects of DMSO on NF-κB/DNA interactions were analyzed at relevant concentrations, demonstrating no inhibitory effects. After 20 min of binding of compound **5** (resuspended in DMSO) at room temperature to NF-κB-p50 (Active Motif, cat.31101), incubation with the ^32^P-labeled NF-κB oligonucleotides was performed in a total final volume of 20 μL for additional 20 min, at room temperature. Then, the samples were electrophoresed at constant voltage (200V for 30 min) through low ionic strength buffer (0.25×)Tris/borate/EDTA buffer (22.5 mM Tris, 22.5 mM boric acid, 0.5 mM EDTA, pH 8) on 6% polyacrylamide gels until tracking dye (bromophenol blue) reached the end of a 16 cm slab. Gels were dried and exposed for autoradiography with intensifying screens at −80 °C. In these experiments, DNA/protein complexes migrate through the gel with slower efficiency. The nucleotide sequences of double-stranded target DNA utilized in these experiments were 5′-ATC GGG GGA AAT CCC AGA AC-3′ (sense strand, NF-κB). The synthetic oligonucleotides used in this study were purchased from IDT (Integrated DNA Technologies, Leuven, Belgium).Control NF-κB inhibitors were trimethylangelicin (TMA) and β-1-*O*-galloyl-3,6-(*R*)-hexahydroxydiphenoyl-*d*-glucose (corilagin). Synthesis of TMA has been reported elsewhere [25,26,27]; corilagin was obtained from the China National Institute for the Control of Pharmaceutical and Biological Products. For quantitative analyses, the autoradiographs were scanned through a Gel-Doc Instrument (Biorad, Hercules, CA, USA) using the QuantityOne software (Version 4, Biorad, Hercules, CA, USA).

## Figures and Tables

**Table 1:**
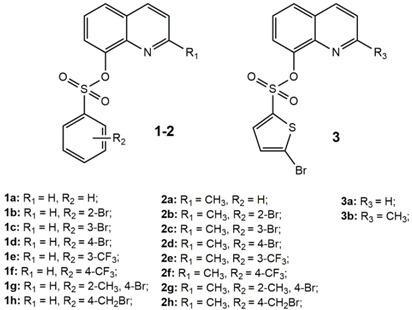
Bioactivity of the tested compounds (**1–3**) against liver cancer cell line Hep3B and the related docking results.

Compound	MTS Relative Values ^a^ (Mean ± SD)	Log P ^b^	Drug Score ^c^	Nuclear Factor NF-κB p65 Subunit: *E*-Value ^d^
1a	1.05 ± 0.07	2.76	0.15	2.59 × 10^−20^
1b	0.95 ± 0.03	4.10	0.37	4.01 × 10^−21^
1c	0.99 ± 0.06	3.17	0.36	1.12 × 10^−16^
1d	1.06 ± 0.08	3.19	0.38	2.38 × 10^−12^
1e	1.01 ± 0.04	3.50	0.22	7.92 × 10^−13^
1f	1.17 ± 0.05	3.50	0.36	7.92 × 10^−13^
1g	0.71 ± 0.04	3.21	0.21	5.39 × 10^−17^
1h	0.25 ± 0.03	3.27	0.10	8.61 × 10^−7^
2a	1.08 ± 0.06	3.18	0.15	6.20 × 10^−17^
2b	0.82 ± 0.02	3.59	0.33	9.52 × 10^−18^
2c	1.16 ± 0.03	3.60	0.33	1.13 × 10^−20^
2d	1.05 ± 0.08	3.61	0.34	1.01 × 10^−15^
2e	1.05 ± 0.07	3.91	0.20	2.74 × 10^−21^
2f	1.18 ± 0.005	3.90	0.33	1.46 × 10^−18^
2g	0.78 ± 0.04	3.63	0.19	4.07 × 10^−16^
2h	0.30 ± 0.04	3.66	0.09	7.92 × 10^−13^
3a	1.04 ± 0.02	3.12	0.22	1.08 × 10^−17^
3b	0.89 ± 0.08	3.49	0.20	7.19 × 10^−15^

^a^ Cytotoxicity of all compounds **1** to **3** was determined against Hep3B cells using the cell proliferation assay (MTS assay). Cells were treated with the tested compounds at 15 μM for 48 h. Doxorubicin (Dox) was used as positive control while dimethyl sulfoxide (DMSO) was used as vehicle control. Each treatment was performed in three technical and independent replicates, and the average reading to the vehicle control was calculated as the relative MTS value. MTS relative values of DMSO and positive control (Dox) were determined as 1.00 ± 0.04 and 0.120 ± 0.004, respectively. ^b^ Estimated by the ALOGPS 2.1 algorithm [19]. ^c^ Estimated by the Osiris Property Explorer (https://www.organic-chemistry.org/prog/peo/).^d^ Estimated by the similarity ensemble approach (SEA) [20].

**Table 2:**
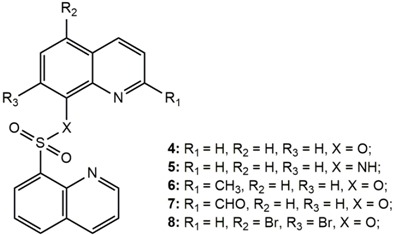
Bioactivity of the tested compounds (**4**–**8**) against Hep3B cell lines and the related docking results.

Compound	MTS Relative Values ^a^ (Mean ± SD)	Log P ^b^	Drug Score ^c^	Nuclear Factor NF-κB p65 Subunit: *E*-Value ^d^
4	0.70 ± 0.07	2.60	0.45	4.63 × 10^−24^
5	0.230 ± 0.003	2.40	0.60	9.05 × 10^−71^
6	0.868 ± 0.009	3.04	0.41	4.45 × 10^−20^
7	0.76 ± 0.03	2.96	0.37	1.27 × 10^−36^
8	0.38 ± 0.01	3.66	0.13	5.18 × 10^−9^

^a^ Cytotoxicity of all compounds were determined against Hep3B cells using the cell proliferation assay (MTS assay) as described in the legend of Table 1. Each treatment was performed in three technical and independent replicates. MTS relative values of DMSO and positive control (Dox) were determined as 1.00 ± 0.04 and 0.120 ± 0.004, respectively. ^b^ Estimated by the ALOGPS 2.1 algorithm [19]. ^c^ Estimated by the Osiris Property Explorer (https://www.organic-chemistry.org/prog/peo/). ^d^ Estimated by the similarity ensemble approach (SEA) [20].

**Table 3:** Cytotoxicity of compound **5** against three human carcinoma cell lines ^†^.

Cell Line	MTS_50_ Values (μM) (Mean ± SD)	
	5	Dox
Hep3B	2.91 ± 0.09	0.27 ± 0.02
SLMT-1	6.53 ± 0.06	0.41 ± 0.03
MCF-7	1.23 ± 0.03	0.28 ± 0.04

^†^ MTS assay was used to evaluate the cytotoxicity of compound **5** on cancer cells. MTS_50_ is defined as the concentration of the test agent required to reduce the MTS activity by 50% when compared with the vehicle treated control. Each treatment was performed in three technical and independent replicates and the corresponding MTS_50_ values of compound **5** and doxorubicin (Dox) were obtained for the three human carcinoma cell lines, and treated for 48 h.

## Supplementary Materials

The supplementary materials are available.

## References

[1] Jemal A., Siegel R., Xu J.Q., Ward E. (2010). Cancer Statistics, 2010. CA. Cancer J. Clin..

[2] Chan D., Zhou Y., Chui C.H., Lam K.H., Law S., Chan A.S.C., Li X., Lam A.K.Y., Tang J.C.O. (2018). Expression of insulin-like growth factor binding protein-5 (IGFBP5) reverses cisplatin-resistance in esophageal carcinoma. Cells.

[3] Mizuno M., Tan R.X., Zhen P., Min Z.D., Iinuma M., Tanaka T. (1990). Two steroidal alkaloid glycosides from Veratrum taliense. Phytochemistry.

[4] Ge H.M., Shen Y., Zhu C.H., Tan S.H., Ding H., Song Y.C., Tan R.X. (2008). Penicidones A–C, three cytotoxic alkaloidal metabolites of an endophytic *Penicillium* sp.. Phytochemistry.

[5] Worthen D.R., Ghosheh O.A., Crooks P.A. (1998). The in vitro anti-tumor activity of some crude and purified components of blackseed, *Nigella sativa* L.. Anticancer Res..

[6] Fattorusso E., Taglialatela-Scafati O. (2008). Modern Alkaloids: Structure, Isolation, Synthesis and Biology.

[7] Kumar S., Bawa S., Gupta H. (2009). Biological activities of quinoline derivatives. Mini-Rev. Med. Chem..

[8] Hu Z., Zhou Z., Hu Y., Wu J., Li Y., Huang W. (2015). HZ08 reverse P-glycoprotein mediated multidrug resistance in vitro and in vivo. PLoS ONE.

[9] Hesse M. (2002). Alkaloids: Nature’s Curse or Blessing?.

[10] Kouznetsov V.V.M., Leonor Y., Vargas M., Melendez Gomez C.M. (2005). Recent progress in the synthesis of quinolines. Curr. Org. Chem..

[11] Chung P.-Y., Bian Z.-X., Pun H.-Y., Chan D., Chan A.S.-C., Chui C.-H., Tang J.C.-O., Lam K.-H. (2015). Recent advances in research of natural and synthetic bioactive quinolines. Future Med. Chem..

[12] Afzal O., Kumar S., Haider M.R., Ali M.R., Kumar R., Jaggi M., Bawa S. (2015). A review on anticancer potential of bioactive heterocycle quinoline. Eur. J. Med. Chem..

[13] Lam K.H., Lee K.K., Gambari R., Kok S.H., Kok T.W., Chan A.S., Bian Z.X., Wong W.Y., Wong R.S., Lau F.Y. (2014). Anti-tumour and pharmacokinetics study of 2-Formyl-8-hydroxy-quinolinium chloride as galipea longiflora alkaloid analogue. Phytomedicine.

[14] Chung P.-Y., Gambari R., Chen Y.-X., Cheng C.-H., Bian Z.-X., Chan A.S.-C., Tang J.C.-O., Leung P.H.-M., Chui C.-H., Lam K.-H. (2014). Development of 8-benzyloxy-substituted quinoline ethers and evaluation of their antimicrobial activities. Med. Chem. Res..

[15] Ratheesh M., Sindhu G., Helen A. (2013). Anti-inflammatory effect of quinoline alkaloid skimmianine isolated from *Ruta Graveolens* L.. Inflamm. Res..

[16] Katanoda K., Matsuda T. (2014). Five-year relative survival rate of liver cancer in the USA, Europe and Japan. Jpn. J. Clin. Oncol..

[17] Kudo M. (2012). Targeted therapy for liver cancer: Updated review in 2012. Curr. Cancer Drug Targets.

[18] Lin P.P., Pang Q.S., Wang P., Lv X.Y., Liu L.F., Li A.K. (2018). The targeted regulation of Gli1 by miR-361 to inhibit epithelia-mesenchymal transition and invasion of esophageal carcinoma cells. Cancer Biomark..

[19] Tetko I.V., Gasteiger J., Todeschini R., Mauri A., Livingstone D., Ertl P., Palyulin V.A., Radchenko E.V., Zefirov N.S., Makarenko A.S. (2005). Virtual computational chemistry laboratory—Design and description. J. Comput.-Aided Mol. Des..

[20] Keiser M.J., Roth B.L., Armbruster B.N., Ernsberger P., Irwin J.J., Shoichet B.K. (2007). Relating protein pharmacology by ligand chemistry. Nat. Biotechnol..

[21] Fernandes I., Vale N., de Freitas V., Moreira R., Mateus N., Gomes P. (2009). Anti-tumoral activity of imidazoquines, a new class of antimalarials derived from primaquine. Bioorg. Med. Chem. Lett..

[22] Pascual-Álvarez A., Topala T., Estevan F., Sanz F., Alzuet-Piña G. (2016). Photoinduced and self-activated nuclease activity of copper(II) complexes with *N*-(Quinolin-8-yl)quin-olin-8-sulfonamide—DNA and bovine serum albumin binding. Eur. J. Inorg. Chem..

[23] Kim Y.-H., Shin K.-J., Lee T.G., Kim E., Lee M.-S., Ryu S.H., Suh P.-G. (2005). G2 arrest and apoptosis by 2-amino-*N*-quinoline-8-yl-benzenesulfonamide (QBS), a novel cytotoxic compound. Biochem. Pharmacol..

[24] Finotti A., Borgatti M., Bezzerri V., Nicolis E., Lampronti I., Dechecchi M., Mancini I., Cabrini G., Saviano M., Avitabile C. (2012). Effects of decoy molecules targeting NF-κB transcription factors in cystic fibrosis IB3–1 cells: Recruitment of NF-κB to the IL-8 gene promoter and transcription of the IL-8 gene. Artif. DNA PNA XNA.

[25] Marzaro G., Guiotto A., Borgatti M., Finotti A., Gambari R., Breveglieri G., Chilin A. (2013). Psoralen derivatives as inhibitors of NF-κB/DNA interaction: Synthesis, molecular modeling, 3D-QSAR, and biological evaluation. J. Med. Chem..

[26] Marzaro G., Lampronti I., D’Aversa E., Sacchetti G., Miolo G., Vaccarin C., Cabrini G., Dechecchi M.C., Gambari R., Chilin A. (2018). Design, synthesis and biological evaluation of novel trimethylangelicin analogues targeting nuclear factor κB (NF-κB). Eur. J. Med. Chem..

[27] Lampronti I., Manzione M.G., Sacchetti G., Ferrari D., Spisani S., Bezzerri V., Finotti A., Borgatti M., Dechecchi M.C., Miolo G. (2017). Differential effects of angelicin analogues on NF-κB activity and IL-8 gene expression in cystic fibrosis IB3-1 cells. Mediat. Inflamm..

[28] Gambari R., Borgatti M., Lampronti I., Fabbri E., Brognara E., Bianchi N., Piccagli L., Yuen C.W.M., Kan C.W., Hau D.K.P. (2012). Corilagin is a potent inhibitor of NF-κB activity and downregulates TNF-alpha induced expression of IL-8 gene in cystic fibrosis IB3-1 cells. Int. Immunopharmacol..

[29] Piccagli L., Fabbri E., Borgatti M., Bianchi N., Bezzerri V., Mancini I., Nicolis E., Dechecchi C.M., Lampronti I., Cabrini G. (2009). Virtual screening against p50 NF-κB transcription factor for the identification of inhibitors of the NF-κB-DNA interaction and expression of NF-κB upregulated genes. ChemMedChem.

[30] Neelgundmath M., Dinesh K.R., Mohan C.D., Li F., Dai X., Siveen K.S., Paricharak S., Mason D.J., Fuchs J.E., Sethi G. (2015). Novel synthetic coumarins that targets NF-κB in hepatocellular carcinoma. Bioorg. Med. Chem. Lett..

[31] Shu G.W., Yue L., Zhao W.H., Xu C., Yang J., Wang S.B., Yang X.Z. (2015). Isoliensinine, a bioactive alkaloid derived from embryos of nelumbo nucifera, induces hepatocellular carcinoma cell apoptosis through suppression of NF-κB signaling. J. Agric. Food. Chem..

[32] Kim Y.-H., Woo K.J., Lim J.H., Kim S., Lee T.J., Jung E.M., Lee J.-M., Park J.-W., Kwon T.K. (2005). 8-Hydroxyquinoline inhibits iNOS expression and nitric oxide production by down-regulating LPS-induced activity of NF-κB and C/EBPβ in Raw 264.7 cells. Biochem. Biophys. Res. Commun..

[33] Musiol R. (2017). An overview of quinoline as a privileged scaffold in cancer drug discovery. Expert Opin. Drug Discov..

[34] Chung P.Y., Tang J., Cheng C.H., Bian Z.X., Wong W.Y., Lam K.H., Chui C.H. (2016). Synthesis of hexahydrofuro [3,2-*c*]quinoline, a martinelline type analogue and investigation of its biological activity. SpringerPlus.

[35] Chopp S., Vanderwall R., Hult A., Klepser M. (2015). Simeprevir and sofosbuvir for treatment of hepatitis C infection. Am. J. Health Syst. Pharm..

[36] Sanford M. (2015). Simeprevir: A review of its use in patients with chronic hepatitis C virus infection. Drugs.

[37] Rosenquist A., Samuelsson B., Johansson P.O., Cummings M.D., Lenz O., Raboisson P., Simmen K., Vendeville S., de Kock H., Nilsson M. (2014). Discovery and development of simeprevir (TMC435), a HCV NS3/4A protease inhibitor. J. Med. Chem..

[38] Liu Y., Zhao Y., Zhai X., Liu X., Sun L., Ren Y., Gong P. (2008). Synthesis and anti-HBV activities evaluation of new ethyl 8-Imidazolylmethyl-7-hydroxyquinoline-3-carboxylate derivatives in vitro. Arch. Pharm..

[39] Jiang N., Zhai X., Li T., Liu D., Zhang T., Wang B., Gong P. (2012). Design, synthesis and antiproliferative activity of novel 2-substituted-4-amino-6-halogenquinolines. Molecules.

[40] Rouffet M., de Oliveira C.A.F., Udi Y., Agrawal A., Sagi I., McCammon J.A., Cohen S.M. (2010). From sensors to silencers: Quinoline-and benzimidazole-sulfonamides as inhibitors for zinc proteases. J. Am. Chem. Soc..

[41] Arepalli S.K., Choi M., Jung J.K., Lee H. (2015). Novel NF-κB inhibitors: A patent review (2011–2014). Expert Opin. Ther. Patents.

[42] Chen F., Castranova V., Shi X. (2001). New insights into the role of fuclear factor-κB in cell growth regulation. Am. J. Pathol..

[43] Guttridge D.C., Albanese C., Reuther J.Y., Pestell R.G., Baldwin A.S. (1999). NF-κB controls cell growth and differentiation through transcriptional regulation of cyclin D1. Mol. Cell. Biol..

[44] Vasudevan K.M., Gurumurthy S., Rangnekar V.M. (2004). Suppression of PTEN expression by NF-κB prevents apoptosis. Mol. Cell. Biol..

[45] Wang J.J., Tian L.L., Khan M.N., Zhang L., Chen Q., Zhao Y., Yan Q., Fu L., Liu J.W. (2018). Ginsenoside Rg3 sensitizes hypoxic lung cancer cells to cisplatin via blocking of NF-κB mediated epithelial-mesenchymal transition and sternness. Cancer Lett..

[46] Lee H., Pyo M.J., Bae S.K., Heo Y., Choudhary I., Hwang D., Yang H., Kim J.H., Chae J., Han C.H. (2018). Nemopilema nomurai jellyfish venom exerts an anti-metastatic effect by inhibiting Smad- and NF-κB-mediated epithelial-mesenchymal transition in HepG2 cells. Sci. Rep..

[47] Arora S., Kothandapani A., Tillison K., Kalman-Maltese V., Patrick S.M. (2010). Downregulation of XPF–ERCC1 enhances cisplatin efficacy in cancer cells. DNA Repair.

[48] Zhang L.L., Mu G.G., Ding Q.S., Li Y.X., Shi Y.B., Dai J.F., Yu H.G. (2015). Phosphatase and tensin homolog (PTEN) represses colon cancer progression through inhibiting paxillin transcription via PI3K/AKT/NF-κB Pathway. J. Biol. Chem..

[49] Lin C., Song L., Gong H., Liu A., Lin X., Wu J., Li M., Li J. (2013). Nkx2-8 downregulation promotes angiogenesis and activates NF-κB in esophageal cancer. Cancer Res..

[50] Zuo Q.-P., Liu S.-K., Li Z.-J., Li B., Zhou Y.-L., Guo R., Huang L.-H. (2011). NF-κB p65 modulates the telomerase reverse transcriptase in the HepG2 hepatoma cell line. Eur. J. Pharmacol..

[51] Chan S.H., Chui C.H., Chan S.W., Kok S.H.L., Chan D., Tsoi M.Y.T., Leung P.H.M., Lam A.K.Y., Chan A.S.C., Lam K.H. (2013). Synthesis of 8-Hydroxyquinoline derivatives as novel antitumor agents. ACS Med. Chem. Lett..

[52] Tang J.C.O., Wan T.S.K., Wong N., Pang E., Lam K.Y., Law S.Y., Chow L.M.C., Ma E.S.K., Chan L.C., Wong J. (2001). Establishment and characterization of a new xenograft-derived human esophageal squamous cell carcinoma cell line SLMT-1 of Chinese origin. Cancer Genet. Cytogenet..

[53] Pun I.H.Y., Chan D., Chan S.H., Chung P.Y., Zhou Y.Y., Law S., Lam A.K.Y., Chui C.H., Chan A.S.C., Lam K.H. (2017). Anti-cancer effects of a novel quinoline derivative 83b1 on human esophageal squamous cell carcinoma through down-regulation of COX-2 mRNA and PGE(2). Cancer Res. Treat..

[54] Pettersen E.F., Goddard T.D., Huang C.C., Couch G.S., Greenblatt D.M., Meng E.C., Ferrin T.E. (2004). UCSF Chimera—A visualization system for exploratory research and analysis. J. Comput. Chem..

[55] O’Boyle N., Banck M., James C., Morley C., Vandermeersch T., Hutchison G. (2011). Open babel: An open chemical toolbox. J. Cheminformatics.

[56] Morris G.M., Huey R., Lindstrom W., Sanner M.F., Belew R.K., Goodsell D.S., Olson A.J. (2009). AutoDock4 and AutoDockTools4: Automated docking with selective receptor flexibility. J. Comput. Chem..

