# Supplementary material for: Targeting DNA Binding for NF-κB as an Anticancer Approach in Hepatocellular Carcinoma
Source: Cells. 2018 Oct 22;7(10):177. doi: 10.3390/cells7100177 (PMC6209864; doi:10.3390/cells7100177)
Supplement: Supplementary file 1 [file cells-07-00177-s001.pdf]

Supplementary information

Po-yee CHUNG, *et al*.

Targeting DNA binding for NF-κB as an anticancer approach in hepatocellular carcinoma

Content

	Page
**Characterization of the synthesized compounds**	1-5
^{1}H NMR and ^{13}C NMR	
**Figure S1**. ^{1}H (400 MHz) and ^{13}C (100 MHz) NMR spectra of **1a** in $CDCl_3$.	6
**Figure S2**. ^{1}H (400 MHz) and ^{13}C (100 MHz) NMR spectra of **1b** in $CDCl_3$.	7
**Figure S3**. ^{1}H (400 MHz) and ^{13}C (100 MHz) NMR spectra of **1c** in $CDCl_3$.	8
**Figure S4**. ^{1}H (400 MHz) and ^{13}C (100 MHz) NMR spectra of **1d** in $CDCl_3$.	9
**Figure S5**. ^{1}H (400 MHz) and ^{13}C (100 MHz) NMR spectra of **1e** in $CDCl_3$.	10
**Figure S6**. ^{1}H (400 MHz) and ^{13}C (100 MHz) NMR spectra of **1f** in $CDCl_3$.	11
**Figure S7**. ^{1}H (400 MHz) and ^{13}C (100 MHz) NMR spectra of **1g** in $CDCl_3$.	12
**Figure S8**. ^{1}H (400 MHz) and ^{13}C (100 MHz) NMR spectra of **1h** in $CDCl_3$.	13
**Figure S9**. ^{1}H (400 MHz) and ^{13}C (100 MHz) NMR spectra of **2a** in $CDCl_3$.	14
**Figure S10**. ^{1}H (400 MHz) and ^{13}C (100 MHz) NMR spectra of **2b** in $CDCl_3$.	15
**Figure S11**. ^{1}H (400 MHz) and ^{13}C (100 MHz) NMR spectra of **2c** in $CDCl_3$.	16
**Figure S12**. ^{1}H (400 MHz) and ^{13}C (100 MHz) NMR spectra of **2d** in $CDCl_3$.	17
**Figure S13**. ^{1}H (400 MHz) and ^{13}C (100 MHz) NMR spectra of **2e** in $CDCl_3$.	18
**Figure S14**. ^{1}H (400 MHz) and ^{13}C (100 MHz) NMR spectra of **2f** in $CDCl_3$.	19
**Figure S15**. ^{1}H (400 MHz) and ^{13}C (100 MHz) NMR spectra of **2g** in $CDCl_3$.	20
**Figure S16**. ^{1}H (400 MHz) and ^{13}C (100 MHz) NMR spectra of **2h** in $CDCl_3$.	21
**Figure S17**. ^{1}H (400 MHz) and ^{13}C (100 MHz) NMR spectra of **3a** in $CDCl_3$.	22
**Figure S18**. ^{1}H (400 MHz) and ^{13}C (100 MHz) NMR spectra of **3b** in $CDCl_3$.	23
**Figure S19**. ^{1}H (400 MHz) and ^{13}C (100 MHz) NMR spectra of **4** in $CDCl_3$.	24
**Figure S20**. ^{1}H (400 MHz) and ^{13}C (100 MHz) NMR spectra of **5** in $CDCl_3$.	25
**Figure S21**. ^{1}H (400 MHz) and ^{13}C (100 MHz) NMR spectra of **6** in $CDCl_3$.	26
**Figure S22**. ^{1}H (400 MHz) and ^{13}C (100 MHz) NMR spectra of **7** in $CDCl_3$.	27
**Figure S23**. ^{1}H (400 MHz) and ^{13}C (100 MHz) NMR spectra of **8** in $CDCl_3$.	28
**Figure S24.** Molecular structure of **5** obtained from X-ray crystallography.	29

Characterization of the synthesized compounds

Quinolin-8-yl benzenesulfonate (1a). Yield = 55%; ^{1}H NMR ($CDCl_3$, 400 MHz) δ7.40 (dd, 1H, *J* = 8.4, 4.0 Hz, Ar-H), 7.47-7.55 (m, 3H, Ar-H), 7.60-7.66 (m, 2H, Ar-H), 7.76-7.79 (m, 1H, Ar-H), 8.00-8.02 (m, 2H, Ar-H), 8.14-8.16 (m, 1H, Ar-H), 8.80 (dd, 1H, *J* = 4.0, 1.6 Hz, Ar-H); ^{13}C NMR ($CDCl_3$, 100 MHz) δ 150.71, 145.41, 141.38, 136.13, 135.84, 133.95, 129.62, 128.80, 128.76, 127.09, 126.05, 122.66, 121.87; M.p. = 112.5-113.6 °C; HRMS (ESI): Mass calculated for $C_{15}H_{12}NO_3S$ $[M+H]^+$: 286.0532; found 286.0546.

Quinolin-8-yl 2-bromobenzenesulfonate (1b). Yield = 79%; ^{1}H NMR ($CDCl_3$, 400 MHz) δ 7.40-7.43 (m, 2H, Ar-H), 7.47-7.55 (m, 3H, Ar-H), 7.78 (d, 1H, *J* = 7.6 Hz, Ar-H), 7.87 (d, 1H, *J* = 7.6 Hz, Ar-H), 8.05 (d, 1H, *J* = 8.0 Hz, Ar-H), 8.16 (d, 1H, *J* = 7.2 Hz, Ar-H), 8.78 (d, 1H, *J* = 2.8 Hz, Ar-H); ^{13}C NMR ($CDCl_3$, 100 MHz) δ 150.79, 145.80, 141.62, 136.97, 135.63, 135.57, 134.51, 132.20, 129.67, 127.26, 127.17, 125.96, 122.57, 121.94, 121.86; M.p. = 104.2-105.9 °C; HRMS (ESI): Mass calculated for $C_{15}H_{11}BrNO_3S$ $[M+H]^+$: 363.9638; found 363.9647.

Quinolin-8-yl 3-bromobenzenesulfonate (1c). Yield = 96%; ^{1}H NMR ($CDCl_3$, 400 MHz) δ 7.32-7.36 (m, 1H, Ar-H), 7.41-7.58 (m, 1H, Ar-H), 7.69-7.74 (m, 1H, Ar-H), 7.79-7.81 (m, 2H, Ar-H), 7.93 (d, 1H, *J* = 8.0 Hz, Ar-H), 8.17 (dd, 1H, *J* = 8.0, 1.6 Hz, Ar-H), 8.24-8.25 (m, 1H, Ar-H), 8.82 (dd, 1H, *J* = 4.0, 1.6 Hz, Ar-H); ^{13}C NMR ($CDCl_3$, 100 MHz) δ 150.75, 145.23, 141.20, 137.84, 136.91, 135.88, 131.89, 130.11, 129.67, 127.32, 126.13, 122.92, 122.61, 122.0; M.p. = 119.5-120.3 °C; HRMS (ESI): Mass calculated for $C_{15}H_{11}BrNO_3S$ $[M+H]^+$: 363.9638; found 363.9648.

Quinolin-8-yl 4-bromobenzenesulfonate (1d). Yield = 44%; ^{1}H NMR ($CDCl_3$, 400 MHz) δ 7.41-7.44 (m, 1H, Ar-H), 7.53-7.69 (m, 4H, Ar-H), 7.78-7.80 (m, 1H, Ar-H), 7.88 (d, 2H, *J* = 8.4 Hz, Ar-H), 8.17 (dd, 1H, *J* = 8.4, 1.6 Hz, Ar-H), 8.79 (dd, 1H, *J* = 4.0, 1.6 Hz, Ar-H); ^{13}C NMR ($CDCl_3$, 100 MHz) δ 150.73, 145.30, 141.26, 135.83, 135.27, 132.04, 130.31, 129.66, 129.21, 127.23, 126.09, 122.81, 121.94; M.p. = 114.4-115.5 °C; HRMS (ESI): Mass calculated for $C_{15}H_{11}BrNO_3S$ $[M+H]^+$: 363.9638; found 363.9645.

Quinolin-8-yl 3-(trifluoromethyl)benzenesulfonate (1e). Yield = 93%; ^{1}H NMR ($CDCl_3$, 400 MHz) δ 7.40 (dd, 1H, *J* = 8.4, 4.0 Hz, Ar-H), 7.56-7.64 (m, 2H, Ar-H), 7.75-7.76 (m, 1H, Ar-H), 7.80-7.82 (m, 1H, Ar-H), 7.85 (d, 1H, *J* = 8.0 Hz, Ar-H), 8.14-8.21 (m, 2H, Ar-H), 8.37 (s, 1H, Ar-H), 8.71 (dd, 1H, *J* = 4.0, 1.6 Hz, Ar-H); ^{13}C NMR ($CDCl_3$, 100 MHz) δ 150.64, 145.06, 140.92, 137.22, 135.98, 132.02, 130.47, 130.43, 129.65, 129.44, 127.48, 126.36, 126.32, 126.19, 123.20, 121.97; M.p. = 73.4-75.6 °C; HRMS (ESI): Mass calculated for $C_{16}H_{11}F_3NO_3S$ $[M+H]^+$: 354.0406; found 354.0419.

Quinolin-8-yl 4-(trifluoromethyl)benzenesulfonate (1f). Yield = 44%; ^{1}H NMR ($CDCl_3$, 400 MHz) δ 7.41 (dd, 1H, *J* = 8.4, 4.0 Hz, Ar-H), 7.55-7.60 (m, 1H, Ar-H), 7.70-7.76 (m, 3H, Ar-H), 7.80-7.82 (m, 1H, Ar-H), 8.14-8.18 (m, 3H, Ar-H), 8.71 (dd, 1H, *J* = 4.4, 1.6 Hz, Ar-H); ^{13}C NMR ($CDCl_3$, 100 MHz) δ 150.65, 145.14, 135.95, 135.34, 129.67, 129.43, 127.43, 126.18, 125.85, 125.81, 123.04, 121.98; M.p. = 134.2-135.1 ^{o}C; HRMS (ESI): Mass calculated for $C_{16}H_{11}F_3NO_3S$ $[M+H]^+$: 354.0406; found 354.0420.

Quinolin-8-yl 4-bromo-2-methylbenzenesulfonate (1g). Yield = 59%; ^{1}H NMR ($CDCl_3$, 400 MHz) δ 2.94 (s, 3H, CH_3), 7.34-7.37 (m, 1H, Ar-H), 7.42 (dd, 1H, *J* = 8.4, 4.0 Hz, Ar-H), 7.49-7.60 (m, 1H, Ar-H), 7.58-7.60 (m, 2H, Ar-H), 7.70 (d, 1H, *J* = 8.4 Hz, Ar-H), 7.75-7.77 (m, 1H, Ar-H), 8.14-8.16 (m, 1H, Ar-H), 8.78 (dd, 1H, *J* = 4.0, 1.6 Hz, Ar-H); ^{13}C NMR ($CDCl_3$, 100 MHz) δ 150.63, 145.45, 141.89, 141.31, 135.81, 135.22, 134.25, 131.65, 129.62, 128.91, 128.83, 127.02, 126.05, 122.47, 121.96, 20.78; M.p. = 133.9-135.3 ^{o}C; HRMS (ESI): Mass calculated for $C_{16}H_{13}BrNO_3S$ $[M+H]^+$: 377.9794; found 377.9802.

Quinolin-8-yl 4-(bromomethyl)benzenesulfonate (1h). Yield = 42%; ^{1}H NMR ($CDCl_3$, 400 MHz) δ 4.48 (s, 2H, CH_2), 7.39-7.42 (m, 1H, Ar-H), 7.46 (d, 1H, Ar-H), 7.54-7.58 (m, 1H, Ar-H), 7.69 (d, 1H, *J* = 6.8 Hz, Ar-H), 7.79 (d, 1H, *J* = 8.0 Hz, Ar-H), 7.96 (d, 2H, *J* = 8.4 Hz, Ar-H), 8.16 (dd, 1H, *J* = 8.4, 1.2 Hz, Ar-H), 8.77 (dd, 1H, *J* = 4.0, 1.2 Hz, Ar-H); ^{13}C NMR ($CDCl_3$, 100 MHz) δ 150.74, 145.30, 144.00, 141.24, 135.83, 129.61, 129.32, 127.19, 126.11, 122.92, 121.93, 31.35; M.p. = 146.3-147.6 ^{o}C; HRMS (ESI): Mass calculated for $C_{16}H_{13}BrNO_3S$ $[M+H]^+$: 377.9794; found 377.9804.

2-Methylquinolin-8-yl benzenesulfonate (2a). Yield = 57%; ^{1}H NMR ($CDCl_3$, 400 MHz) δ 2.54 (s, 3H, CH_3), 7.23 (d, 1H, *J* = 8.4 Hz, Ar-H), 7.45-7.50 (m, 3H, Ar-H), 7.58-7.62 (m, 1H, Ar-H), 7.67-7.73 (m, 2H, Ar-H), 8.01 (dd, 3H, *J* = 8.4, 6.8 Hz, Ar-H); ^{13}C NMR ($CDCl_3$, 100 MHz) δ 159.64, 144.91, 140.73, 136.48, 135.81, 133.78, 128.90, 128.52, 127.86, 126.83, 125.13, 123.16, 122.72, 25.23; M.p. = 112.9-113.5 ^{o}C; HRMS (ESI): Mass calculated for $C_{16}H_{14}NO_3S$ $[M+H]^+$: 300.0689; found 300.0701.

2-Methylquinolin-8-yl 2-bromobenzenesulfonate (2b). Yield = 72%; ^{1}H NMR ($CDCl_3$, 400 MHz) δ 2.44 (s, 3H, CH_3), 7.22 (d, 1H, *J* = 8.8 Hz, Ar-H), 7.34 (t, 1H, *J* = 7.6 Hz, Ar-H), 7.43-7.48 (m, 2H, Ar-H), 7.62 (d, 1H, *J* = 7.2 Hz, Ar-H), 7.71 (d, 1H, *J* = 8.0 Hz, Ar-H), 7.86 (d, 1H, *J* = 8.0 Hz, Ar-H), 7.95-8.00 (m, 2H, Ar-H); ^{13}C NMR ($CDCl_3$, 100 MHz) δ 159.73, 145.41, 140.87, 137.56, 135.59, 135.38, 134.18, 132.04, 127.85, 126.98, 126.89, 125.07, 123.38, 122.78, 122.19, 25.02; M.p. = 113.1-113.7 ^{o}C;

HRMS (ESI): Mass calculated for $C_{16}H_{13}BrNO_3S$ $[M+H]^+$: 377.9794; found 377.9809.

2-Methylquinolin-8-yl 3-bromobenzenesulfonate (2c). Yield = 91%; ^{1}H NMR ($CDCl_3$, 400 MHz) δ 2.60 (s, 3H, CH_3), 7.26-7.33 (m, 2H, Ar-H), 7.48-7.54 (m, 1H, Ar-H), 7.71-7.75 (m, 3H, Ar-H), 7.90 (d, 1H, *J* = 7.6 Hz, Ar-H), 8.03 (d, 1H, *J* = 8.8 Hz, Ar-H), 8.31 (s, 1H, Ar-H); ^{13}C NMR ($CDCl_3$, 100 MHz) δ 159.92, 144.64, 140.57, 138.27, 136.77, 135.87, 131.89, 129.88, 127.95, 127.38, 127.10, 125.18, 123.30, 122.85, 122.49, 25.45; M.p. = 128.3-129.8 ^{o}C; HRMS (ESI): Mass calculated for $C_{16}H_{13}BrNO_3S$ $[M+H]^+$: 377.9794; found 377.9811.

2-Methylquinolin-8-yl 4-bromobenzenesulfonate (2d). Yield = 52%; ^{1}H NMR ($CDCl_3$, 400 MHz) δ 2.54 (s, 3H, CH_3), 7.25 (d, 1H, *J* = 8.4 Hz, Ar-H), 7.48-7.52 (m, 1H, Ar-H), 7.59 (d, 2H, *J* = 8.4 Hz, Ar-H), 7.69-7.74 (m, 2H, Ar-H), 7.86 (d, 2H, *J* = 8.8 Hz, Ar-H), 8.02 (d, 1H, *J* = 8.4 Hz, Ar-H); ^{13}C NMR ($CDCl_3$, 100 MHz) δ 159.70, 144.79, 140.65, 135.81, 135.50, 131.80, 130.44, 129.02, 127.90, 126.99, 125.19, 123.40, 122.76, 25.16; M.p. = 157.4-158.7 ^{o}C; HRMS (ESI): Mass calculated for $C_{16}H_{13}BrNO_3S$ $[M+H]^+$: 377.9794; found 377.9798.

2-Methylquinolin-8-yl 3-(trifluoromethyl)benzenesulfonate (2e). Yield = 93%; ^{1}H NMR ($CDCl_3$, 400 MHz) δ 2.48 (s, 3H, CH_3), 7.24 (d, 1H, *J* = 8.4 Hz, Ar-H), 7.50-7.54 (m, 1H, Ar-H), 7.60-7.63 (m, 1H, Ar-H), 7.74-7.77 (m, 2H, Ar-H), 7.85 (d, 1H, *J* = 8.0 Hz, Ar-H), 8.02 (d, 1H, *J* = 8.4 Hz, Ar-H), 8.20 (d, 1H, *J* = 8.0 Hz, Ar-H), 8.31 (s, 1H, Ar-H); ^{13}C NMR ($CDCl_3$, 100 MHz) δ 159.90, 144.59, 140.47, 137.72, 135.84, 132.08, 131.50, 131.17, 130.30, 129.27, 127.95, 127.20, 126.05, 125.18, 123.36, 122.83, 25.10; M.p. = 61.2-62.9 ^{o}C; HRMS (ESI): Mass calculated for $C_{17}H_{13}F_3NO_3S$ $[M+H]^+$: 368.0563; found 368.0578.

2-Methylquinolin-8-yl 4-(trifluoromethyl)benzenesulfonate (2f). Yield = 55%; ^{1}H NMR ($CDCl_3$, 400 MHz) δ 2.45 (s, 3H, CH_3), 7.23 (d, 1H, *J* = 8.4 Hz, Ar-H), 7.52 (t, 1H, *J* = 8.0 Hz, Ar-H), 7.71-7.76 (m, 4H, Ar-H), 8.01 (d, 1H, *J* = 8.4 Hz, Ar-H), 8.14 (d, 2H, *J* = 8.0 Hz, Ar-H); ^{13}C NMR ($CDCl_3$, 100 MHz) δ 159.72, 144.72, 140.49, 140.12, 135.84, 135.12, 129.50, 127.92, 127.14, 125.57, 125.53, 125.23, 124.49, 123.50, 122.78, 25.01; M.p. = 137.0-138.4 ^{o}C; HRMS (ESI): Mass calculated for $C_{17}H_{13}F_3NO_3S$ $[M+H]^+$: 368.0563; found 368.0579.

2-Methylquinolin-8-yl 4-bromo-2-methylbenzenesulfonate (2g). Yield = 28%; ^{1}H NMR ($CDCl_3$, 400 MHz) δ 2.51 (s, 3H, CH_3), 2.99 (s, 3H, CH_3), 7.22-7.28 (m, 2H, Ar-H), 7.44-7.48 (m, 1H, Ar-H), 7.56-7.71 (m, 4H, Ar-H), 7.99 (d, 1H, *J* = 8.4 Hz, Ar-H); ^{13}C NMR ($CDCl_3$, 100 MHz) δ 159.61, 144.99, 142.16, 135.70, 135.02, 134.49, 131.56, 128.67, 128.54, 127.81, 126.78, 125.09, 123.27, 122.77, 24.92, 21.05; M.p. =

106.9-107.6 °C; HRMS (ESI): Mass calculated for $C_{17}H_{15}BrNO_3S$ $[M+H]^+$: 391.9951; found 391.9964.

2-Methylquinolin-8-yl 4-(bromomethyl)benzenesulfonate (2h). Yield = 29%; ^{1}H NMR ($CDCl_3$, 400 MHz) δ 2.54 (s, 3H, CH_3), 4.47 (s, 2H, CH_2), 7.23 (d, 1H, *J* = 8.8 Hz, Ar-H), 7.45-7.51 (m, 3H, Ar-H), 7.70-7.74 (m, 2H, Ar-H), 7.95-8.01 (m, 3H, Ar-H); ^{13}C NMR ($CDCl_3$, 100 MHz) δ 159.72, 144.88, 143.62, 140.74, 136.33, 135.71, 129.46, 129.41, 129.01, 128.49, 127.85, 126.88, 125.14, 123.33, 122.76, 31.34, 25.35; M.p. = 144.6-145.9 °C; HRMS (ESI): Mass calculated for $C_{17}H_{15}BrNO_3S$ $[M+H]^+$: 391.9951; found 391.9962.

Quinolin-8-yl 5-bromothiophene-2-sulfonate (3a). Yield = 43%; ^{1}H NMR ($CDCl_3$, 400 MHz) δ 7.01 (d, 1H, *J* = 4.0 Hz, Ar-H), 7.44-7.47 (m, 2H, Ar-H), 7.54-7.59 (m, 1H, Ar-H), 7.70 (d, 1H, *J* = 7.6 Hz, Ar-H), 7.82 (d, 1H, *J* = 8.4 Hz, Ar-H), 8.19 (dd, 1H, *J* = 8.4, 1.6 Hz, Ar-H), 8.87 (dd, 1H, *J* = 4.0, 1.6 Hz, Ar-H); ^{13}C NMR ($CDCl_3$, 100 MHz) δ 150.78, 145.33, 141.20, 136.03, 135.77, 130.23, 129.65, 127.46, 126.17, 122.93, 122.81, 122.04; M.p. = 144.2-145.5 °C; Mass calculated for $C_{13}H_9BrNO_3S_2$ $[M+H]^+$: 369.9202; found 369.9200.

2-Methylquinolin-8-yl 5-bromothiophene-2-sulfonate (3b). Yield = 90%; ^{1}H NMR ($CDCl_3$, 400 MHz) δ 2.63 (s, 3H, CH_3), 6.95 (d, 1H, *J* = 4.0 Hz, Ar-H), 7.27-7.32 (m, 2H, Ar-H), 7.50 (t, 1H, *J* = 8.0 Hz, Ar-H), 7.69-7.76 (m, 2H, Ar-H), 8.04 (d, 1H, *J* = 8.4 Hz, Ar-H); ^{13}C NMR ($CDCl_3$, 100 MHz) δ 159.96, 144.83, 140.79, 135.82, 135.42, 129.99, 127.85, 127.17, 125.17, 123.48, 122.84, 122.36, 25.22; M.p. = 105.8-106.3 °C; HRMS (ESI): Mass calculated for $C_{14}H_{11}BrNO_3S_2$ $[M+H]^+$: 383.9364; found 383.9357.

Quinolin-8-yl quinoline-8-sulfonate (4). Yield = 44%; ^{1}H NMR (400 mHz, $CDCl_3$): δ 7.32 (dd, 1H, *J* = 8.4, 4.0 Hz, Ar-H), 7.52-7.57 (m, 2H, Ar-H), 7.62-7.66 (m, 1H, Ar-H), 7.74-7.79 (m, 2H, Ar-H), 8.10-8.17 (m, 2H, Ar-H), 8.30 (dd, 1H, *J* = 8.4, 1.6 Hz, Ar-H), 8.53 (dd, 1H, *J* = 4.4, 1.6 Hz, Ar-H), 8.58 (dd, 1H, *J* = 7.2, 1.2 Hz, Ar-H), 9.10-9.11 (m, 1H, Ar-H); ^{13}C NMR (100 mHz, $CDCl_3$): δ 151.83, 150.33, 146.28, 144.49, 141.56, 136.28, 135.73, 134.82, 134.52, 133.25, 129.51, 128.95, 126.71, 126.15, 125.12, 123.07, 122.25, 121.66; M.p. = 148.3-149.2 °C; HRMS (ESI): Mass calculated for $C_{18}H_{13}N_2O_3S$ $[M+H]^+$: 337.0641; found 337.0640.

N-(Quinolin-8-yl)quinolin-8-sulfonamide (5). Yield = 28%; ^{1}H NMR ($CDCl_3$, 400 MHz) δ 7.33-7.40 (m, 3H, Ar-H), 7.47 (dd, 1H, *J* = 8.4, 4.4 Hz, Ar-H), 7.56-7.60 (m, 1H, Ar-H), 7.95 (d, 1H, *J* = 8.0 Hz, Ar-H), 8.00-8.02 (m, 2H, Ar-H), 8.13 (d, 1H, *J* = 8.0 Hz, Ar-H), 8.55 (d, 1H, *J* = 7.2 Hz, Ar-H), 8.73-8.74 (m, 1H, Ar-H), 9.14-9.15 (m, 1H, Ar-H); ^{13}C NMR ($CDCl_3$, 100 MHz) δ 151.17, 148.06, 138.75, 136.24, 136.02,

135.91, 134.62, 133.48, 131.77, 128.68, 128.14, 126.89, 125.18, 122.11, 121.63, 114.95; M.p. = 236.7-237.5 °C; HRMS (ESI): Mass calculated for $C_{18}H_{14}N_3O_2S$ $[M+H]^+$: 336.0804; found 336.0801.

2-Methylquinolin-8-yl quinoline-8-sulfonate (6). Yield = 68%; ^{1}H NMR (400 mHz, $CDCl_3$): δ 2.05 (s, 3H, CH_3), 7.12 (d, 1H, *J* = 8.4 Hz, Ar-H), 7.49 (t, 1H, *J* = 8.0 Hz, Ar-H), 7.57-7.62 (m, 2H, Ar-H), 7.70 (d, 1H, *J* = 8.0 Hz, Ar-H), 7.78 (d, 1H, *J* = 7.6 Hz, Ar-H), 7.96 (d, 1H, *J* = 8.4 Hz, Ar-H), 8.13-8.15 (m, 1H, Ar-H), 8.30 (dd, 1H, *J* = 8.4, 1.6 Hz, Ar-H), 8.49-8.51 (m, 1H, Ar-H), 9.17-9.18 (m, 1H, Ar-H); ^{13}C NMR (100 mHz, $CDCl_3$): δ 159.13, 151.91, 145.64, 144.68, 136.21, 136.00, 134.47, 132.94, 128.96, 127.76, 126.45, 125.01, 123.79, 122.54, 122.22, 24.68; M.p. = 154.2-156.4 °C; HRMS (ESI): Mass calculated for $C_{19}H_{14}N_2SO_3Na$ $[M+Na]^+$: 373.0617; found 373.0630.

2-Formylquinolin-8-yl quinoline-8-sulfonate (7). Yield = 30%; ^{1}H NMR (400 mHz, $CDCl_3$): δ 7.58-7.62 (m, 2H, Ar-H), 7.72-7.76 (m, 1H, Ar-H), 7.83-7.86 (m, 2H, Ar-H), 7.93 (dd, 1H, *J* = 7.6, 1.2 Hz, Ar-H), 8.15-8.17 (m, 1H, Ar-H), 8.25 (d, 1H, *J* = 8.8 Hz, Ar-H), 8.30-8.33 (m, 1H, Ar-H), 8.45 (dd, 1H, *J* = 7.2, 1.2 Hz, Ar-H), 8.92 (s, 1H, CHO), 9.14 (dd, 1H, *J* = 4.0, 1.6 Hz, Ar-H); ^{13}C NMR (100 mHz, $CDCl_3$): δ 192.95, 152.09, 151.97, 147.04, 144.64, 141.36, 137.20, 136.35, 134.93, 134.75, 133.01, 131.00, 129.13, 128.94, 126.74, 125.13, 124.82, 122.43, 117.61; M.p. =143.3-143.7 °C; HRMS (ESI): Mass calculated for $C_{19}H_{13}N_2SO_4$ $[M+H]^+$: 365.0591; found 365.0605.

5,7-Dibromoquinolin-8-yl quinoline-8-sulfonate (8). Yield = 23%; ^{1}H NMR (400 mHz, $CDCl_3$): δ 7.42-7.45 (m, 1H, Ar-H), 7.61-7.66 (m, 2H, Ar-H), 8.03 (s, 1H, Ar-H), 8.19-8.21 (m, 1H, Ar-H), 8.33-8.44 (m, 4H, Ar-H), 9.26 (d, 1H, *J* = 2.8 Hz, Ar-H); ^{13}C NMR (100 mHz, $CDCl_3$): δ 158.36, 152.21, 151.29, 144.96, 144.43, 142.79, 136.42, 135.80, 134.77, 133.61, 132.46, 128.94, 127.73, 125.10, 122.92, 122.46; M.p. =198.3-200.0 °C; HRMS (ESI): Mass calculated for $C_{18}H_{11}N_2SO_3Br_2$ $[M+H]^+$: 492.8852; found 492.8861.

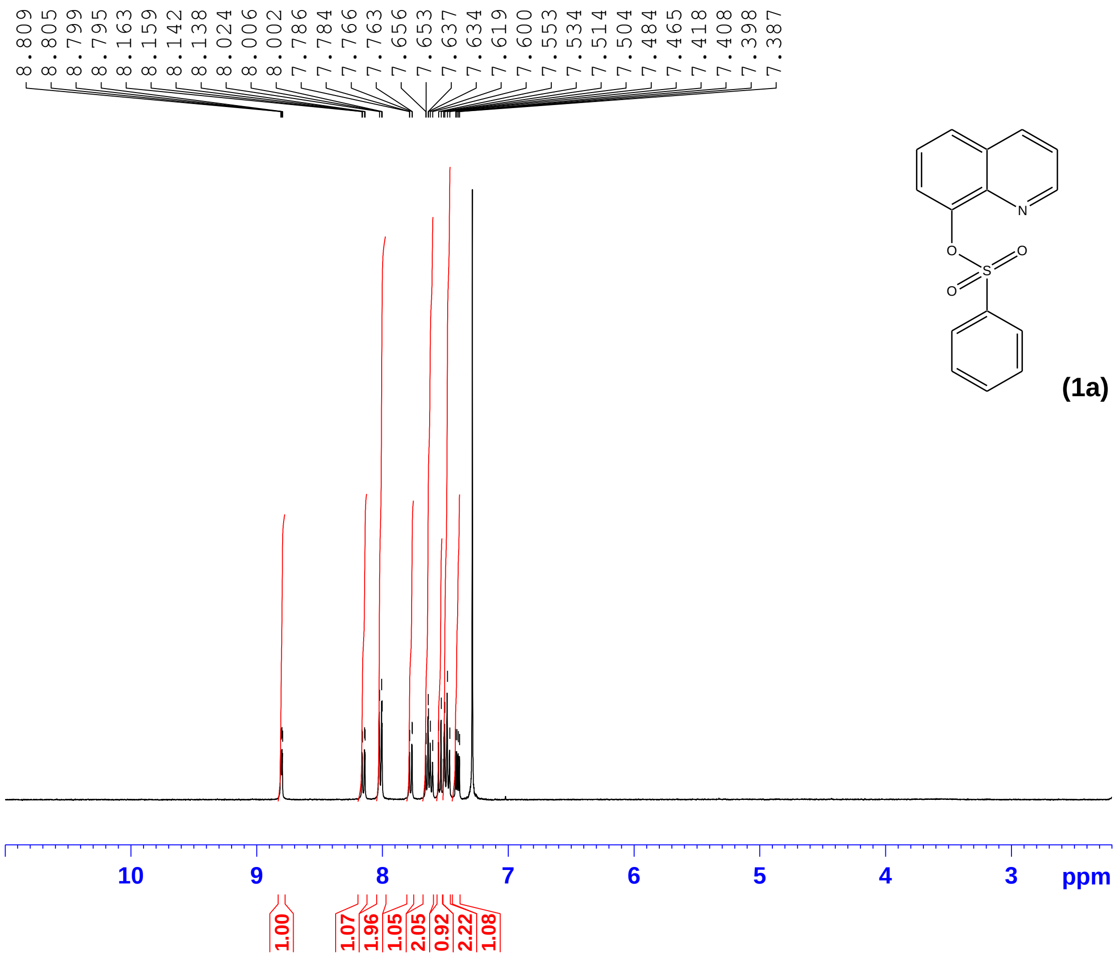

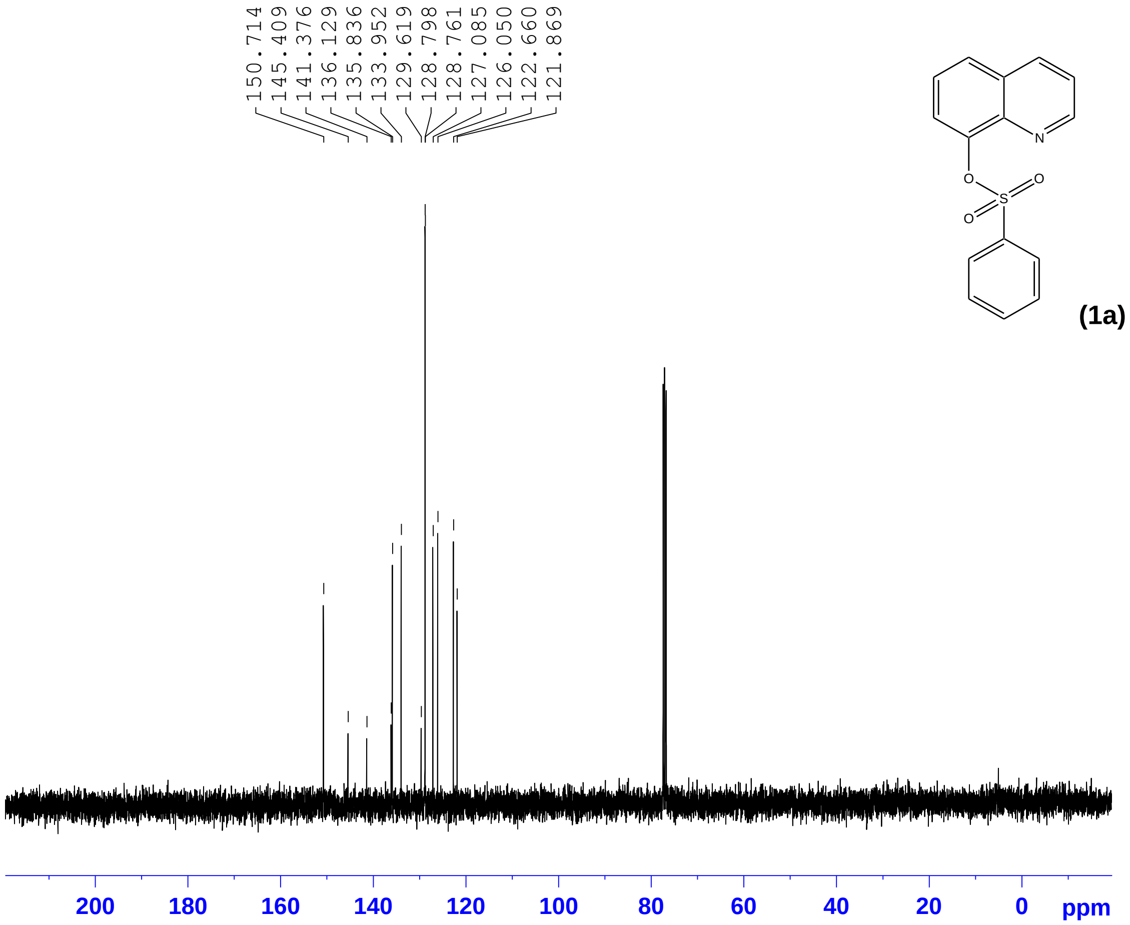

Figure S1. ^{1}H (400 MHz) and ^{13}C (100 MHz) NMR spectra of **1a** in $CDCl_3$.

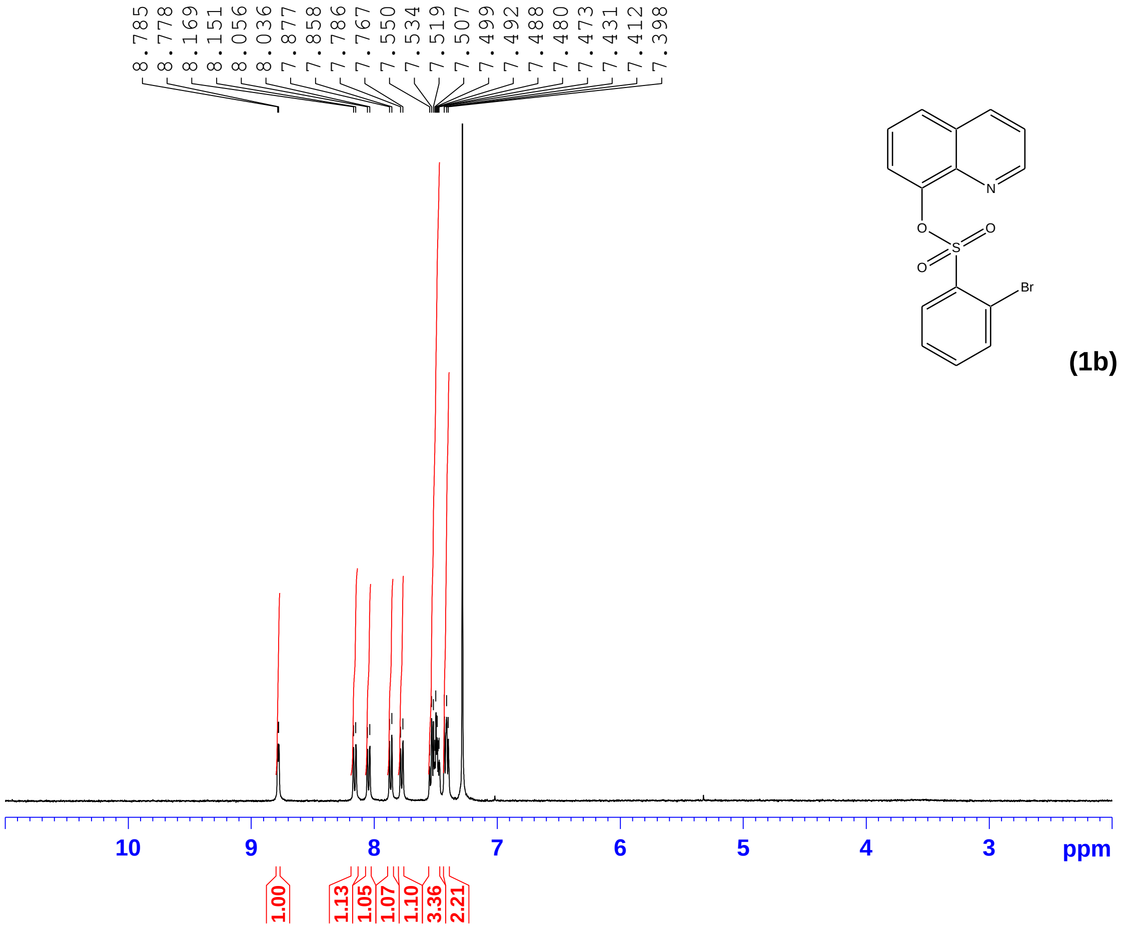

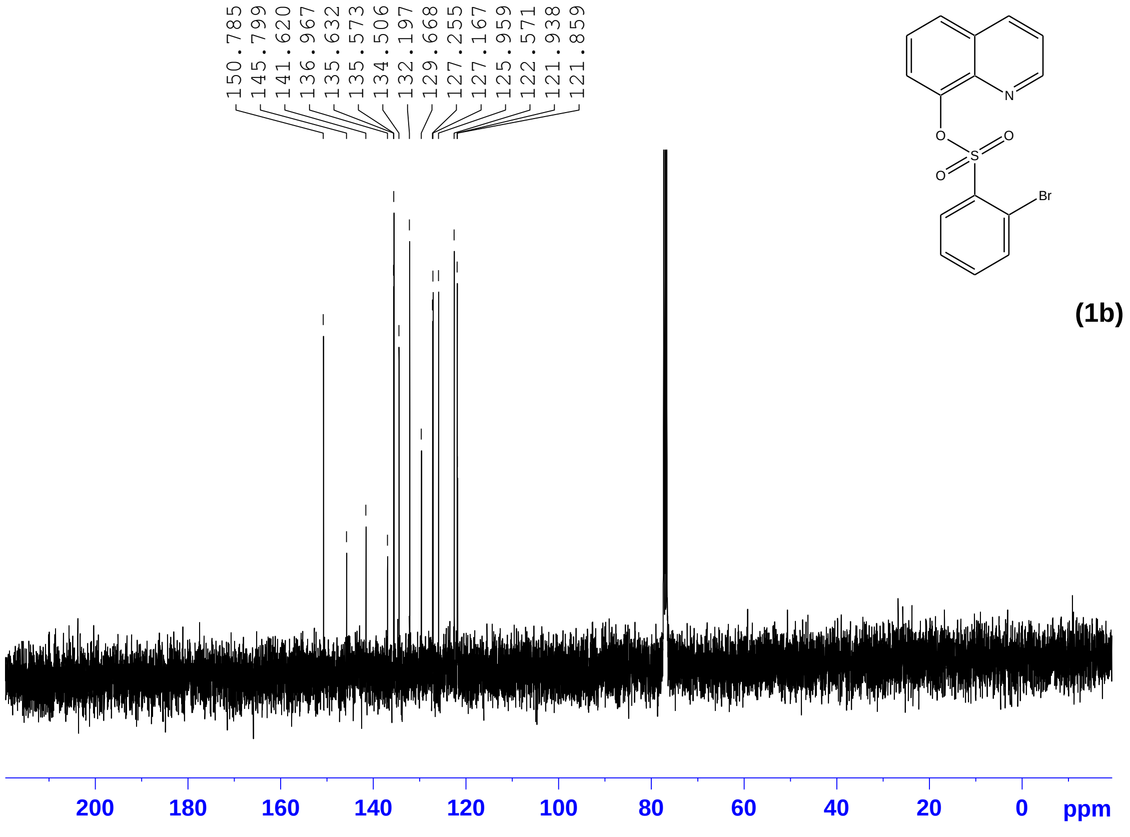

Figure S2. ^{1}H (400 MHz) and ^{13}C (100 MHz) NMR spectra of **1b** in $CDCl_3$.

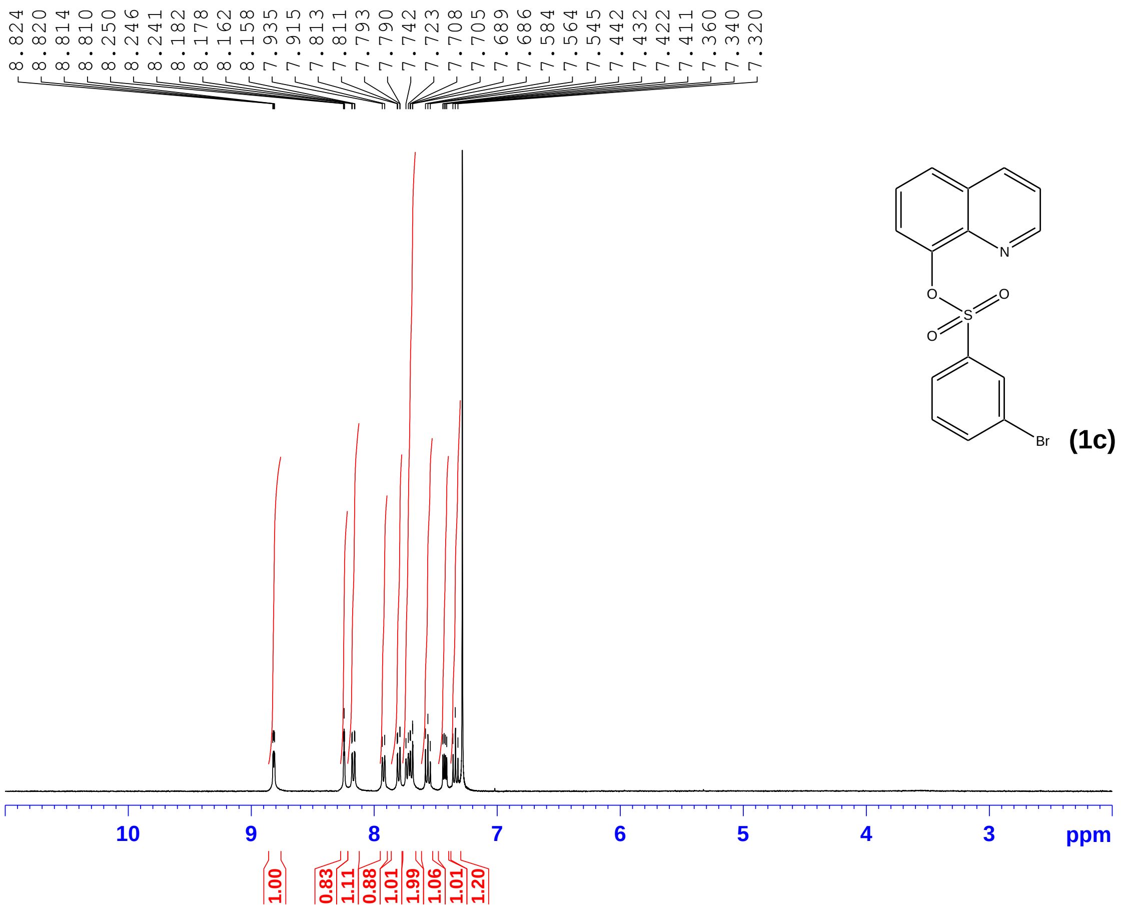

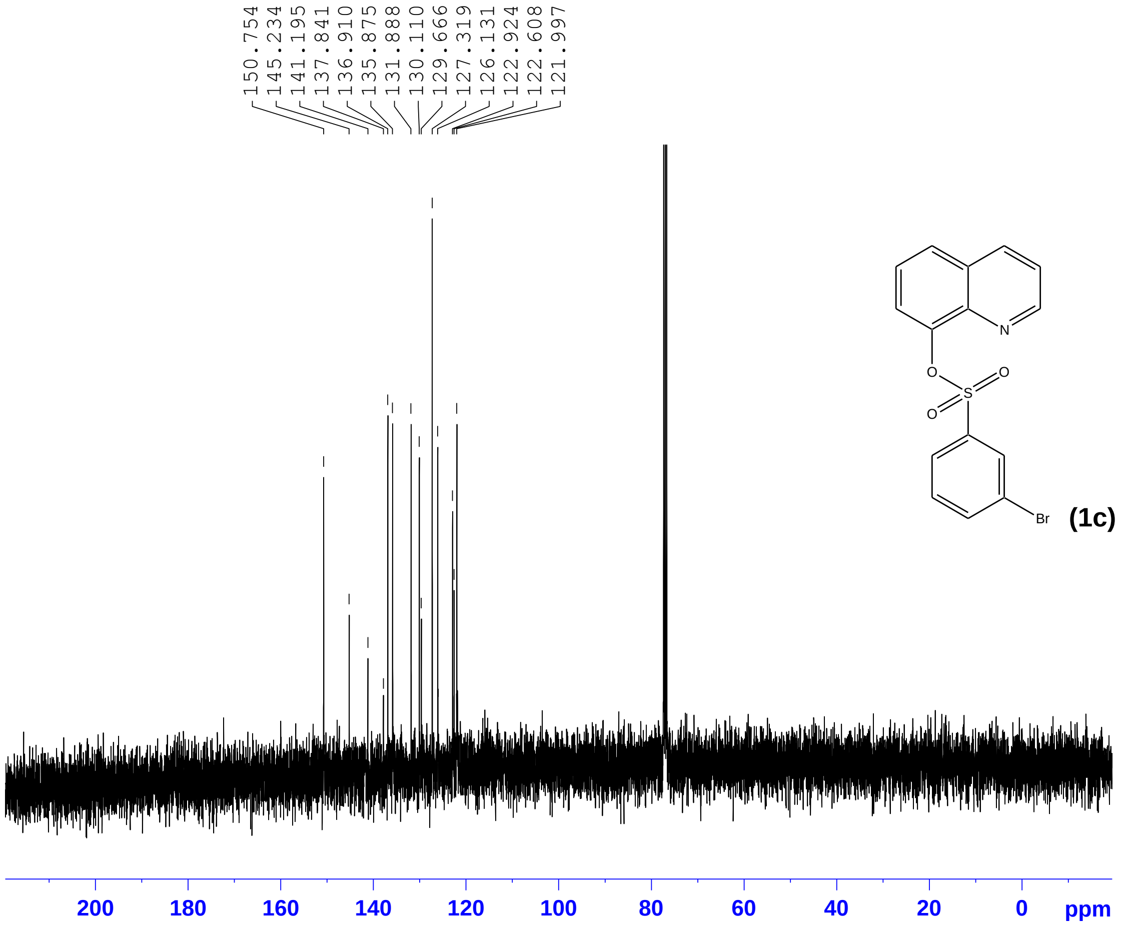

Figure S3. ^{1}H (400 MHz) and ^{13}C (100 MHz) NMR spectra of **1c** in $CDCl_3$.

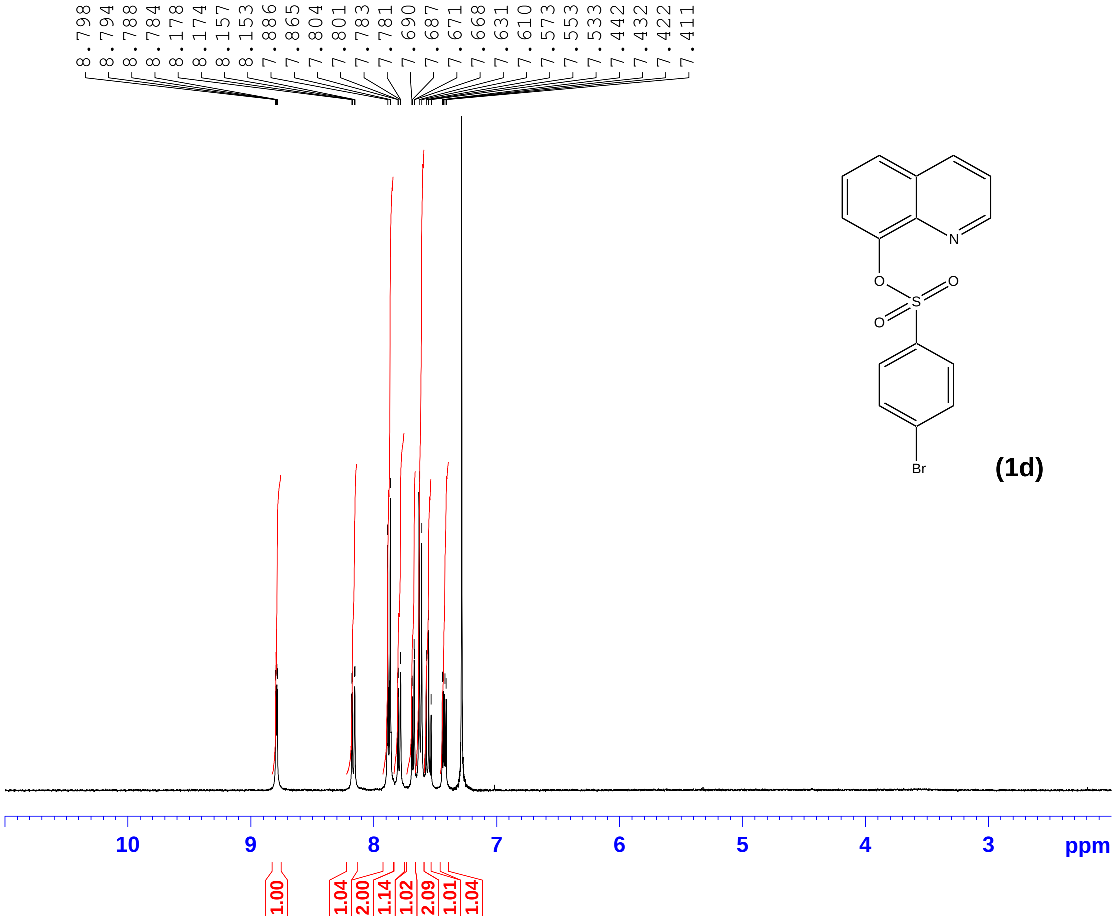

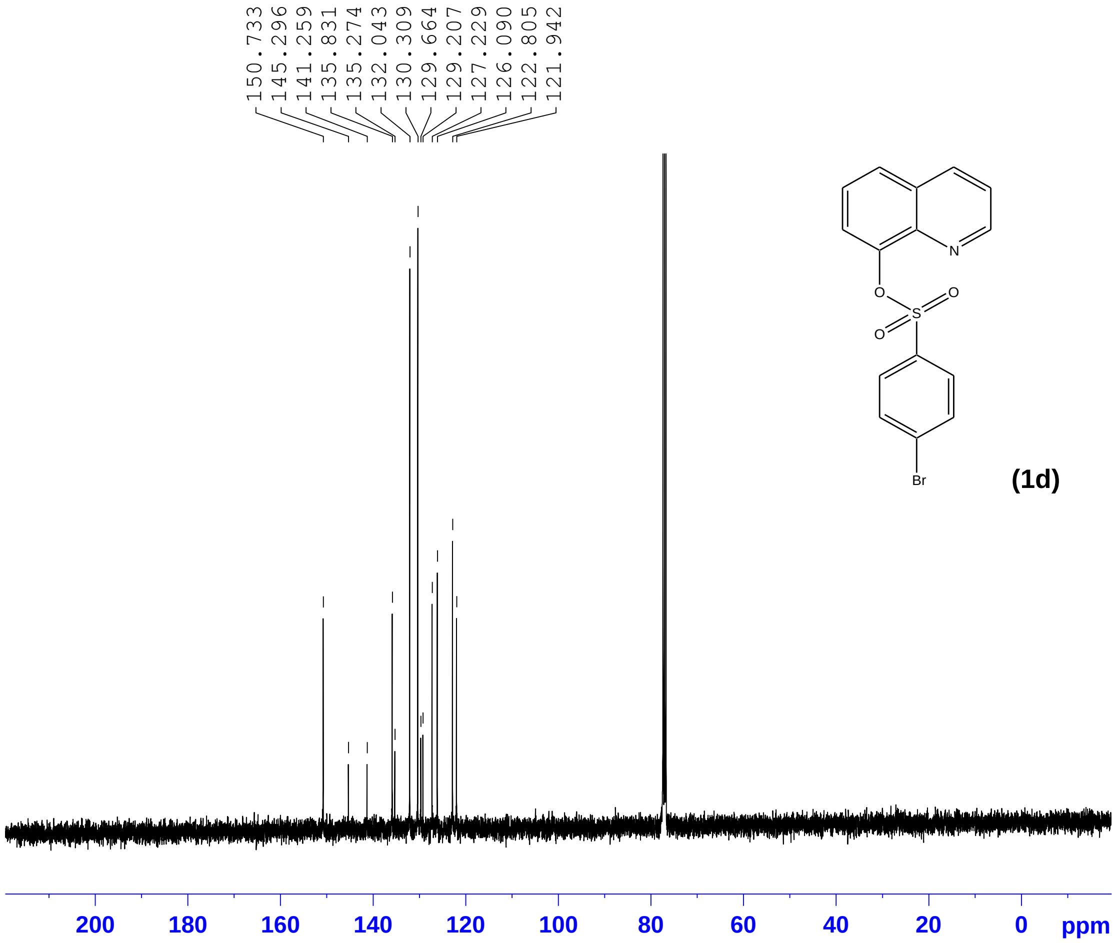

Figure S4. ^{1}H (400 MHz) and ^{13}C (100 MHz) NMR spectra of **1d** in $CDCl_3$.

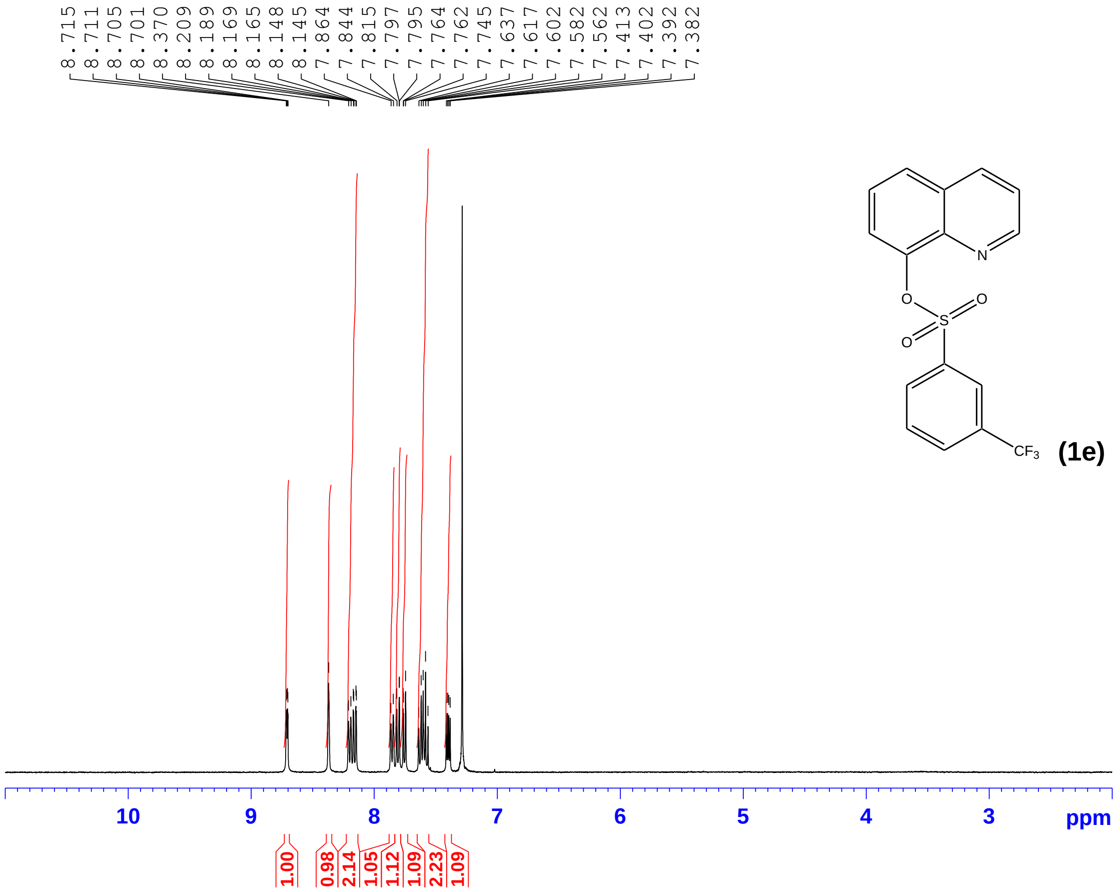

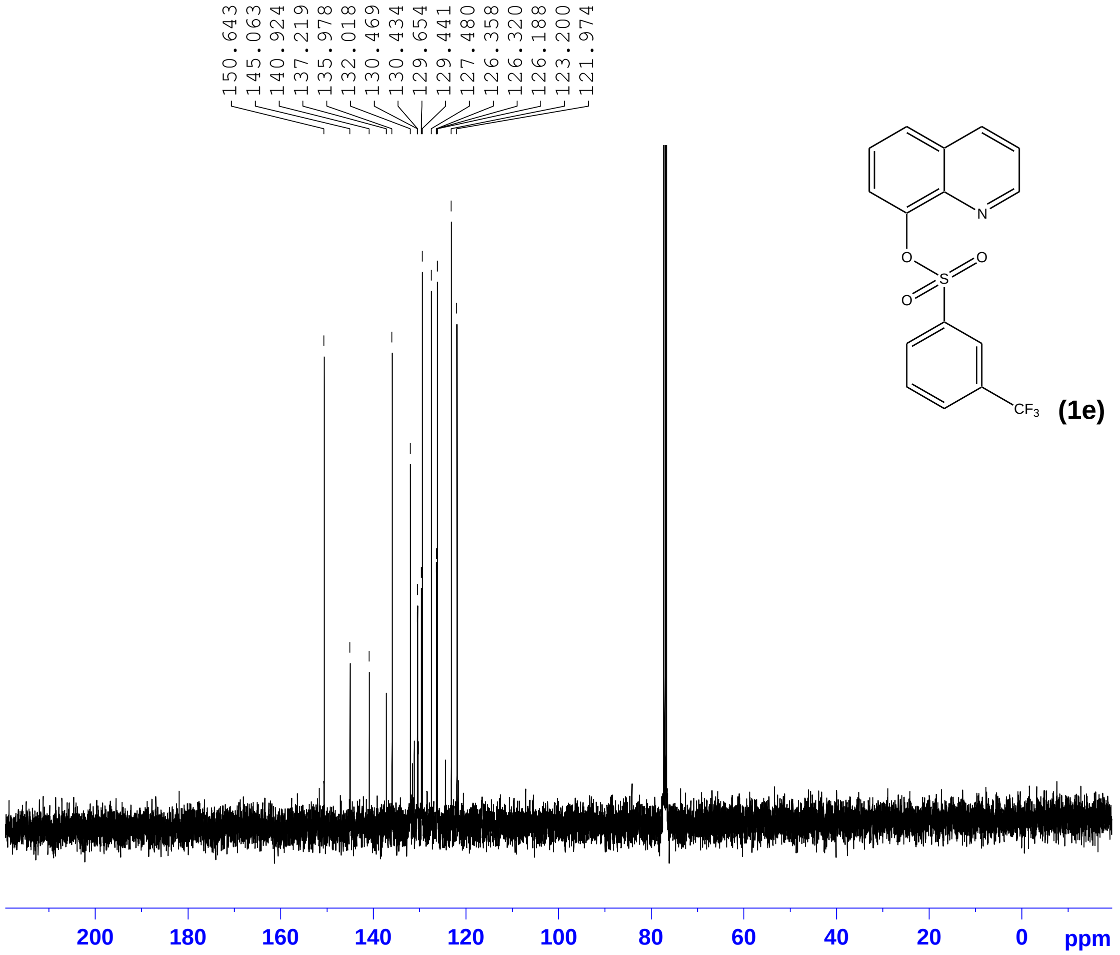

Figure S5. ^{1}H (400 MHz) and ^{13}C (100 MHz) NMR spectra of **1e** in $CDCl_3$.

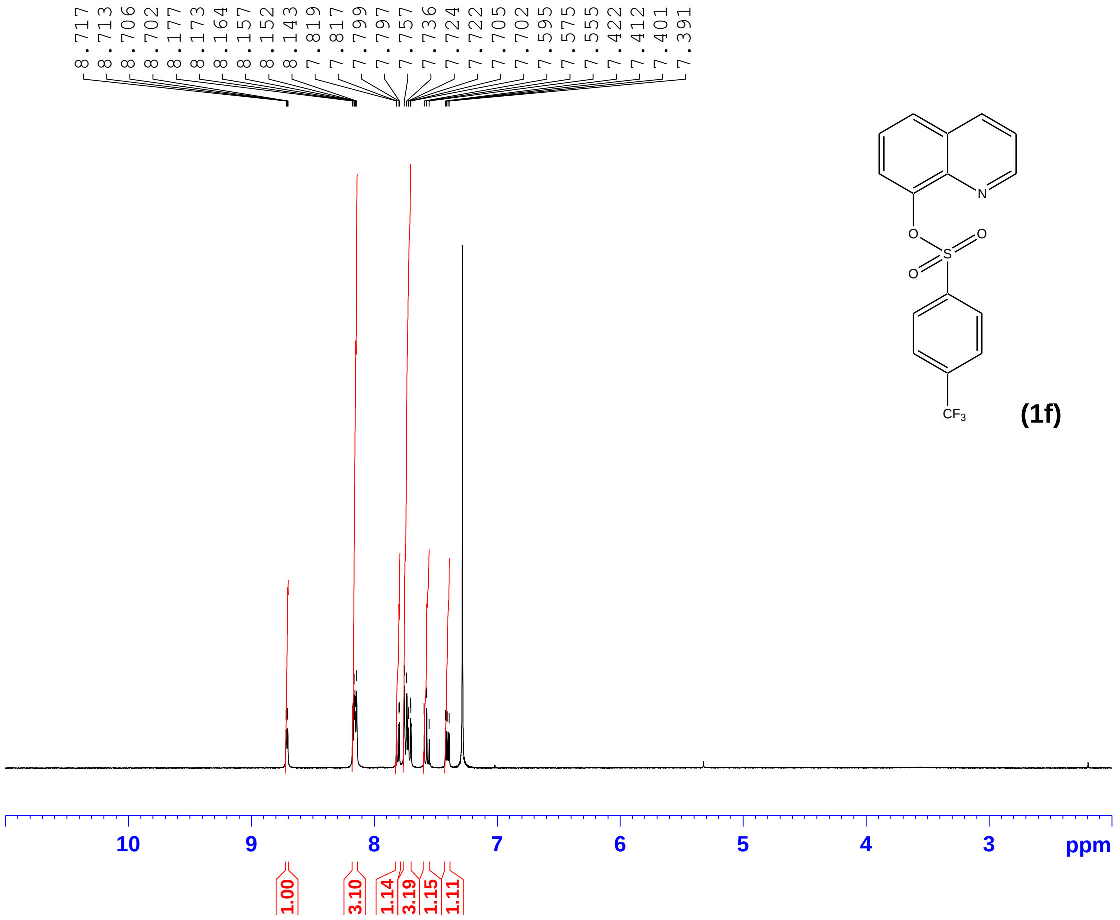

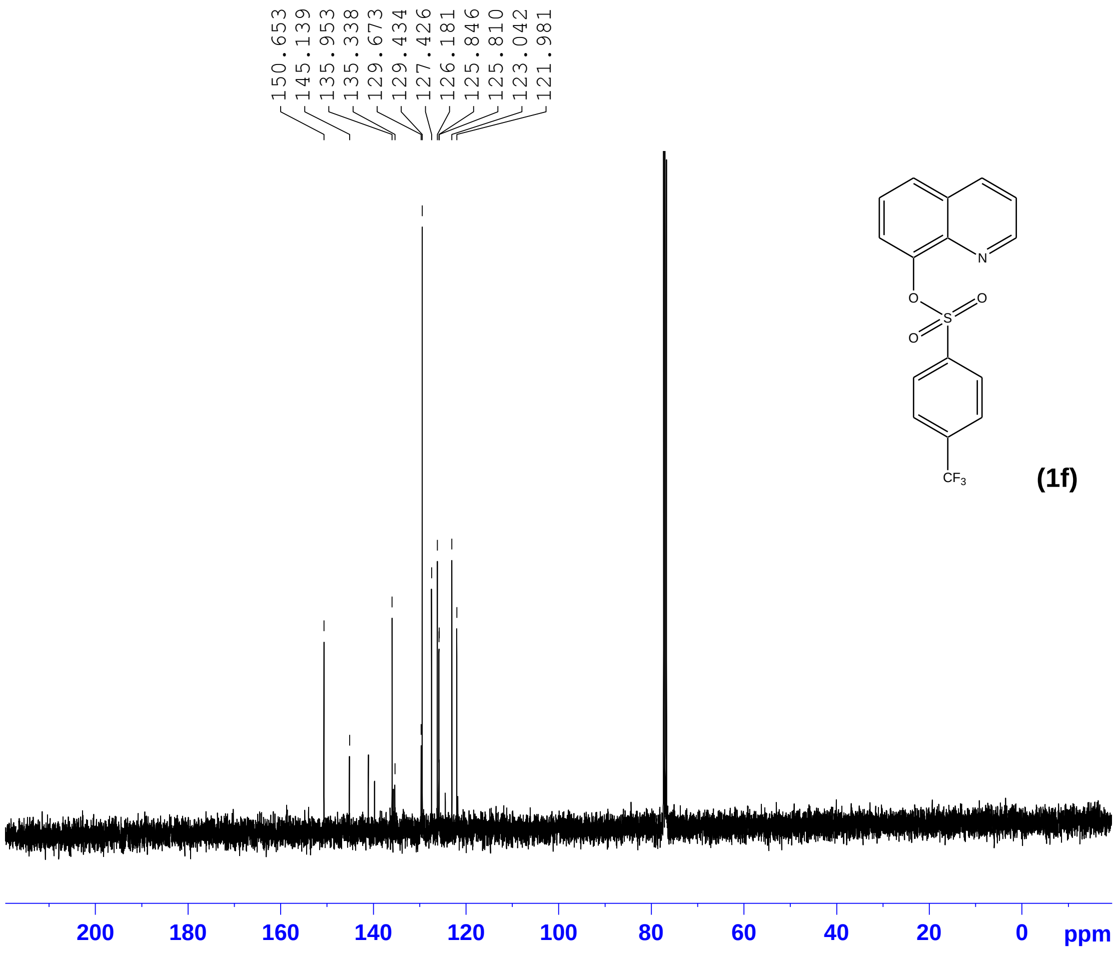

Figure S6. ^{1}H (400 MHz) and ^{13}C (100 MHz) NMR spectra of **1f** in $CDCl_3$.

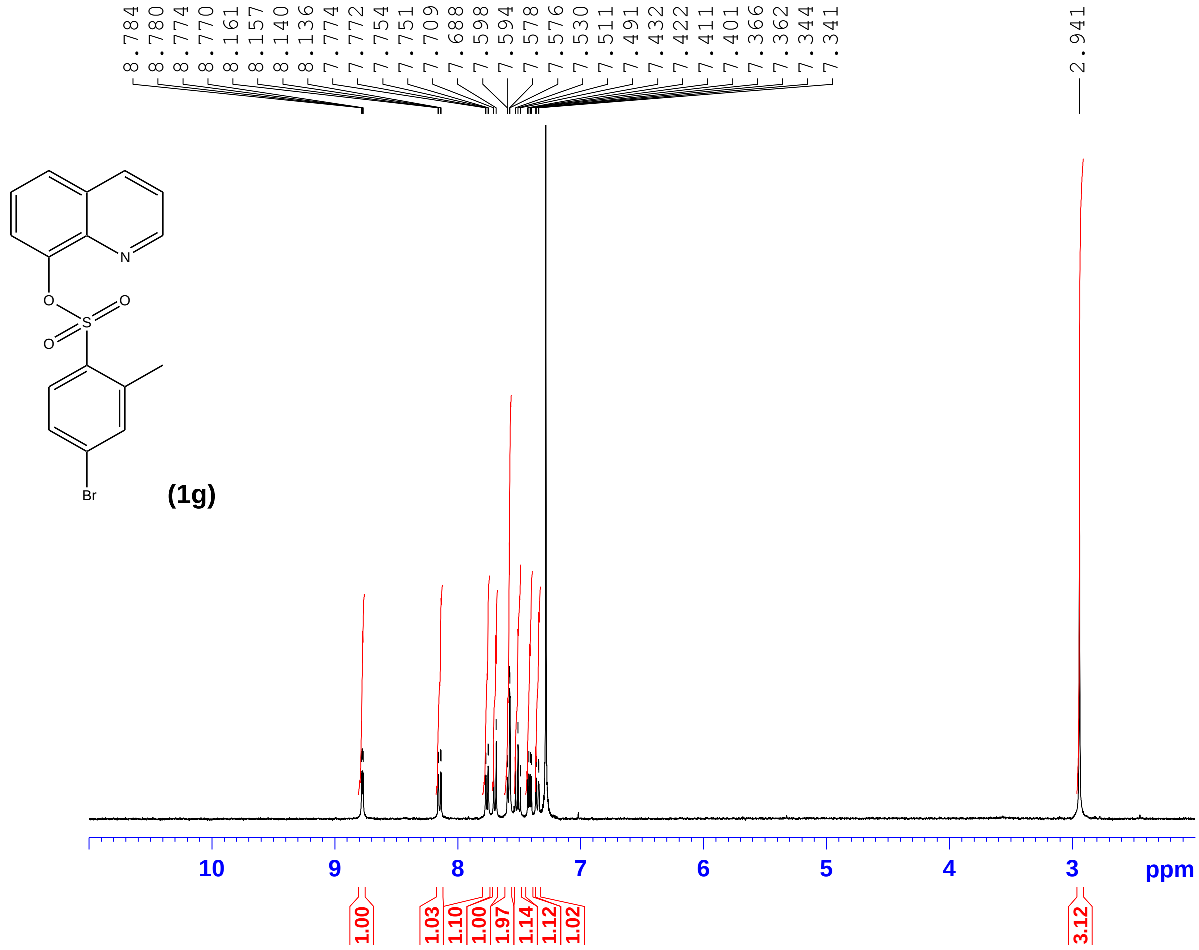

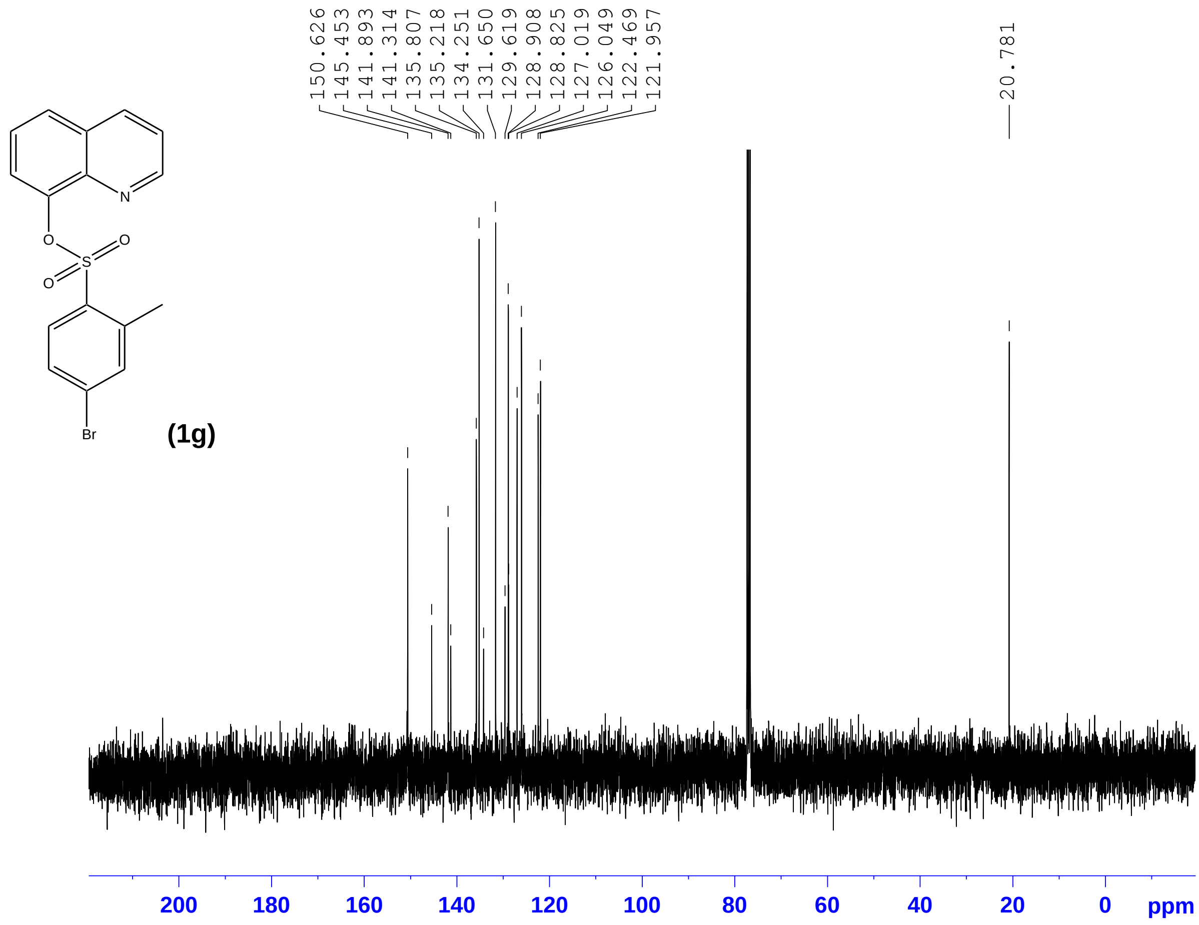

Figure S7. ^{1}H (400 MHz) and ^{13}C (100 MHz) NMR spectra of **1g** in $CDCl_3$.

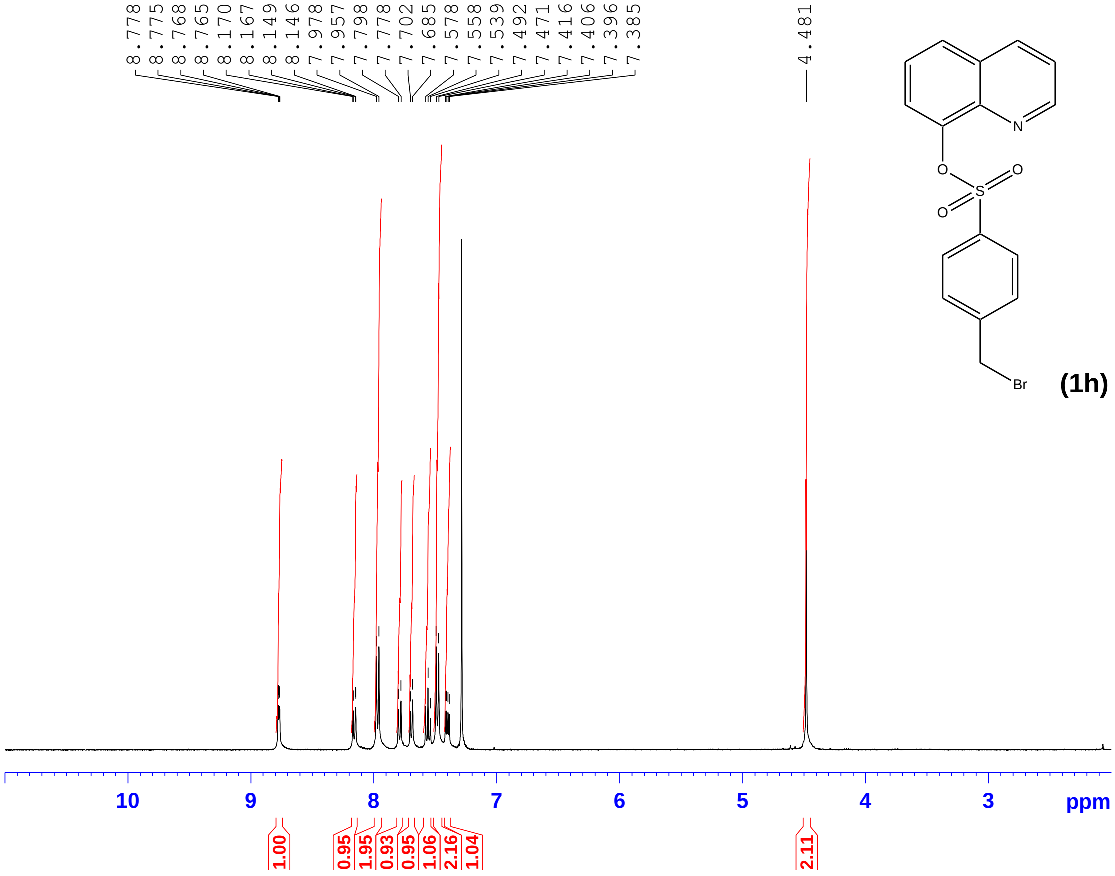

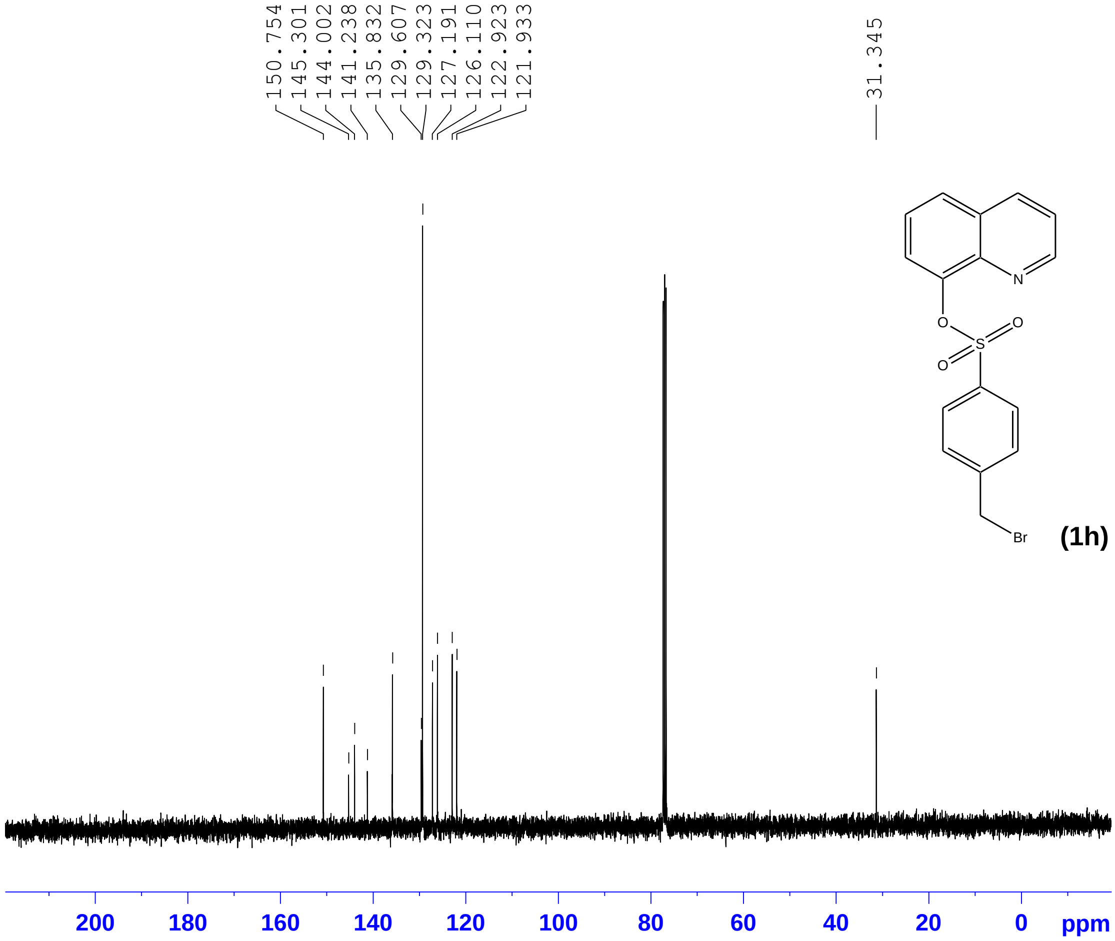

Figure S8. ^{1}H (400 MHz) and ^{13}C (100 MHz) NMR spectra of **1h** in $CDCl_3$.

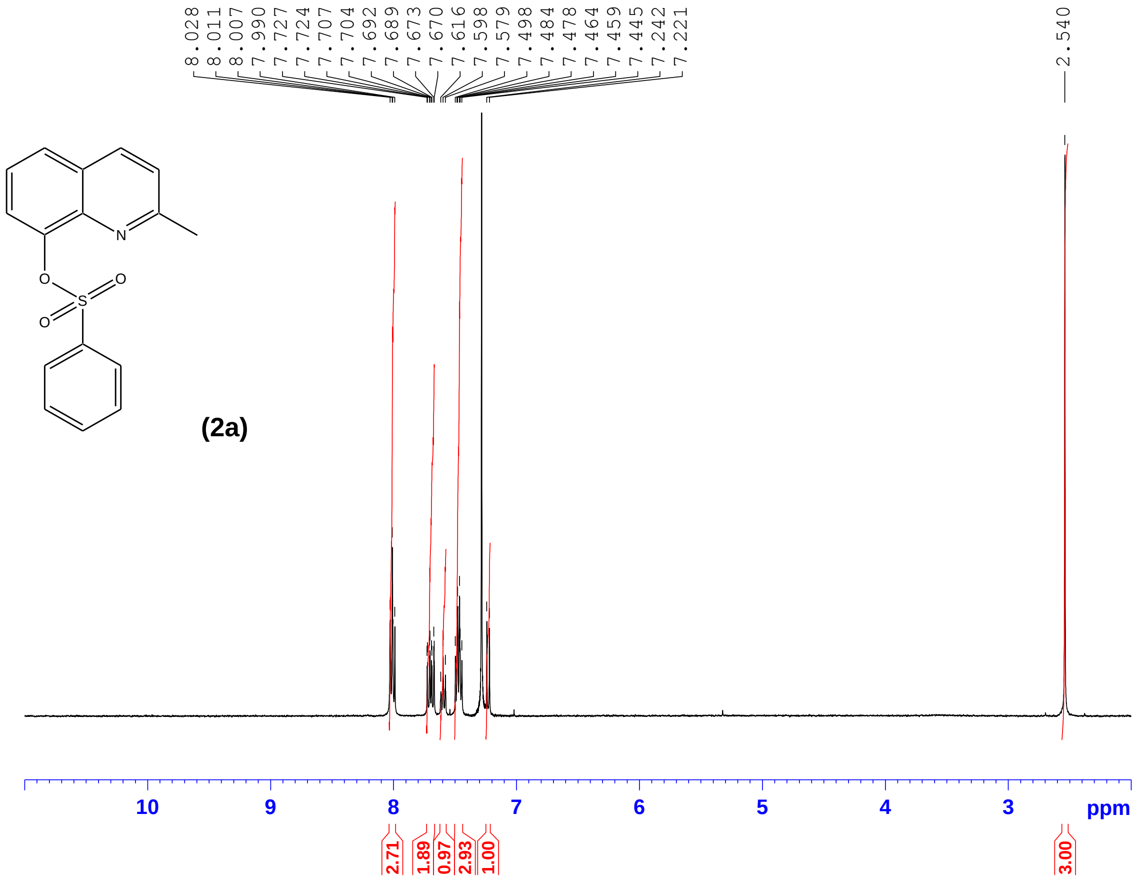

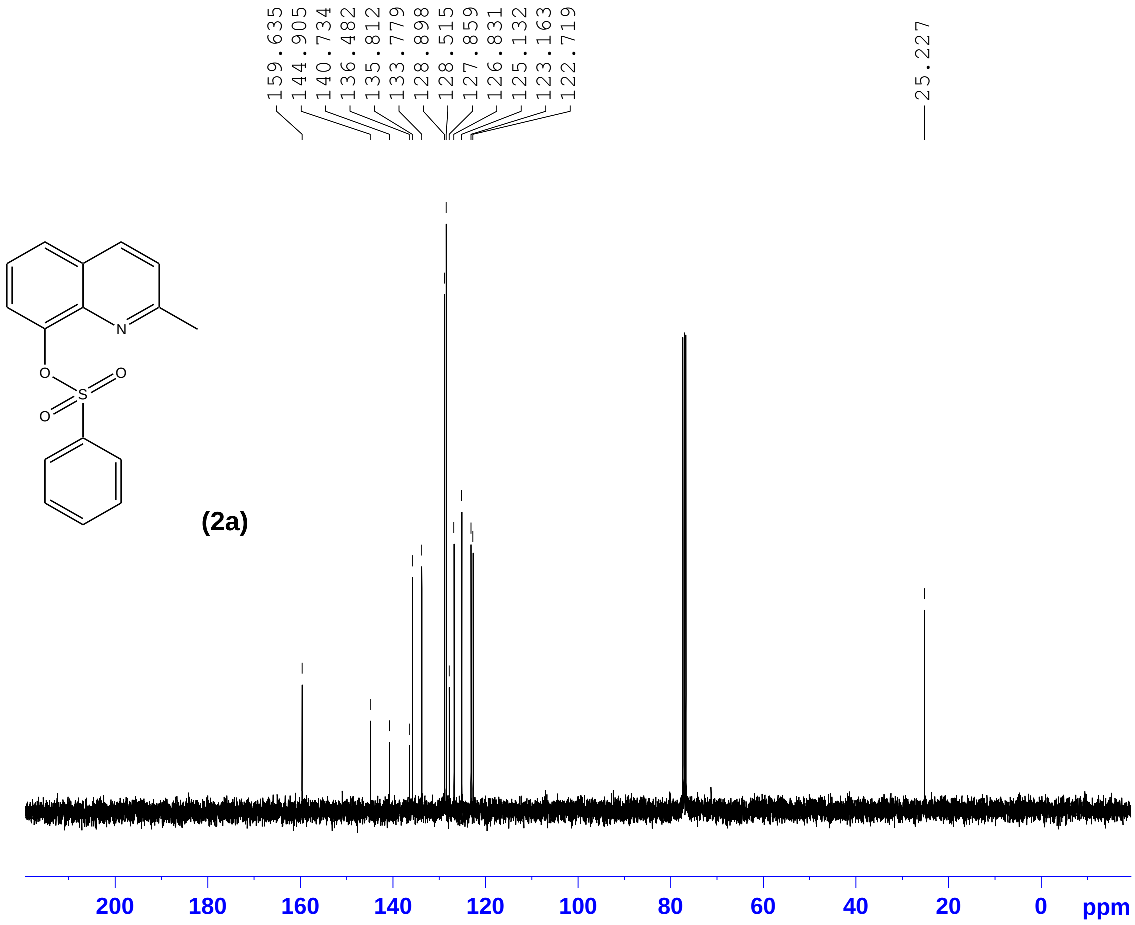

Figure S9. ^{1}H (400 MHz) and ^{13}C (100 MHz) NMR spectra of **2a** in $CDCl_3$.

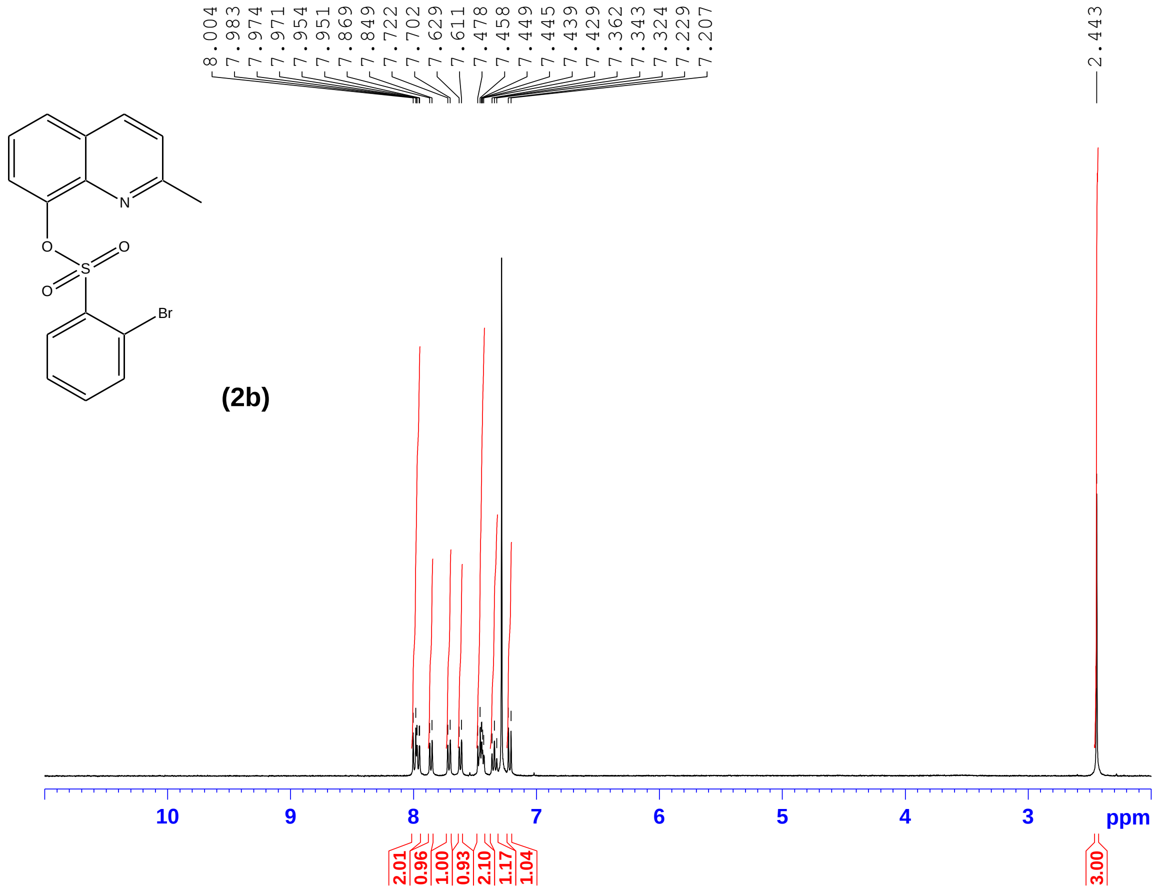

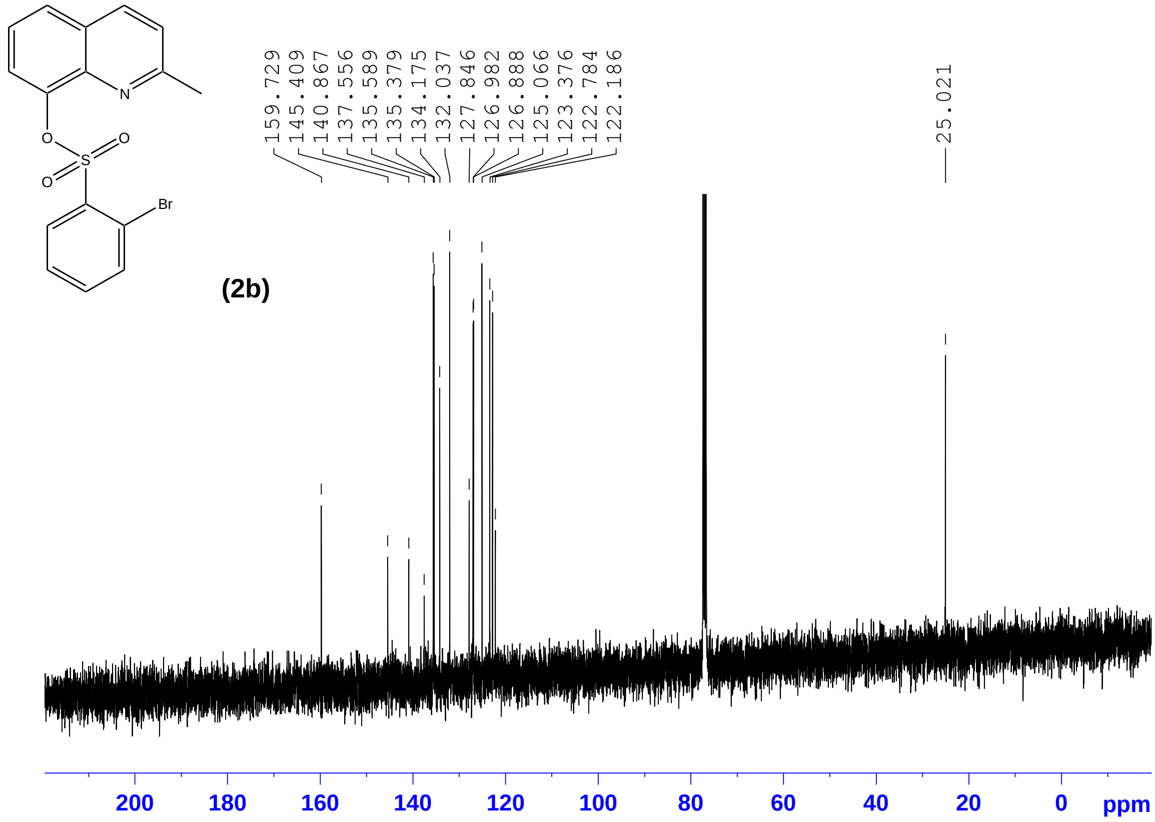

Figure S10. ^{1}H (400 MHz) and ^{13}C (100 MHz) NMR spectra of **2b** in $CDCl_3$.

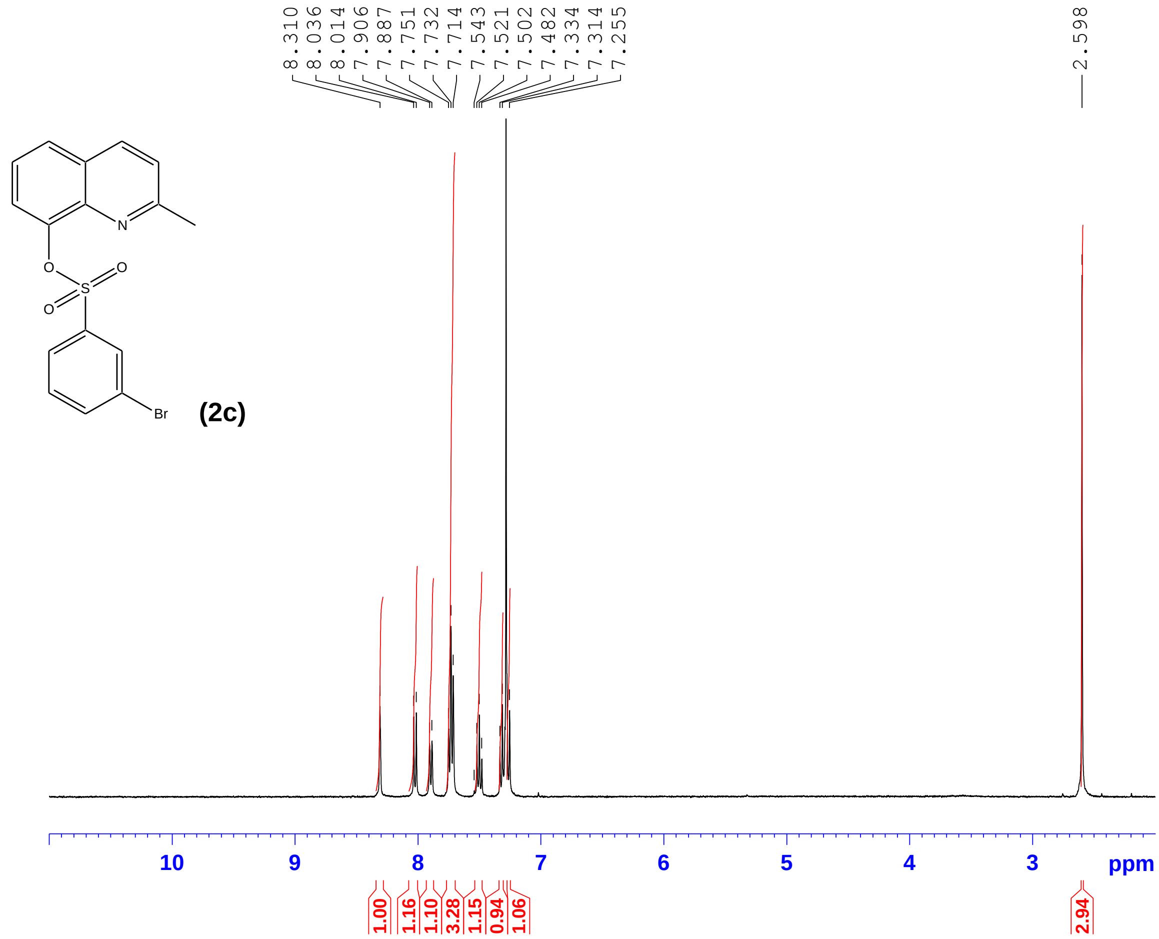

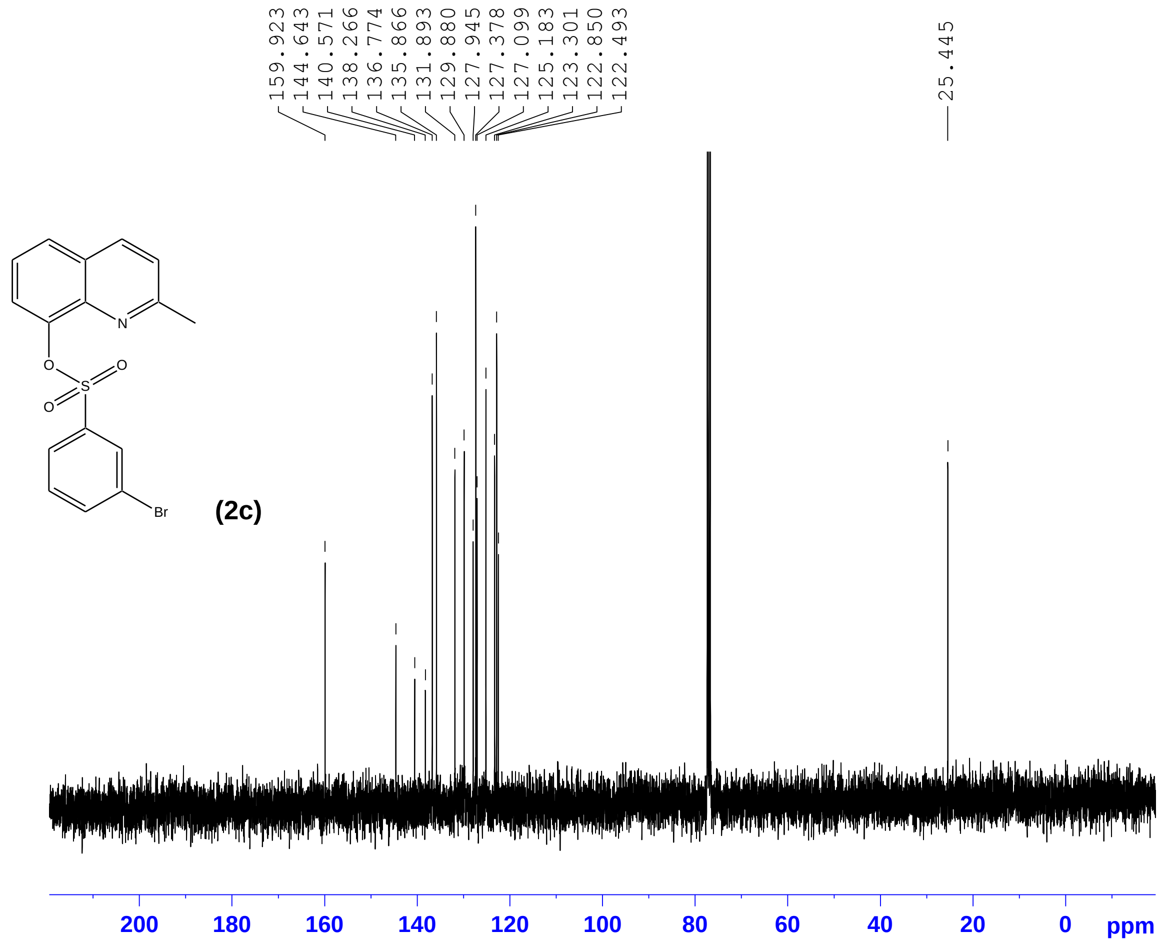

Figure S11. ^{1}H (400 MHz) and ^{13}C (100 MHz) NMR spectra of **2c** in $CDCl_3$.

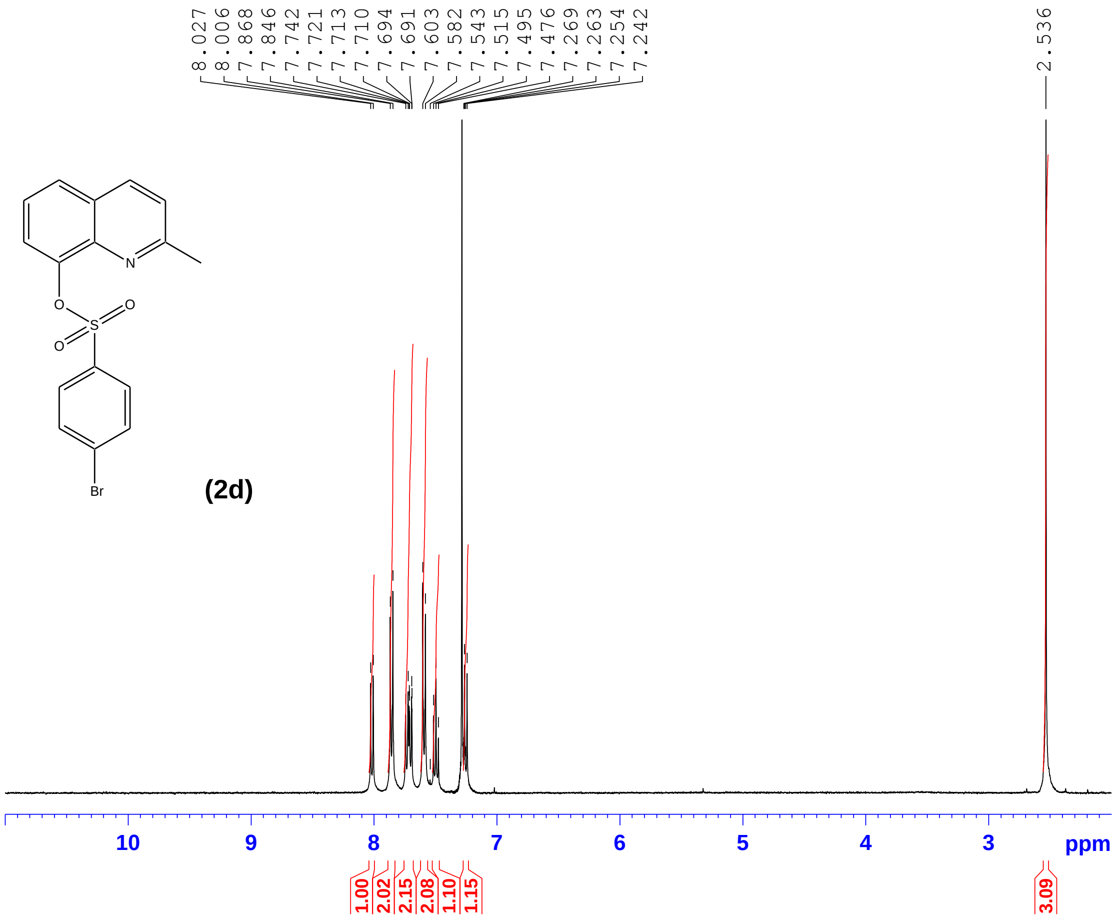

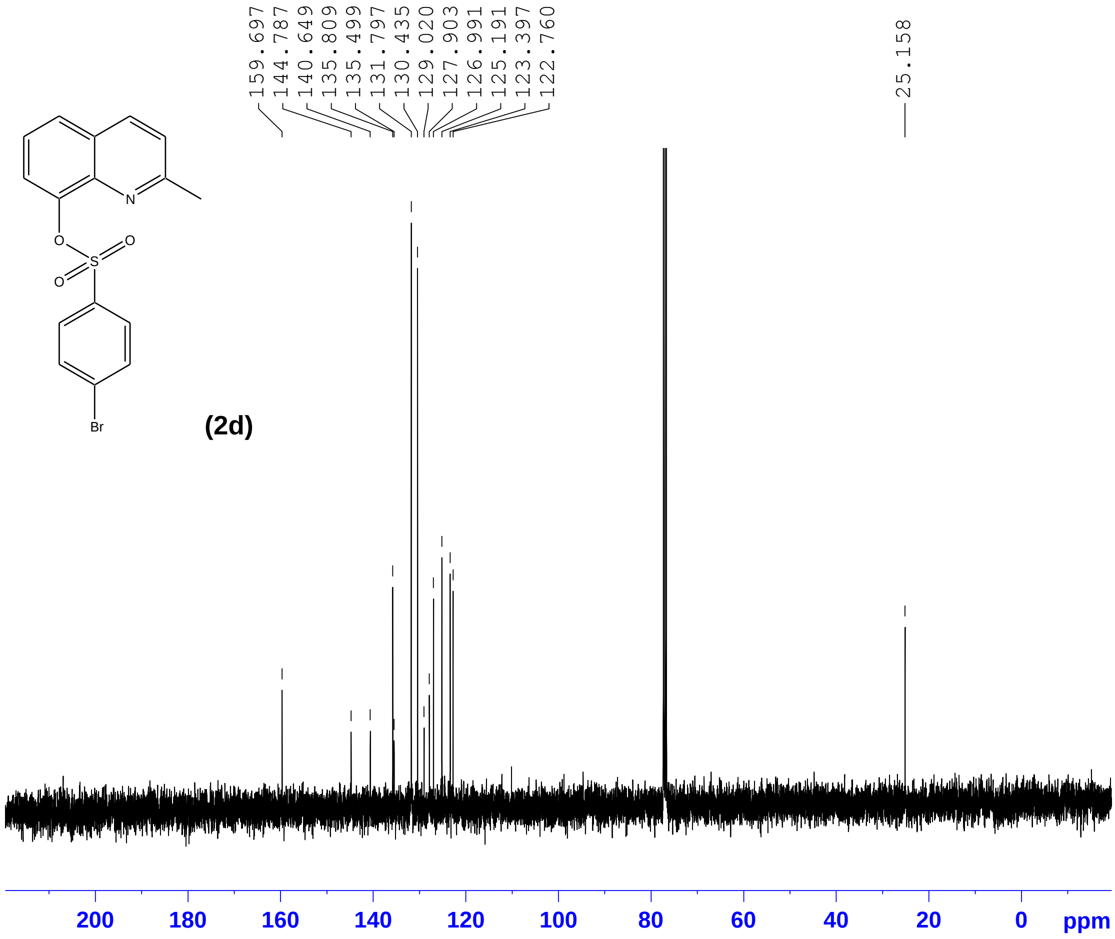

Figure S12. ^{1}H (400 MHz) and ^{13}C (100 MHz) NMR spectra of **2d** in $CDCl_3$.

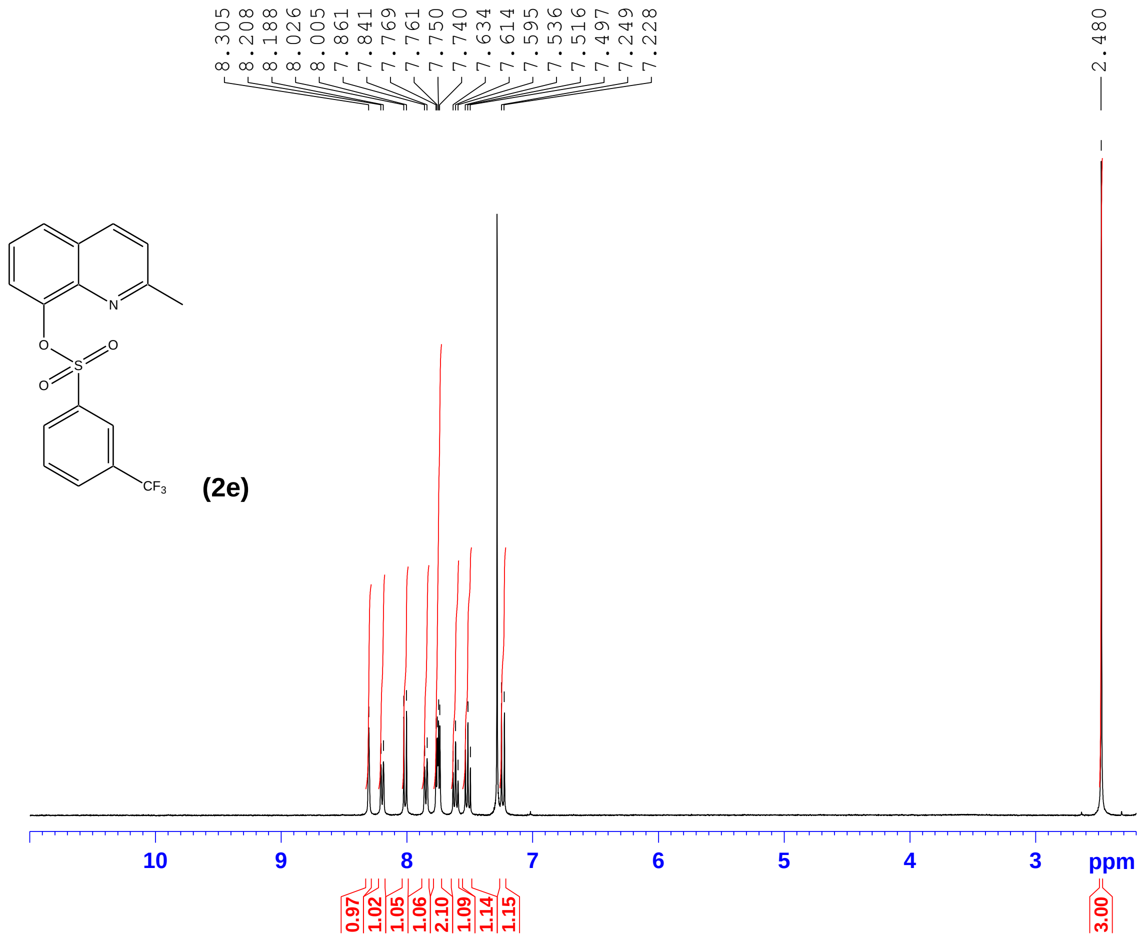

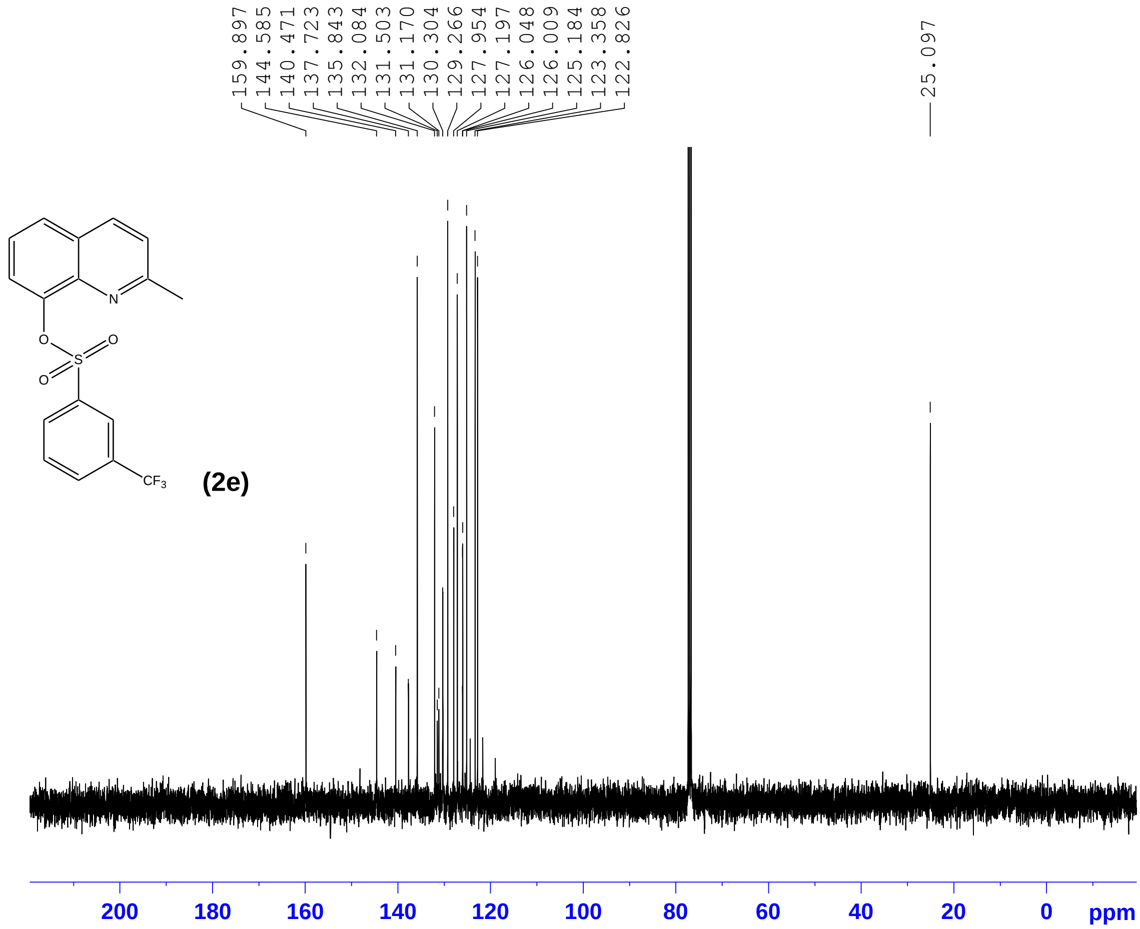

Figure S13. ^{1}H (400 MHz) and ^{13}C (100 MHz) NMR spectra of **2e** in $CDCl_3$.

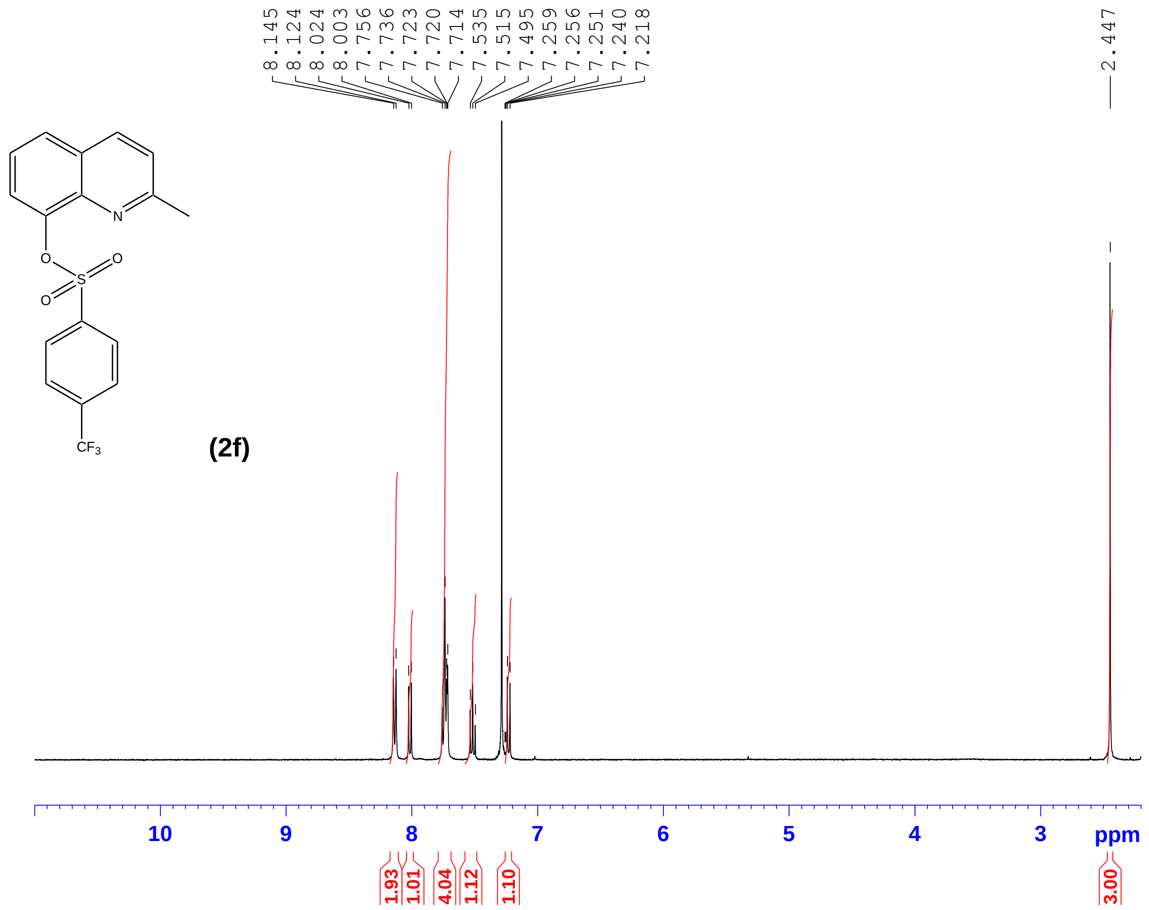

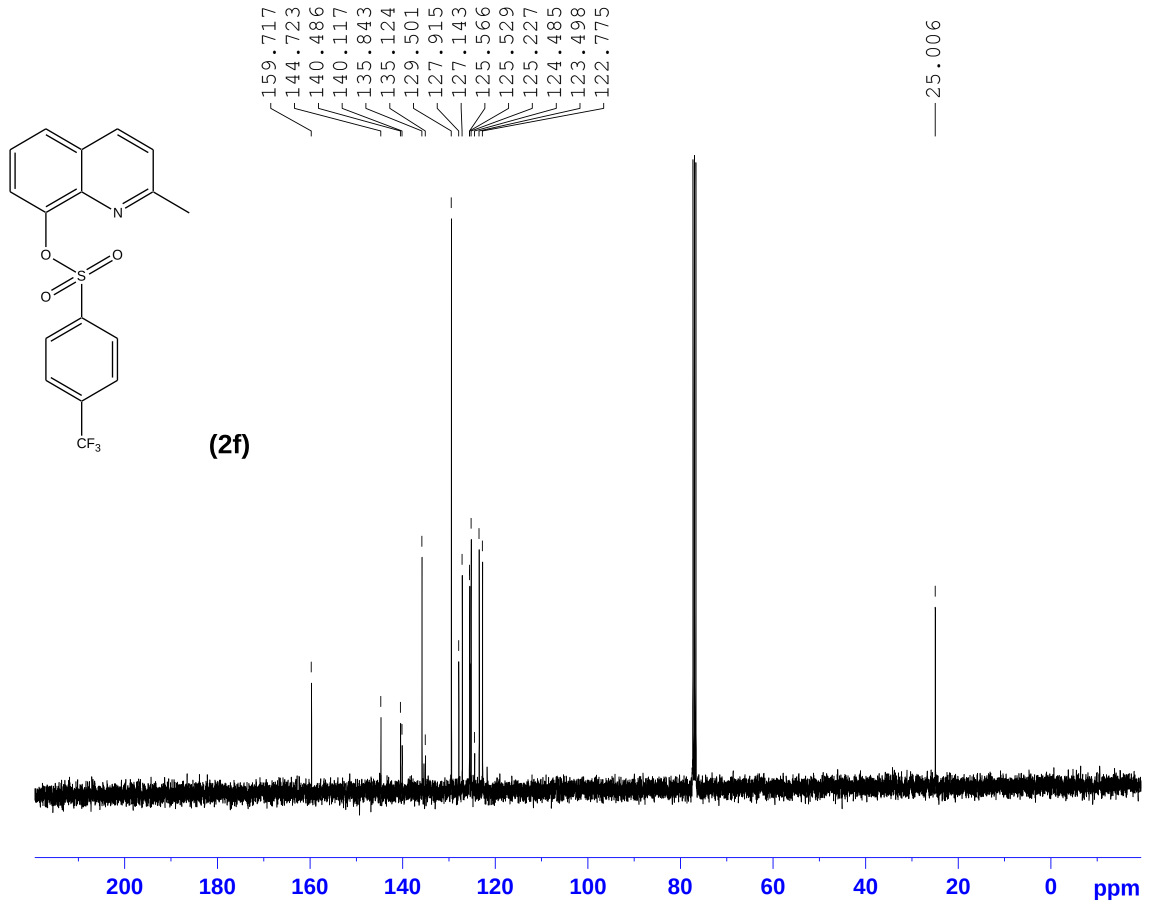

Figure S14. ^{1}H (400 MHz) and ^{13}C (100 MHz) NMR spectra of **2f** in $CDCl_3$.

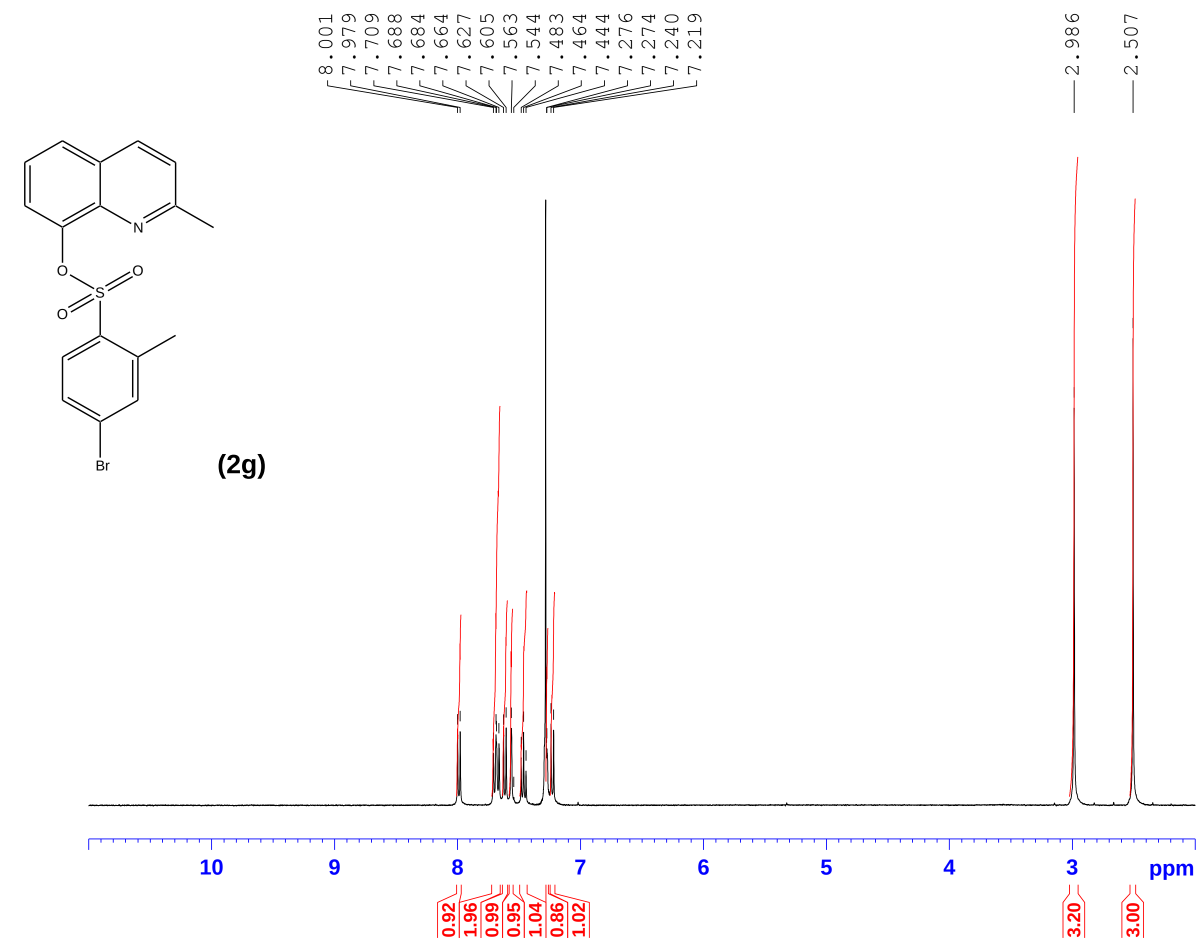

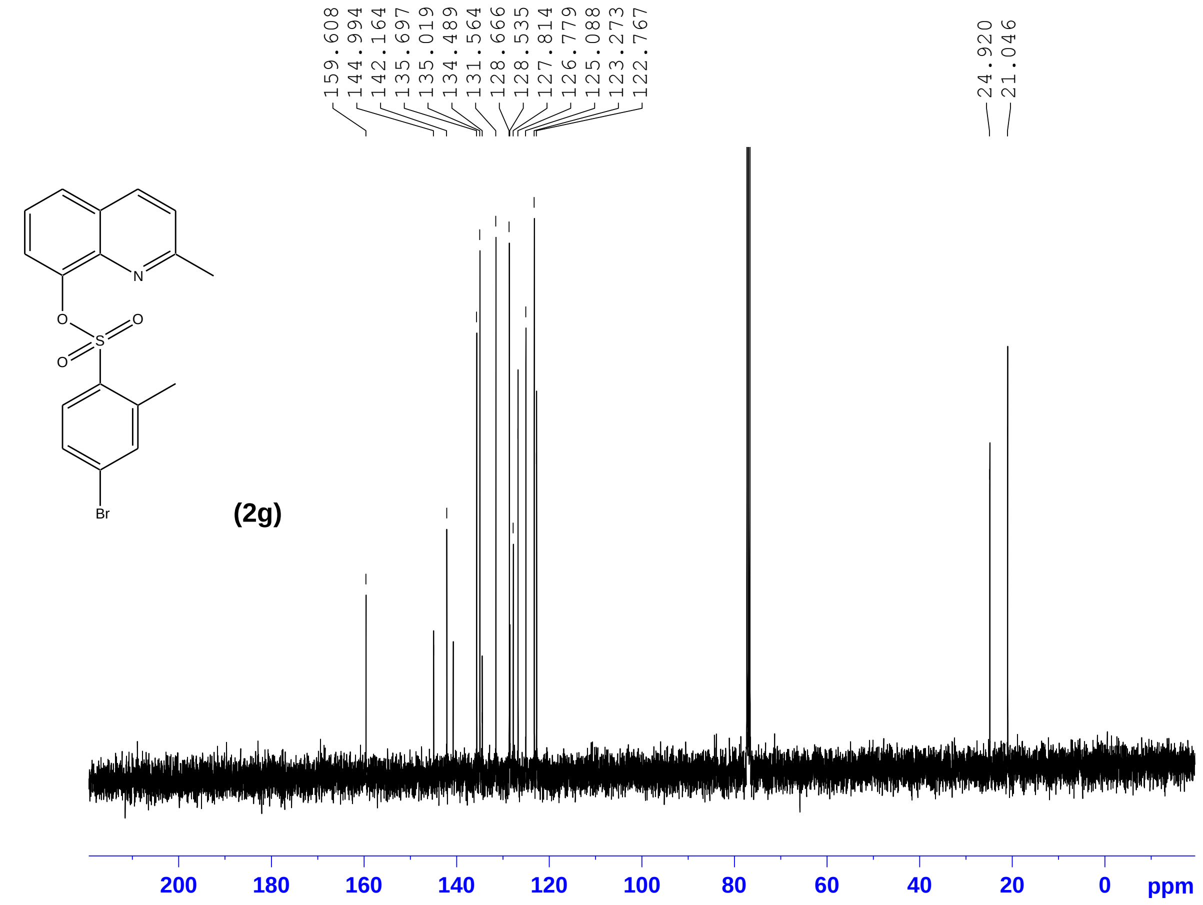

Figure S15. ^{1}H (400 MHz) and ^{13}C (100 MHz) NMR spectra of **2g** in $CDCl_3$.

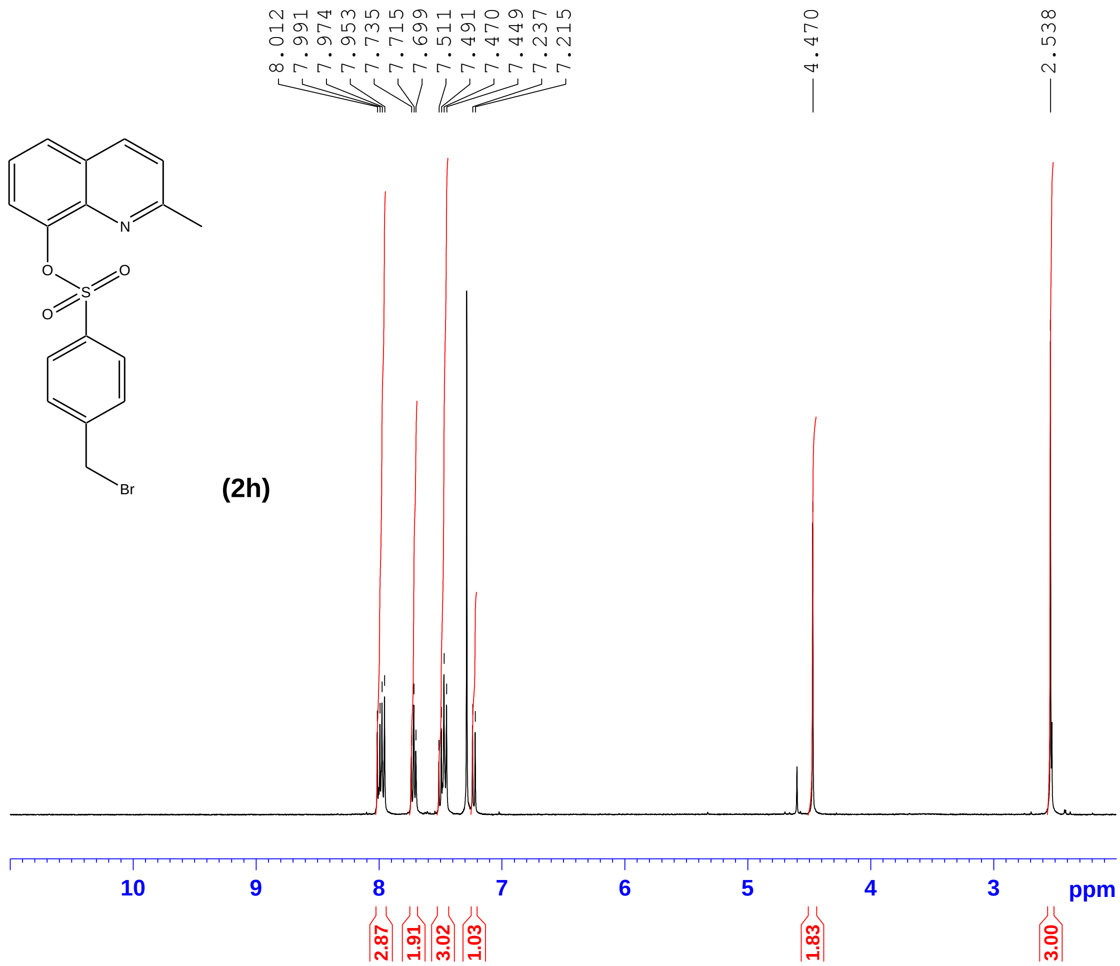

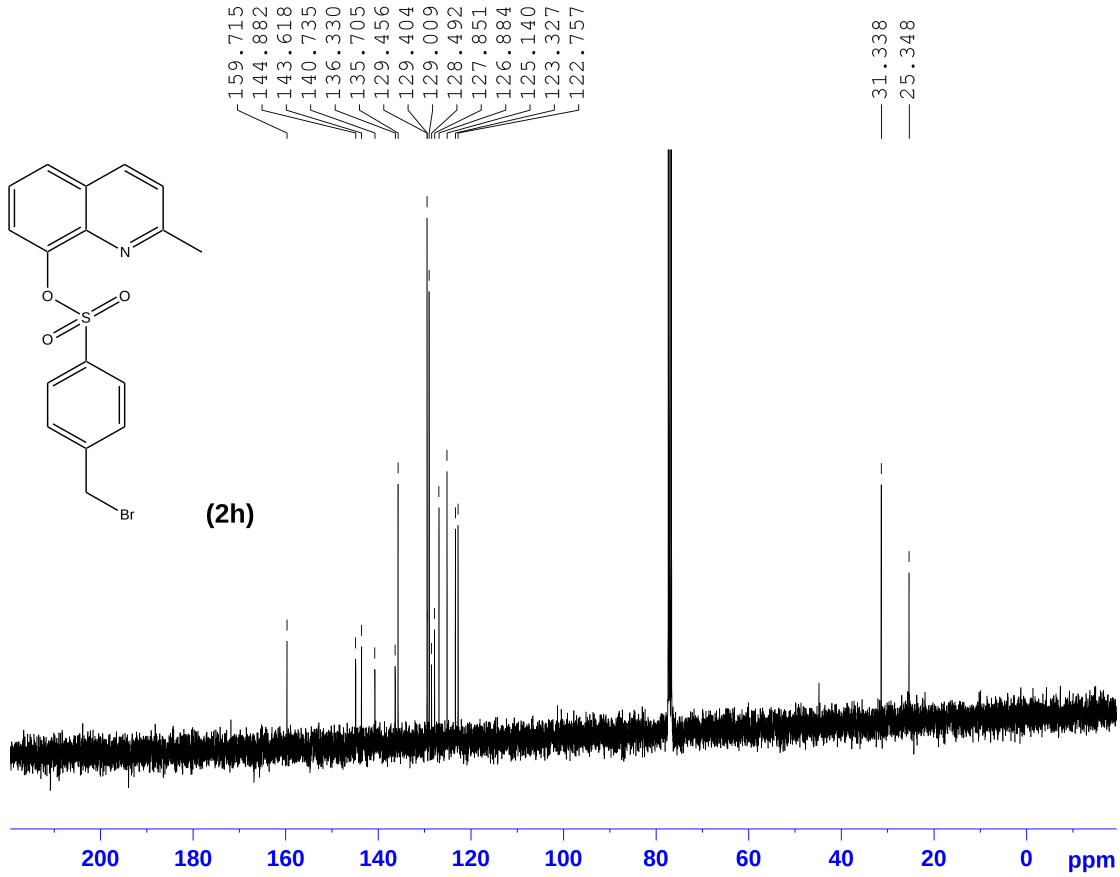

Figure S16. ^{1}H (400 MHz) and ^{13}C (100 MHz) NMR spectra of **2h** in $CDCl_3$.

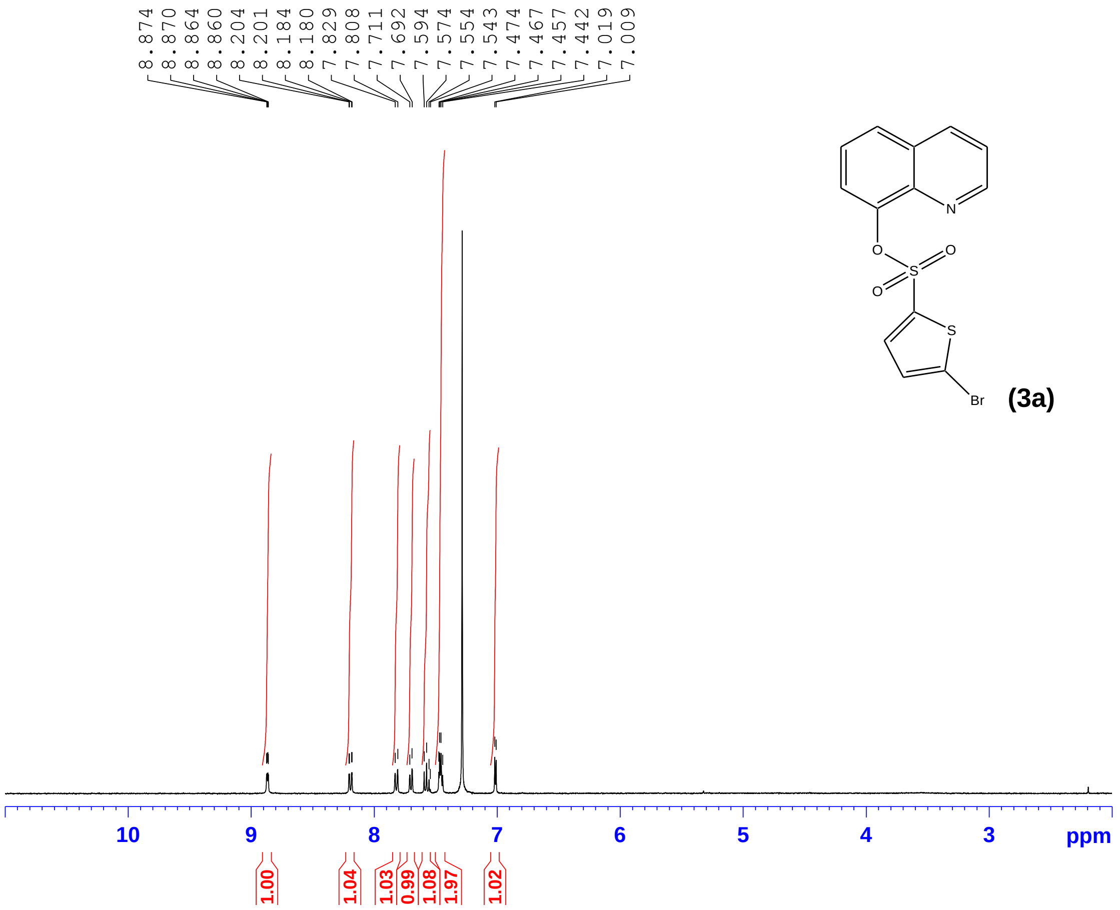

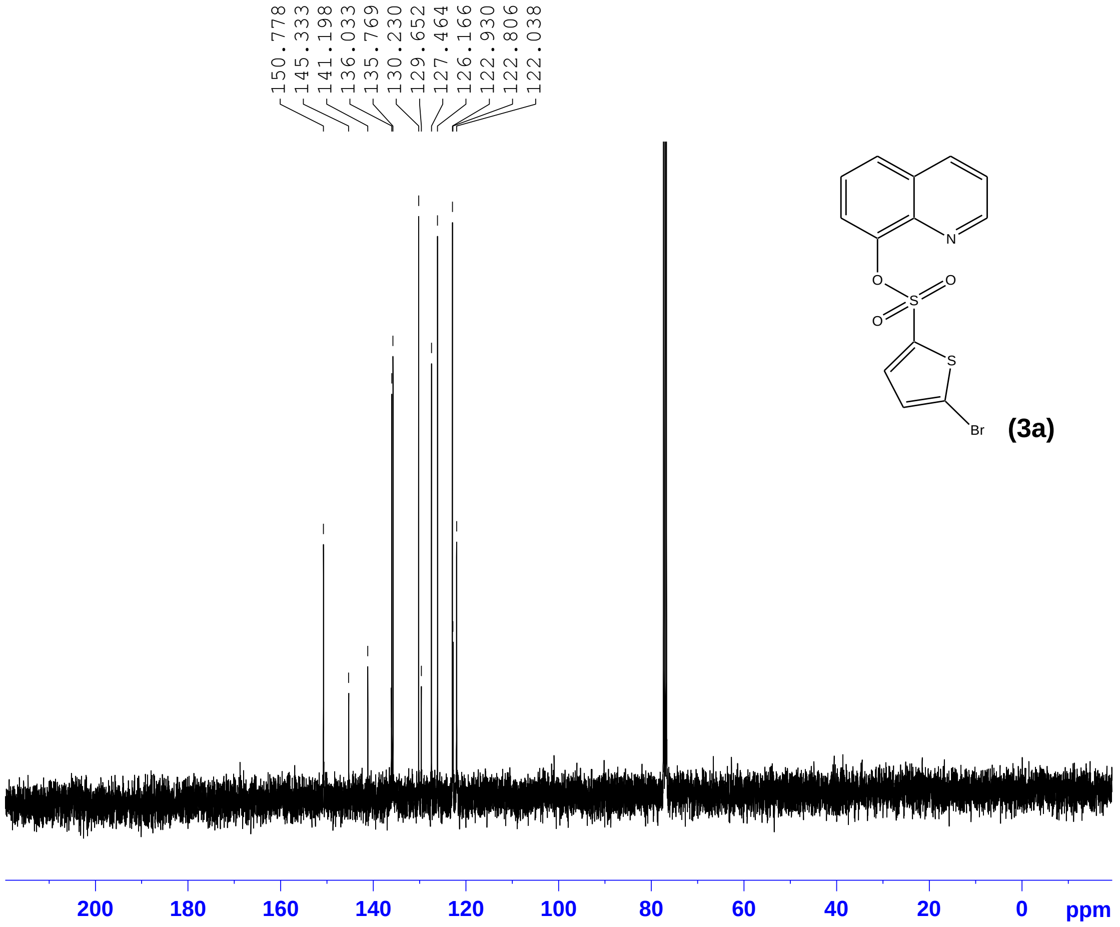

Figure S17. ^{1}H (400 MHz) and ^{13}C (100 MHz) NMR spectra of **3a** in $CDCl_3$.

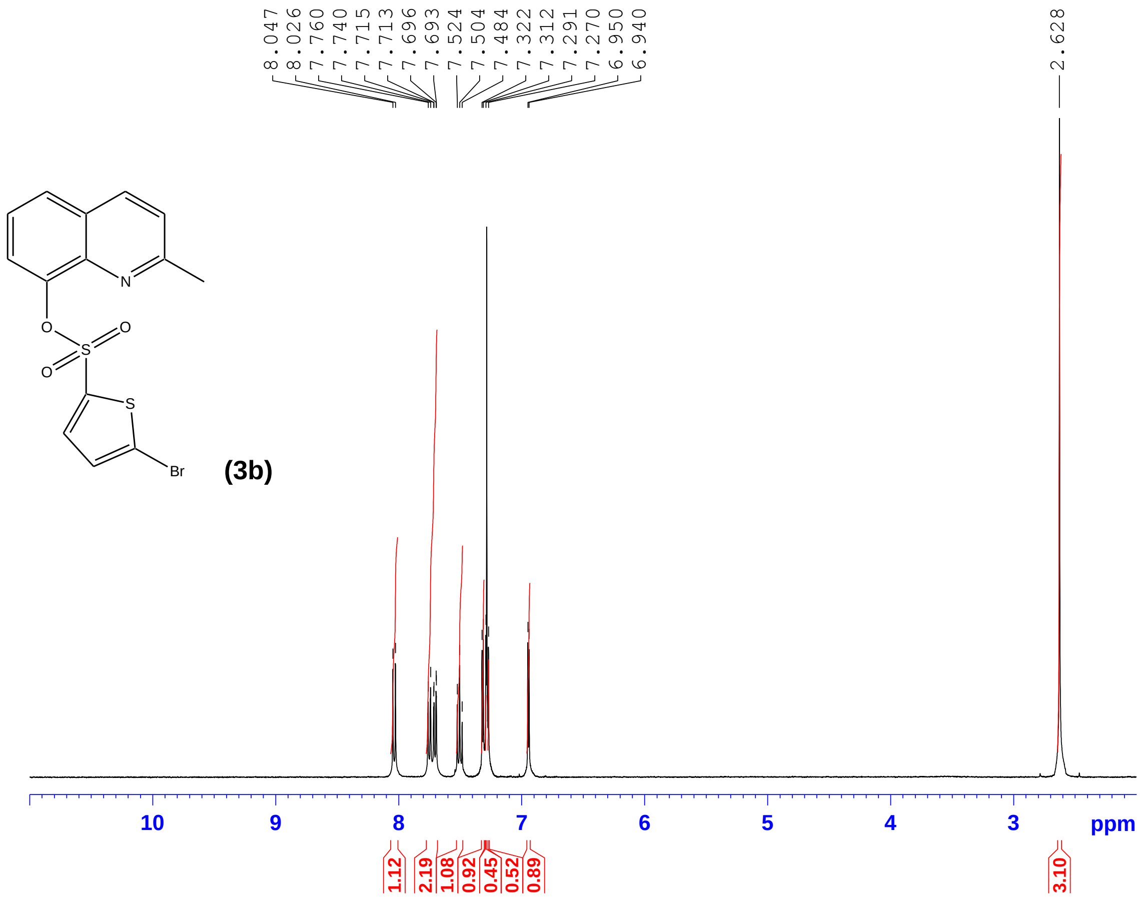

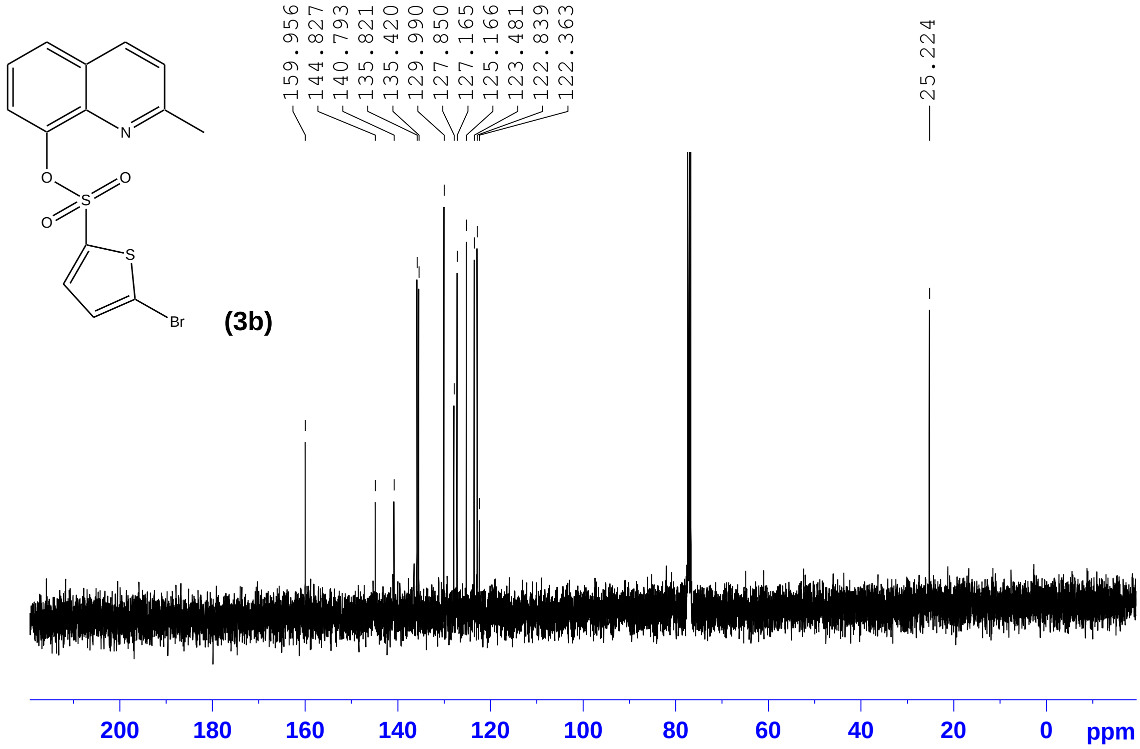

Figure S18. ^{1}H (400 MHz) and ^{13}C (100 MHz) NMR spectra of **3b** in $CDCl_3$.

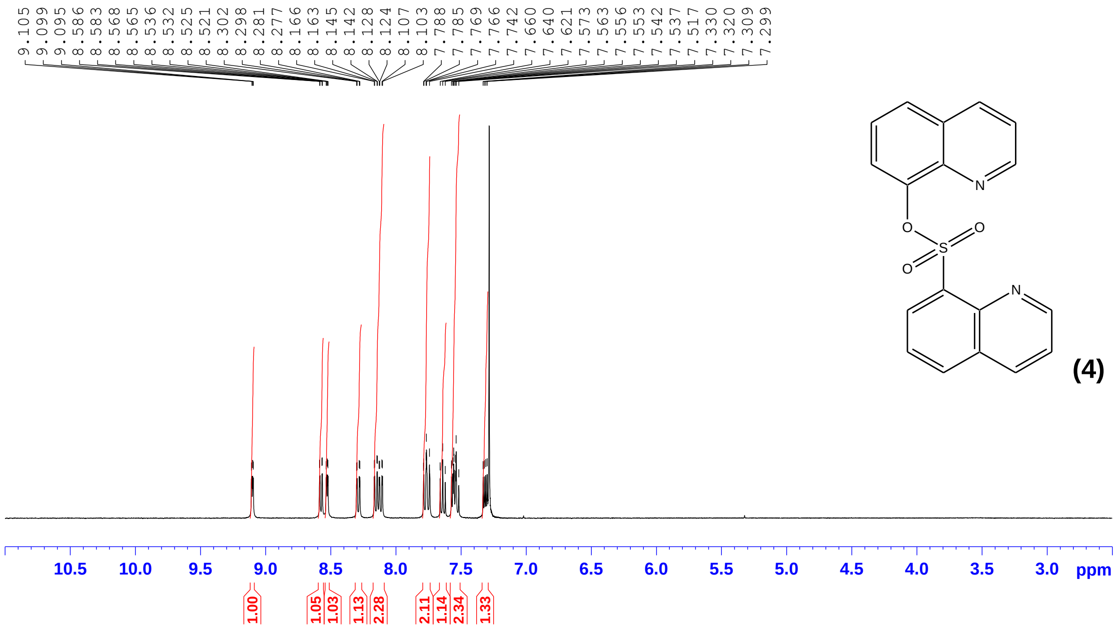

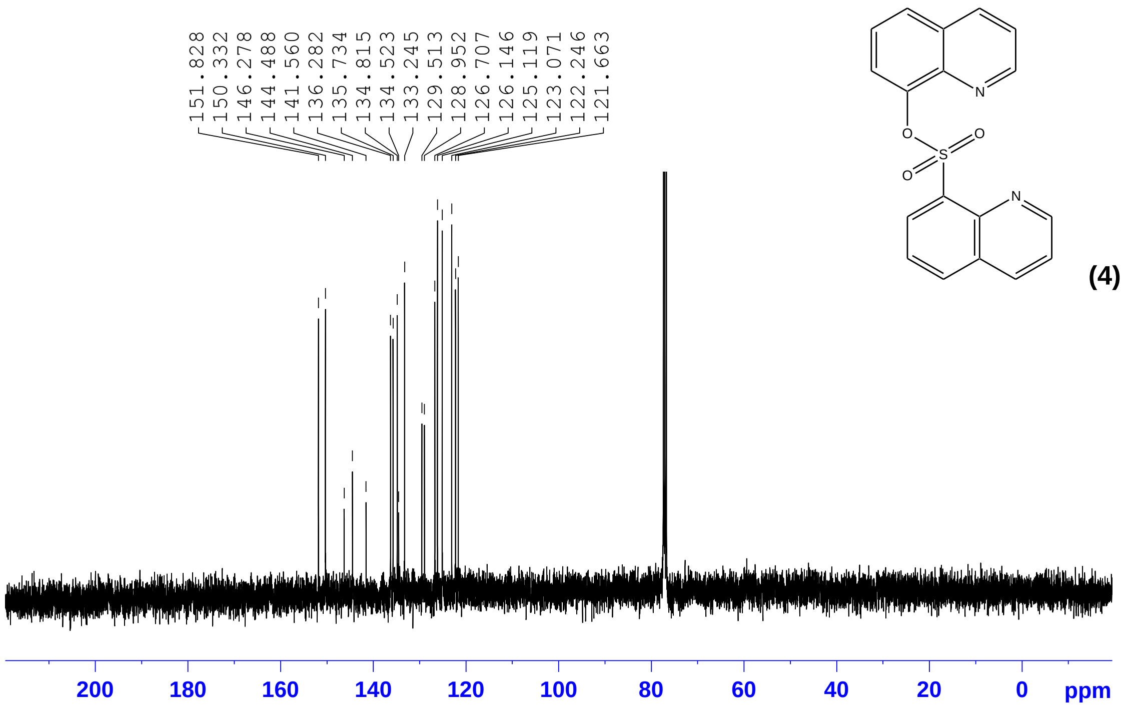

Figure S19. ^{1}H (400 MHz) and ^{13}C (100 MHz) NMR spectra of **4** in $CDCl_3$.

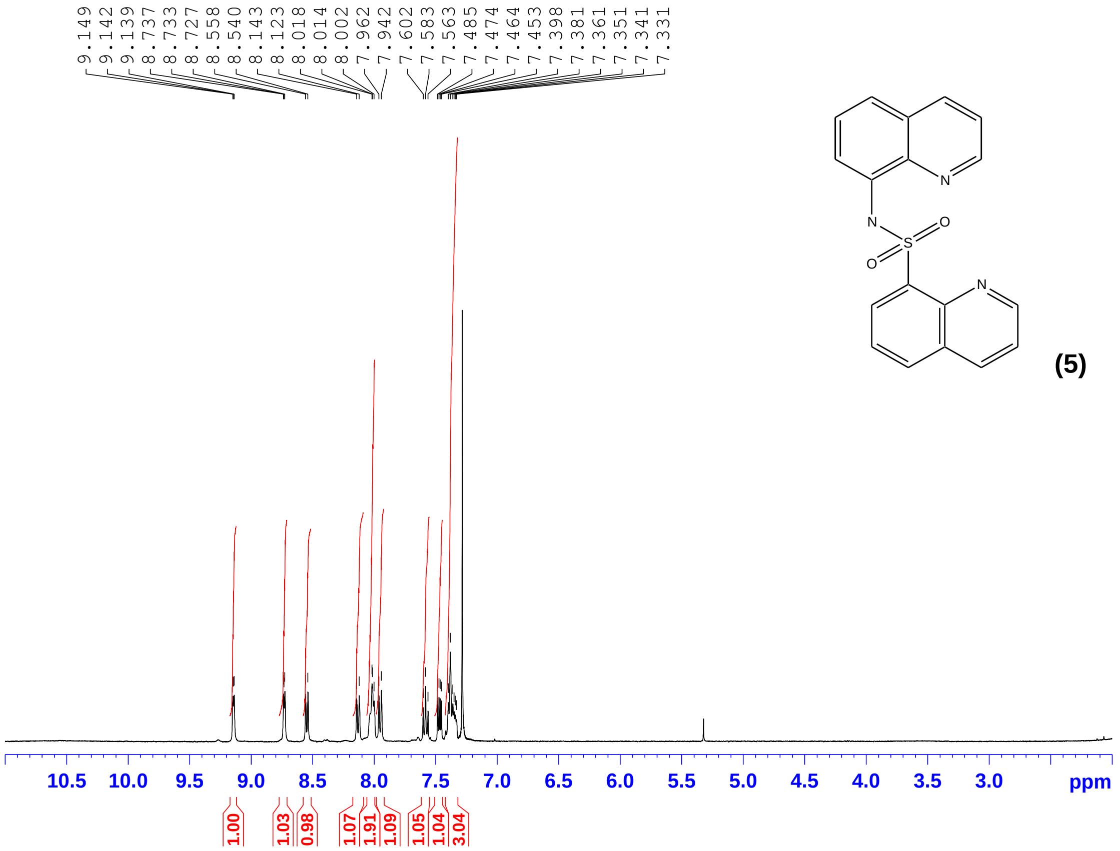

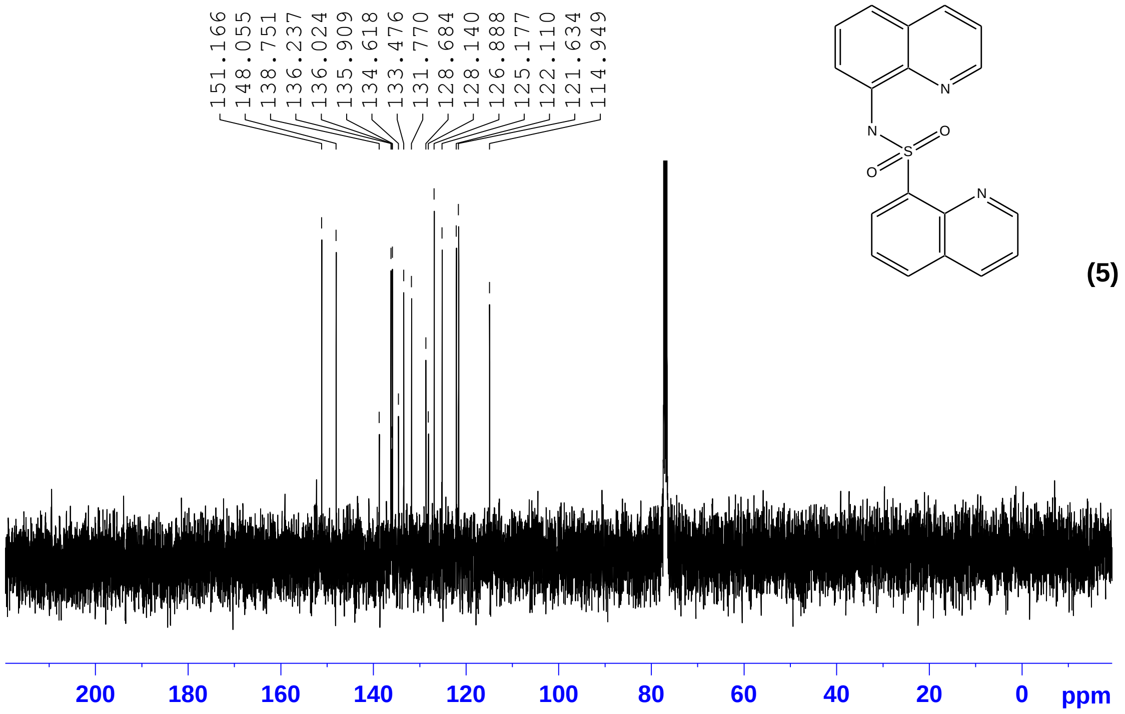

Figure S20. ^{1}H (400 MHz) and ^{13}C (100 MHz) NMR spectra of **5** in $CDCl_3$.

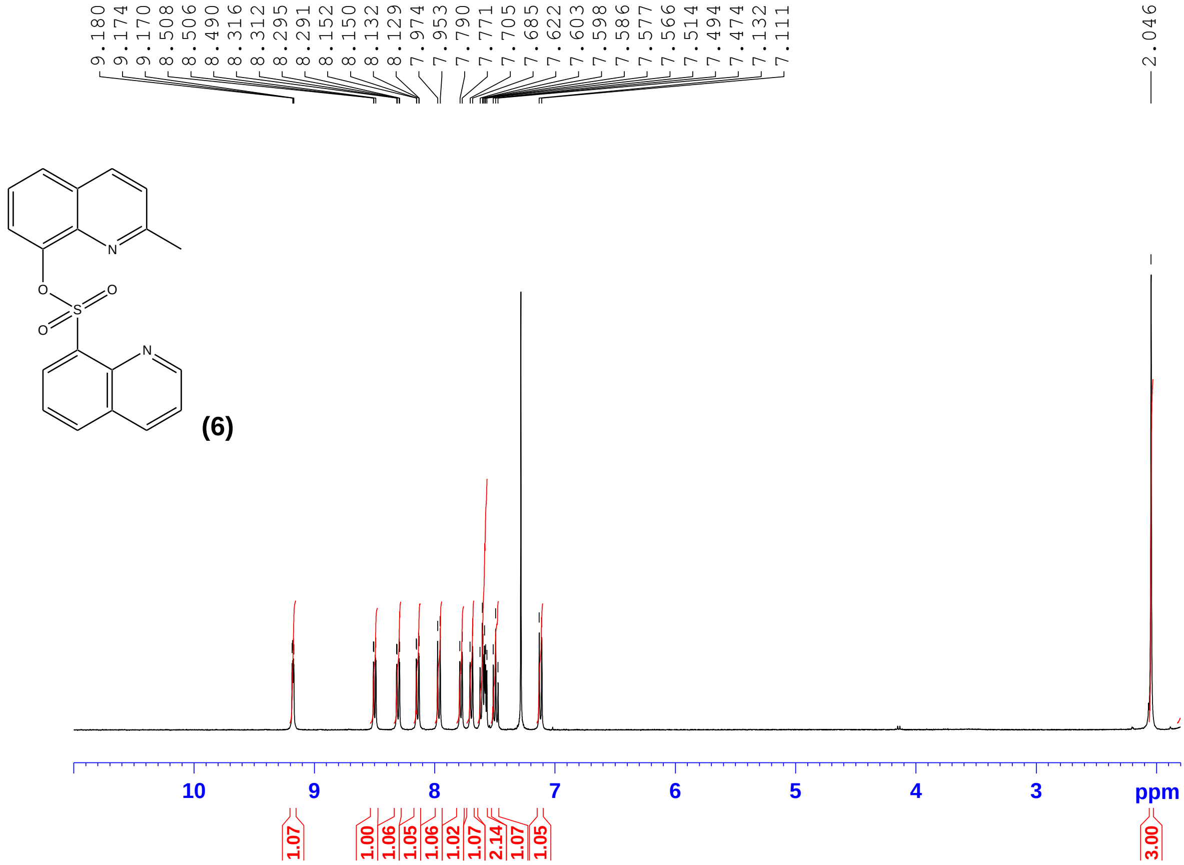

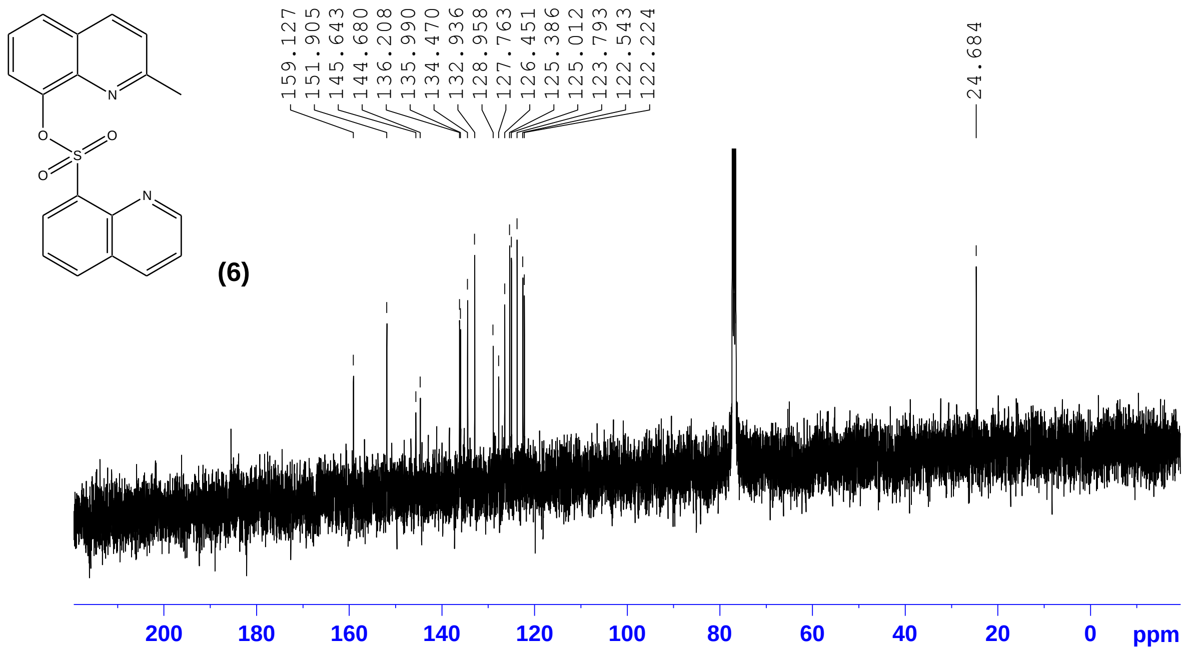

Figure S21. ^{1}H (400 MHz) and ^{13}C (100 MHz) NMR spectra of **6** in $CDCl_3$.

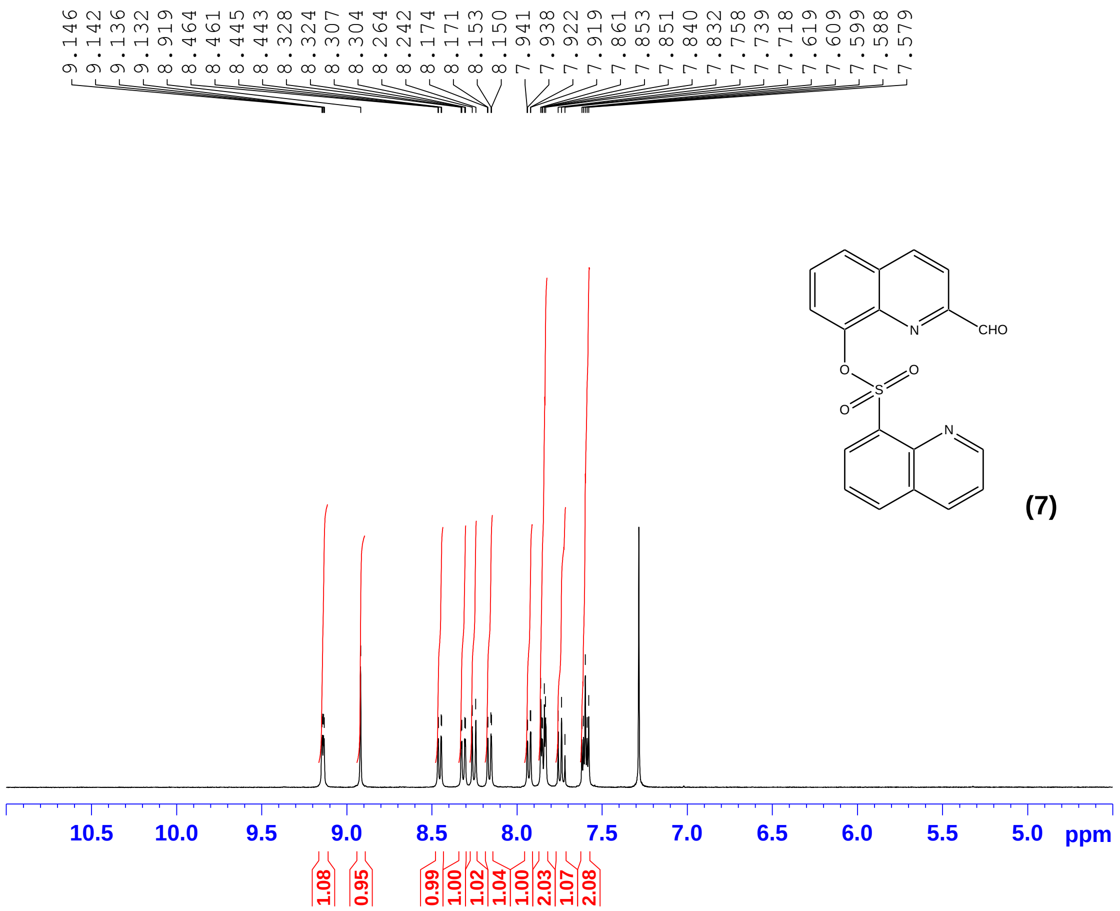

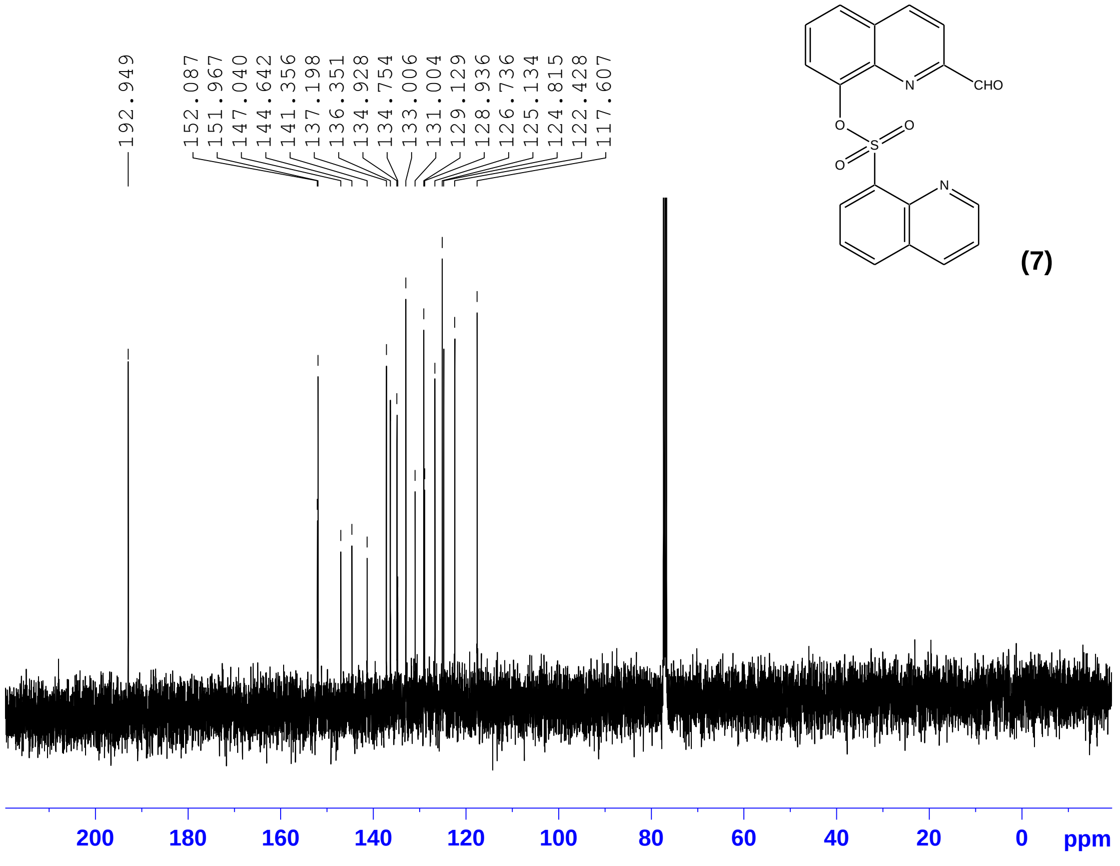

Figure S22. ^{1}H (400 MHz) and ^{13}C (100 MHz) NMR spectra of **7** in $CDCl_3$.

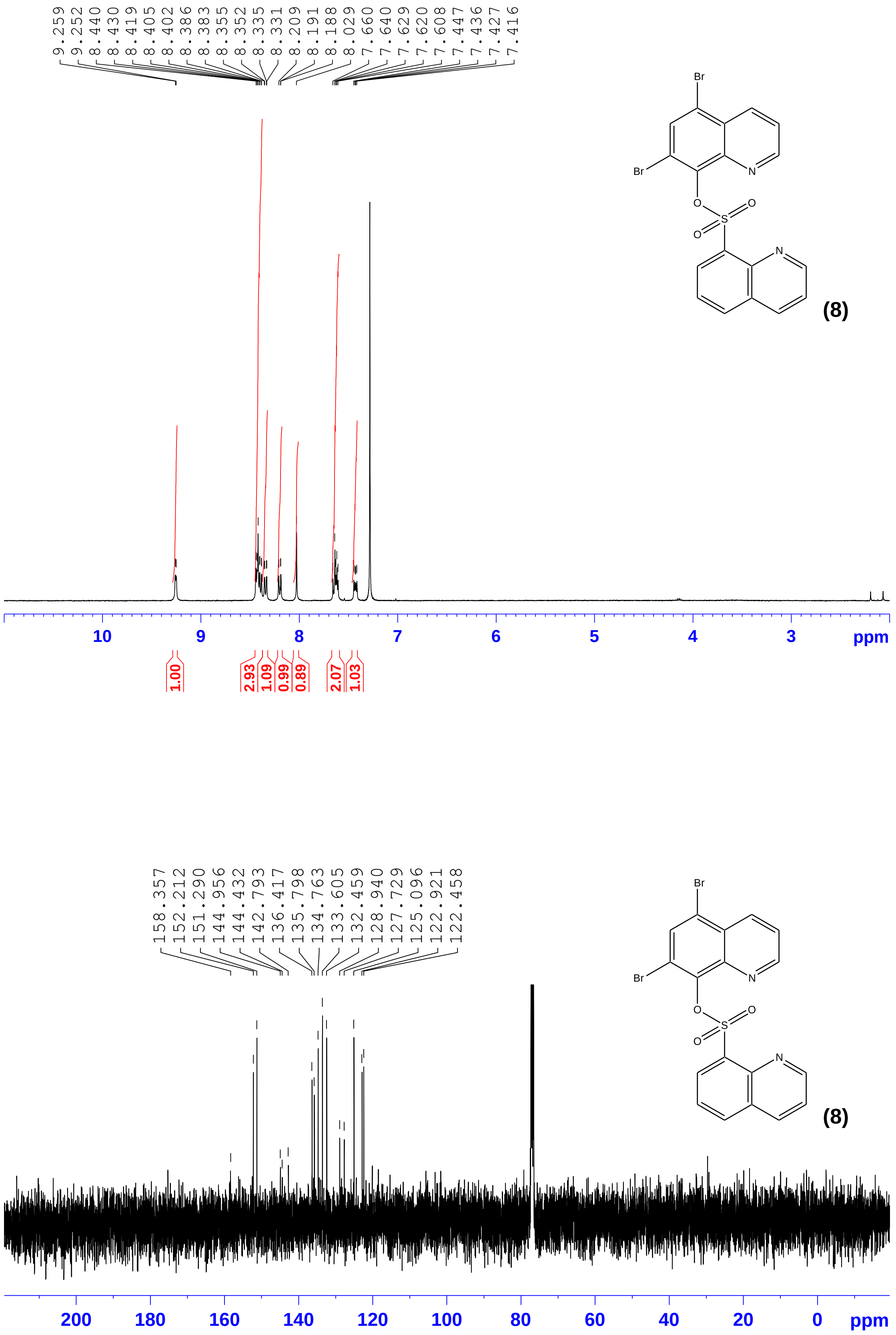

Figure S23. ^{1}H (400 MHz) and ^{13}C (100 MHz) NMR spectra of **8** in $CDCl_3$.

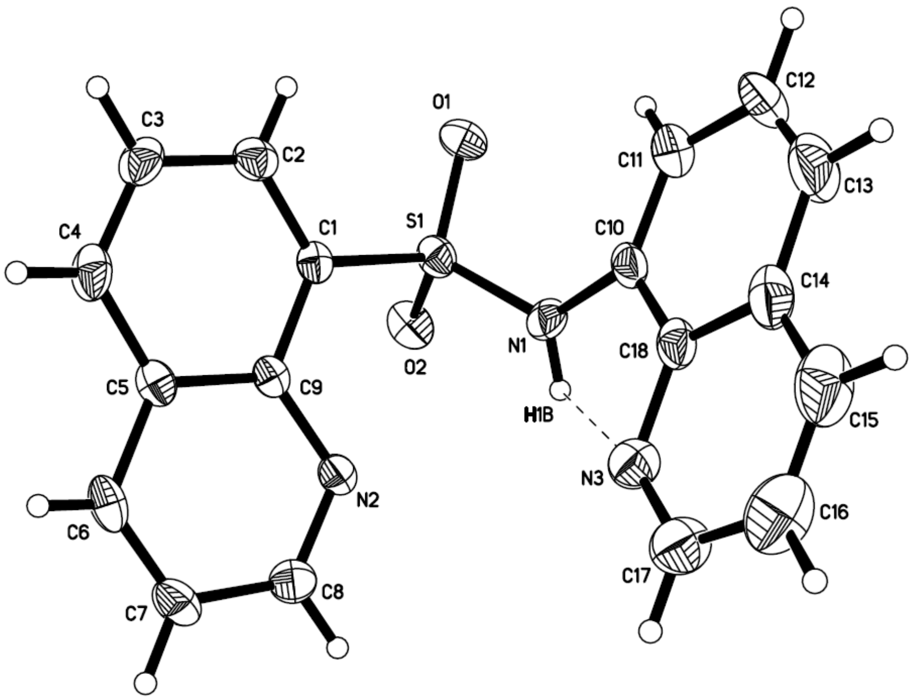

Figure S24. Molecular structure of **5** obtained from X-ray crystallography.
